# *Plasmodium falciparum* contains functional SCF and CRL4 ubiquitin E3 ligases, and CRL4 is critical for cell division and membrane integrity

**DOI:** 10.1371/journal.ppat.1012045

**Published:** 2024-02-28

**Authors:** Zeba Rizvi, G. Srinivas Reddy, Somesh M. Gorde, Priyanka Pundir, Divya Das, Puran Singh Sijwali

**Affiliations:** 1 CSIR-Centre for Cellular and Molecular Biology, Hyderabad-500007, India; 2 Academy of Scientific and Innovative Research (AcSIR), Ghaziabad-201002, UP, India; University of Texas Southwestern Medical Center at Dallas, UNITED STATES

## Abstract

Protein ubiquitination is essential for cellular homeostasis and regulation of several processes, including cell division and genome integrity. Ubiquitin E3 ligases determine substrate specificity for ubiquitination, and Cullin-RING E3 ubiquitin ligases (CRLs) make the largest group among the ubiquitin E3 ligases. Although conserved and most studied in model eukaryotes, CRLs remain underappreciated in *Plasmodium* and related parasites. To investigate the CRLs of human malaria parasite *Plasmodium falciparum*, we generated parasites expressing tagged *P*. *falciparum* cullin-1 (PfCullin-1), cullin-2 (PfCullin-2), Rbx1 (PfRbx1) and Skp1 (PfSkp1). PfCullin-1 and PfCullin-2 were predominantly expressed in erythrocytic trophozoite and schizont stages, with nucleocytoplasmic localization and chromatin association, suggesting their roles in different cellular compartments and DNA-associated processes. Immunoprecipitation, *in vitro* protein-protein interaction, and ubiquitination assay confirmed the presence of a functional Skp1-Cullin-1-Fbox (PfSCF) complex, comprising of PfCullin-1, PfRbx1, PfSkp1, PfFBXO1, and calcyclin binding protein. Immunoprecipitation, sequence analysis, and ubiquitination assay indicated that PfCullin-2 forms a functional human CRL4-like complex (PfCRL4), consisting of PfRbx1, cleavage and polyadenylation specificity factor subunit_A and WD40 repeat proteins. PfCullin-2 knock-down at the protein level, which would hinder PfCRL4 assembly, significantly decreased asexual and sexual erythrocytic stage development. The protein levels of several pathways, including protein translation and folding, lipid biosynthesis and transport, DNA replication, and protein degradation were significantly altered upon PfCullin-2 depletion, which likely reflects association of PfCRL4 with multiple pathways. PfCullin-2-depleted schizonts had poorly delimited merozoites and internal membraned structures, suggesting a role of PfCRL4 in maintaining membrane integrity. PfCullin-2-depleted parasites had a significantly lower number of nuclei/parasite than the normal parasites, indicating a crucial role of PfCRL4 in cell division. We demonstrate the presence of functional CRLs in *P*. *falciparum*, with crucial roles for PfCRL4 in cell division and maintaining membrane integrity.

## Author summary

*Plasmodium falciparum* is the major contributor to global burden of malaria. The parasite produces and disposes a number of its cellular contents during its development, which would require degradation machineries. Ubiquitin proteasome system (UPS) is a major degradation machinery in eukaryotes, which tags proteins by ubiquitin and degrades the tagged-proteins. Ubiquitin E3 ligases recognize the proteins to be tagged with ubiquitin, and Cullin-RING E3 ubiquitin ligases (CRLs) make the largest group of these enzymes. CRLs have crucial roles in a variety of processes and are also targets of ongoing drug discovery projects. Hence, we investigated CRLs of *P*. *falciparum*, and show that it has two functional CRLs: PfSCF and PfCRL4. Both these enzymes are present during parasite’s development in erythrocytes, the malaria-causing phase of the parasite. Both the enzymes are present in cytoplasmic and nuclear compartments, suggesting roles in these two compartments. Depletion of PfCullin-2, a constituent of PfCRL4, at protein level decreased parasite growth during disease-causing and transmission phases. The depletion also caused damage to parasite membranes and blocked nuclear division, indicating a crucial role of PfCRL4. Overall, we have identified two major enzymes of the parasite UPS, which could be used as drug targets.

## Introduction

*Plasmodium* species cause malaria in humans and several other vertebrates. Human malaria is a huge burden, with the latest estimates of about 247 million cases and 6,19,000 deaths in 2021 [1*]*. *Plasmodium* species undergo multi-stage development in diverse cellular environments, with massive changes in the stage-specific proteomes and organelles, which must require protein turnover pathways like the ubiquitin proteasome system (UPS) and autophagy. UPS has key roles in diverse cellular processes, including cell cycle progression, differentiation, endocytic transport, transcription, DNA repair, signalling, and antigen presentation [2–8]. *Plasmodium* UPS is a validated drug target, and its inhibition potentiates the antimalarial activity of artemisinin on artemisinin susceptible and resistant *P*. *falciparum* strains, which further strengthens the therapeutic potential and warrants investigation of *Plasmodium* UPS [9–20].

The core UPS components are ubiquitin, ubiquitination enzymes, and proteasome. Ubiquitination, the covalent conjugation of ubiquitin to the ε-amino group of a lysine residue of the substrate protein, involves sequential action of ubiquitin-activating enzyme E1, transfer of the activated ubiquitin to ubiquitin-conjugating enzyme E2, followed by transfer of ubiquitin from the E2-ubiquitin conjugate to the substrate lysine residue by ubiquitin E3 ligase. Finally, ubiquitinated proteins are degraded by the proteasome. However, ubiquitination also serves as a non-degradation signal, which depends on the ubiquitination chain topology [21].

Ubiquitin E3 ligases recognize substrate proteins, thereby dictating target specificity of the UPS. There are 600–700 putative ubiquitin E3 ligases in humans, which are classified into RING E3s, HECT E3s, and RBR E3s depending on the domain organization and the mechanism of ubiquitin transfer to the substrate [22,23]. RING E3s contain structurally similar RING (Really Interesting New Gene) or U-box domain proteins, which bind the E2-ubiquitin conjugate and facilitate ubiquitin transfer to the substrate. HECT E3s contain a characteristic HECT domain (homologous to the E6AP carboxyl terminus) and catalyse ubiquitin transfer to their own catalytic cysteine residue and then to the substrate. RBR (RING-in-between-RING) E3s contain a canonical RING finger domain (RING1), an In-Between-RING domain (IBR) and a RING2 domain. The RING1 domain accepts the E2-ubiquitin conjugate and mediates transfer of ubiquitin to the catalytic cysteine residue in RING2, which then transfers ubiquitin to the substrate. Some of the best studied RING E3s are Cullin-RING E3 ubiquitin ligases (CRLs), which make about 40% of the total ubiquitin E3 ligases. CRLs contain a cullin subunit that serves as a scaffold for assembly of the complex, a RING-box protein that binds E2 of the E2-ubiquitin conjugate, and a substrate adaptor for recruitment of the substrate. Skp1-Cullin-1-F-box (SCF) and anaphase-promoting complex/cyclosome (APC/C) are the two most notable CRLs, which mediate timely degradation of cell cycle regulatory proteins [24,25]. Cullin proteins are evolutionary conserved, with 8 in humans (Cul1-3, 4A, 4B, 5, 7 and 9/PARC) and 3 in *S*. *cerevisiae* (cul1, cul3, cul8) [26–28]. CRLs have been mostly named based on the cullin subunit present in the complex; for example, cullin-1 makes CRL1 that is also known as SCF, and Cullin-4A and Cullin-4B make CRL4A and CRL4B, respectively.

CRLs have been shown to have key roles in cell cycle regulation [29], DNA replication [30], and DNA damage repair [31]. A key regulatory role of SCF and APC/C is timely degradation of cyclins and cyclin-dependent kinase inhibitors (CKIs) during eukaryotic cell cycle [29], which ensures chromosome replication, uniform segregation of chromosomes to the opposite poles, followed by cytokinesis into two daughter cells. Interestingly, *Plasmodium* cell division involves multiple rounds of nuclear divisions prior to cytokinesis, followed by sequestration of each nucleus with organelles and other cellular contents at the parasite plasma membrane, resulting into multiple daughter cells. This likely requires unique mode of regulation, including a role of the UPS.

More than half of the *P*. *falciparum* proteome represents possible targets of ubiquitination, suggesting both regulatory and homeostatic roles of the UPS [32]. The studies on the role of UPS in *Plasmodium* biology are limited to a few UPS components. The *P*. *falciparum* ubiquitin-activating enzyme 1 (PfUBA1) and ubiquitin C-terminal hydrolase 37 (PfUCH37) have been shown to be essential for erythrocytic stage development [33,34]. The HECT E3 ligase has been shown to have roles in cell cycle progression, invasion of erythrocytes by merozoites, and virulence [35,36]. The knock-out of ubiquitin-conjugating enzyme 13 (UBC13) decreased parasite growth and increased susceptibility to DNA mutagens and dihydroartemisinin [37]. The *P*. *falciparum* ubiquitin C-terminal hydrolase 3 (PfUCH3) has been shown to have dual-hydrolase activity for ubiquitin and neuronal precursor cell-expressed developmentally down-regulated protein 8 (NEDD8) conjugates [38,39]. An endoplasmic reticulum-associated protein degradation (ERAD)-like complex, including the ubiquitination enzymes, has been shown to be localized to the apicoplast, a plastid remnant, and is required for protein transport to the organelle [40,41]. Although *Plasmodium* does not appear to have APC/C, the homologs of human APC/C subunits CDC20/CDH1 and APC3 have been shown to be vital for male gametogenesis [42,43]. In a recent study, Rashpa, *et*. *al*. proposed an SCF-like complex in the mouse malaria parasite *P*. *berghei*, and showed that FBXO1 is required for cell division, gamete egress, and integrity of the inner membrane complex in *P*. *berghei* [44].

We have previously shown that the *P*. *falciparum* cullin homologs are conjugated to NEDD8, which marks the active state of a CRL, and thus suggests active CRLs in *P*. *falciparum* [45]. However, it remains to be investigated if *Plasmodium* cullins form functional complexes, and what are the core components and roles of *Plasmodium* CRLs during parasite development. Given the regulatory roles and possibility of exploitation of ongoing CRL-targeted drug discovery efforts, we investigated *Plasmodium* cullins. We report the core components of *P*. *falciparum* Cullin-1 and Cullin-2 complexes, and demonstrate that these two form functional SCF-like (PfSCF) and CRL-4-like (PfCRL4) ubiquitin E3 ligases, respectively. We further show that PfCRL4 is critical for cell division and membrane integrity.

## Results

### *Plasmodium* genomes code for putative cullin, Rbx1 and Skp1 homologs

The cullin family proteins are conserved in eukaryotes and function as scaffold proteins in CRLs, which form the largest group of ubiquitin E3 ligases. Although conserved across eukaryotes, the number of cullin genes is variable, with eight in *Homo sapiens* and three in *Saccharomyces cerevisiae* [4]. All *Plasmodium* species contain two putative cullin homologs, and we have previously reported that *Plasmodium falciparum* cullins are substrates of neddylation [45,46]. The *P*. *falciparum* Cullin-1 (PfCullin-1, PF3D7_0811000) and Cullin-2 (PfCullin-2, PF3D7_0629800) share 13.9%-24.9% sequence identity with *H*. *sapiens* and *S*. *cerevisiae* cullins. Based on sequence identity with *H*. *sapiens* and *S*. *cerevisiae* cullins, PfCullin-1 is marginally closer to human Cullin-1 (HsCullin-1)/*S*. *cerevisiae* Cullin-1 (ScCullin-1) and PfCullin-2 is marginally closer to human Cullin-4B (HsCullin-4B)/*S*. *cerevisiae* Cullin-8 (ScCullin-8).

We also searched for PfCullin-1 and PfCullin-2 homologs in related apicomplexan parasites, and compared apicomplexan cullins with human cullins. All the apicomplexans sequenced to date have 2–3 cullins, which share 13.3%-66.5% sequence identity with PfCullins, and also share the typical cullin domain architecture, consisting of the signature cullin homology domain and neddylation motif (S1A Fig and S1 Table). Apicomplexan cullins share maximum identity with HsCullin-1, HsCullin-3, and HsCullin-4A or HsCullin-4B (S1 Table). Phylogenetic analysis showed clustering of apicomplexan cullins into three separate groups: PfCullin-1 group, PfCullin-2 group, and the 3^rd^ group that contained the 3^rd^ cullin homolog present in the Sarcocystidae family. *Cryptosporidium parvum* cullin domain containing protein (CDCP) also clustered with the 3^rd^ group. This suggested that PfCullin-1 and PfCullin-2 homologs are conserved across apicomplexa (S1B Fig). PfCullin-1 and PfCullin-2 occupied separate branches on the phylogenetic tree and do not share node with any of the cullins of model organisms, indicating that *Plasmodium* cullins are distantly related to those of the model organisms (S1C Fig).

Cullin-1 associates with several other proteins, including Rbx1 and Skp1, to form the SCF E3 ubiquitin ligase. BLAST search of the reference apicomplexan parasite genomes using *H*. *sapiens* and *S*. *cerevisiae* Rbx1 and Skp1 amino acid sequences identified homologs in all the parasites sequenced to date (S1 Table). *P*. *falciparum* Rbx1 (PfRbx1, PF3D7_0319100) shares 64.7% identity with *H*. *sapiens* Rbx1 (HsRbx1) and 56.6% with *S*. *cerevisiae* Rbx1 (ScRbx1). *P*. *falciparum* Skp1 (PfSkp1, PF3D7_1367000) shares 44.7% sequence identity with *H*. *sapiens* Skp1 (HsSkp1) and 51.9% sequence identity with *S*. *cerevisiae* Skp1 (ScSkp1). Apicomplexan Rbx1 proteins share 21.6%-98.4% sequence identity and contain the characteristic RING_Ubox superfamily (RING_Ubox SF) domain (S2A Fig). Apicomplexan Skp1 proteins share 44.1%-97.1% sequence identity and contain the characteristic Skp1 superfamily (SS) domain (S2B Fig). The presence of cullin, Rbx1 and Skp1 homologs in apicomplexan parasites suggests for the presence of at least two CRL ubiquitin E3 ligases.

### PfCullin-1 and PfCullin-2 are expressed in asexual erythrocytic stages, localize to both cytoplasm and nucleus, and associated with chromatin

**Fig 1:**
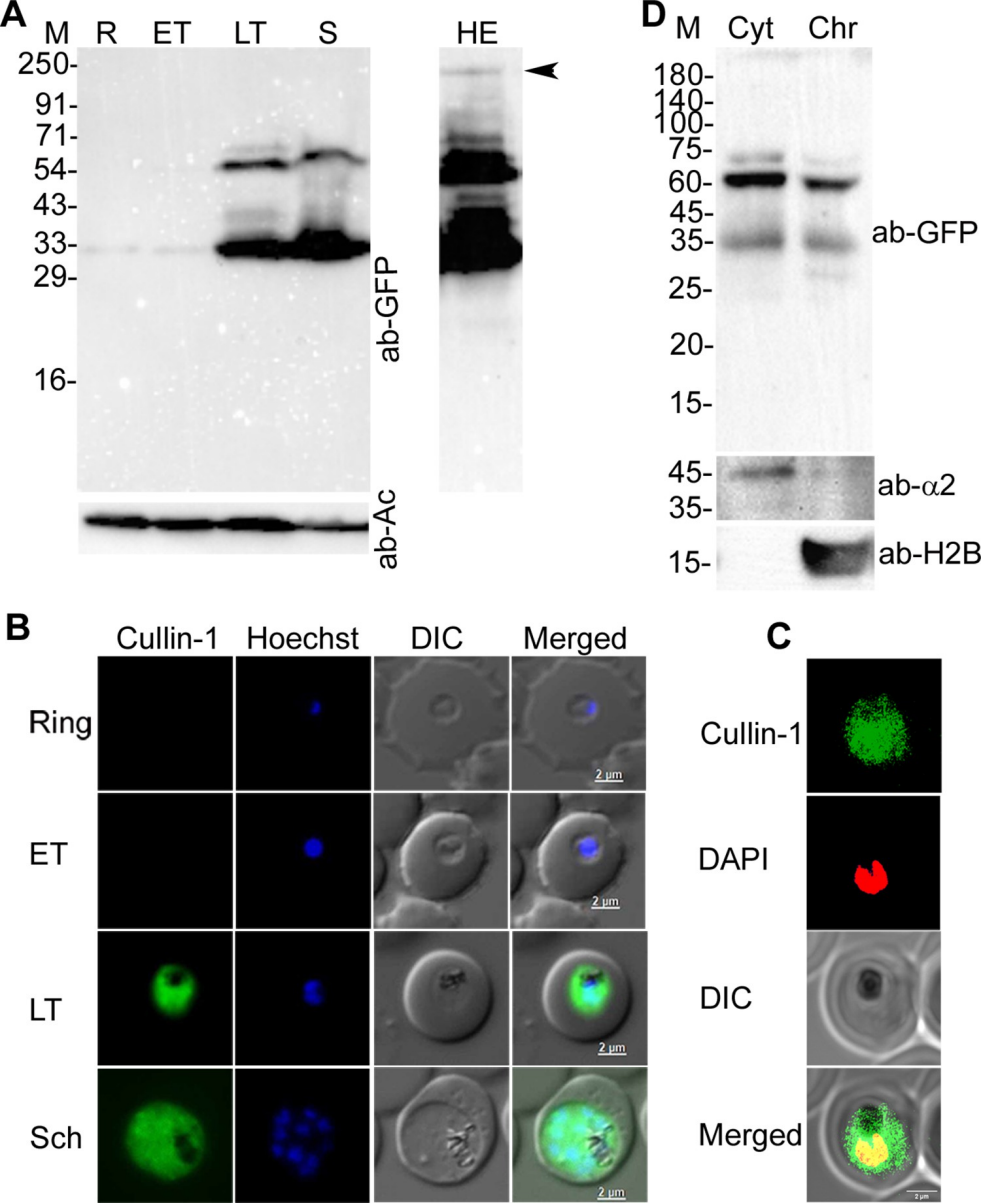
Expression and localization of PfCullin-1. **A.** The lysates of PfCul1GFPKI parasites corresponding to ring (R), early trophozoite (ET), late trophozoite (LT) and schizont (S) stages were assessed for expression of PfCullin-1/GFP and β-actin as a loading control by western blot using anti-GFP (ab-GFP) and mouse anti-β-actin-HRP (ab-Ac) antibodies, respectively. The full-length PfCullin-1/GFP protein (~128 kDa) is indicated by arrow head in the higher exposure panel (HE). **B.** The ring, early trophozoite (ET), late trophozoite (LT) and schizont (Sch) stages of PfCul1GFPKI parasites were assessed for localization of PfCullin-1/GFP by live-cell fluorescence microscopy. The panels show signal for PfCullin-1/GFP (Cullin-1), nuclear stain (Hoechst), bright field with RBC and parasite boundaries (DIC) and overlap of the three images (Merged). **C.** The confocal images of fixed PfCul1GFPKI trophozoite stage show colocalization of PfCullin-1/GFP with the nuclear stain DAPI (Pearson’s coefficient 0.59±0.10 from 27 images). The panels are as in B except that DAPI was used for nuclear staining. **D.** The cytoplasmic (Cyt) and chromatin (Chr) fractions of PfCul1GFPKI trophozoites were assessed for the presence of PfCullin-1/GFP (ab-GFP), cytoplasmic protein α2 proteasome subunit (ab-α2), and nuclear protein histone-2B (ab-H2B) by western blotting. The sizes of protein markers (M) are in kDa and the size scale bars are in the merged panel.

**Fig 2:**
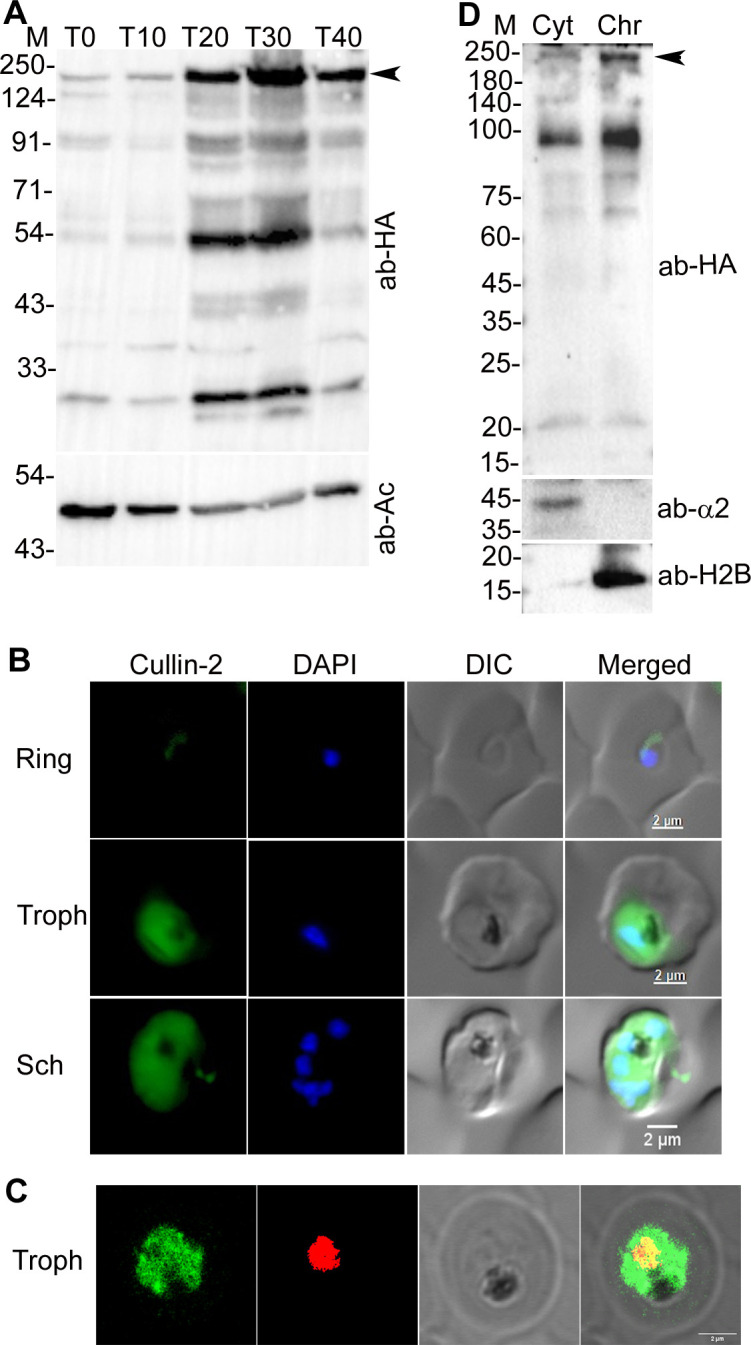
Expression and localization of PfCullin-2. **A.** Lysates of PfCul2KD_D10_ parasites corresponding to early ring (T0), late ring (T10), early trophozoite (T20), late trophozoite (T30) and schizont (T40) stages were assessed for expression of PfCullin-2/cDD_HA_ and β-actin as a loading control by western blot using anti-HA (ab-HA) and anti-β-actin (ab-Ac) antibodies, respectively. The full-length PfCullin-2/cDD_HA_ protein (~155.74 kDa) is indicated with arrow. **B.** The ring, trophozoite (Troph), and schizont (Sch) stages of PfCul2KD_D10_ parasites were assessed for localization of PfCullin-2/cDD_HA_ by IFA using anti-HA antibodies. The panels show signal for PfCullin-2/cDD_HA_ (Cullin-2), nuclear stain (DAPI), bright field with RBC and parasite boundaries (DIC) and overlap of the three images (Merged). **C.** The confocal images of PfCul2KD_D10_ trophozoite (Troph) show colocalization of PfCullin-2/cDD_HA_ with DAPI (Pearson’s coefficient 0.59±0.11 from 13 images). **D.** The cytoplasmic (Cyt) and chromatin (Chr) fractions of PfCul2KD_D10_ trophozoites were assessed for the presence of PfCullin-2/cDD_HA_ (ab-HA), cytoplasmic protein α2 proteasome subunit (ab-α2), and chromatin protein histone-2B (ab-H2B) by western blotting. The sizes of protein markers (M) are in kDa and the size scale bars are in the merged panel.

To study the expression and localization of PfCullins, we replaced wild type PfCullin-1 and PfCullin-2 coding regions with PfCullin-1/GFP and PfCullin-2/cDD_HA_ coding sequences in *P*. *falciparum* D10 strain, respectively. Cloned PfCullin-1/GFP knock-in (PfCul1GFPKI) and PfCullin-2/cDD_HA_ knock-in (PfCul2KD_D10_) parasites were evaluated for replacement of the target gene by PCR and expression of the fusion protein by western blotting. PfCul1GFPKI and PfCul2KD_D10_ parasites showed replacement of the respective wild type coding region with PfCullin-1/GFP and PfCullin-2/cDD_HA_ coding sequences, respectively (S3 Fig). PfCullin-1/GFP was predominantly expressed in late trophozoite and schizont stages (Fig 1A), which is similar to its reported stage-specific transcription profile [47,48]. PfCullin-2/cDD_HA_ is a fusion of PfCullin-2 with HA-tagged mutant *E*. *coli* DHFR, and it was predominantly expressed in trophozoite and schizont stages compared to the ring stage (Fig 2A), which is in agreement with its reported transcription profile [47,48]. The western blot of PfCul1GFPKI parasites had smaller size PfCullin-1/GFP species, and the full-length protein (~128 kDa) was observed at higher exposure only (Fig 1A). The western blot of PfCul2KD_D10_ parasites also had truncated species in addition to the full-length PfCullin-2/cDD_HA_ (~155.7 kDa) (Fig 2A). We did not see any signal in the western blot of wild type parasite lysates with the antibodies used for detection of PfCullin-1/GFP and PfCullin-2/cDD_HA_ (S3C and S3F Fig), indicating that the smaller size bands could be processed forms of full-length proteins. Both PfCullin-1/GFP and PfCullin-2/cDD_HA_ were localized all over the parasite except the black pigment-containing region, which is hemozoin in the food vacuole, a lysosome-equivalent organelle of *Plasmodium* (Figs 1B and 2B). Confocal fluorescence images showed colocalization of PfCullin-1/GFP and PfCullin-2/cDD_HA_ with the nuclear stain DAPI (Figs 1C and 2C), indicating that both these proteins are present in the nucleus as well. As some nuclear CRLs are chromatin-associated and regulate DNA-associated processes [49,50], we checked PfCullin-1/GFP and PfCullin-2/cDD_HA_ for chromatin association. Both the proteins were present in cytoplasmic and chromatin fractions along with the marker proteins (H2B for nuclear fraction and the α2 proteasome subunit for cytoplasmic fraction) (Figs 1D and 2D), suggesting that these proteins have DNA-associated functions.

### PfRbx1 and PfSkp1 localize to both cytoplasm and nucleus, and are associated with chromatin

**Fig 3:**
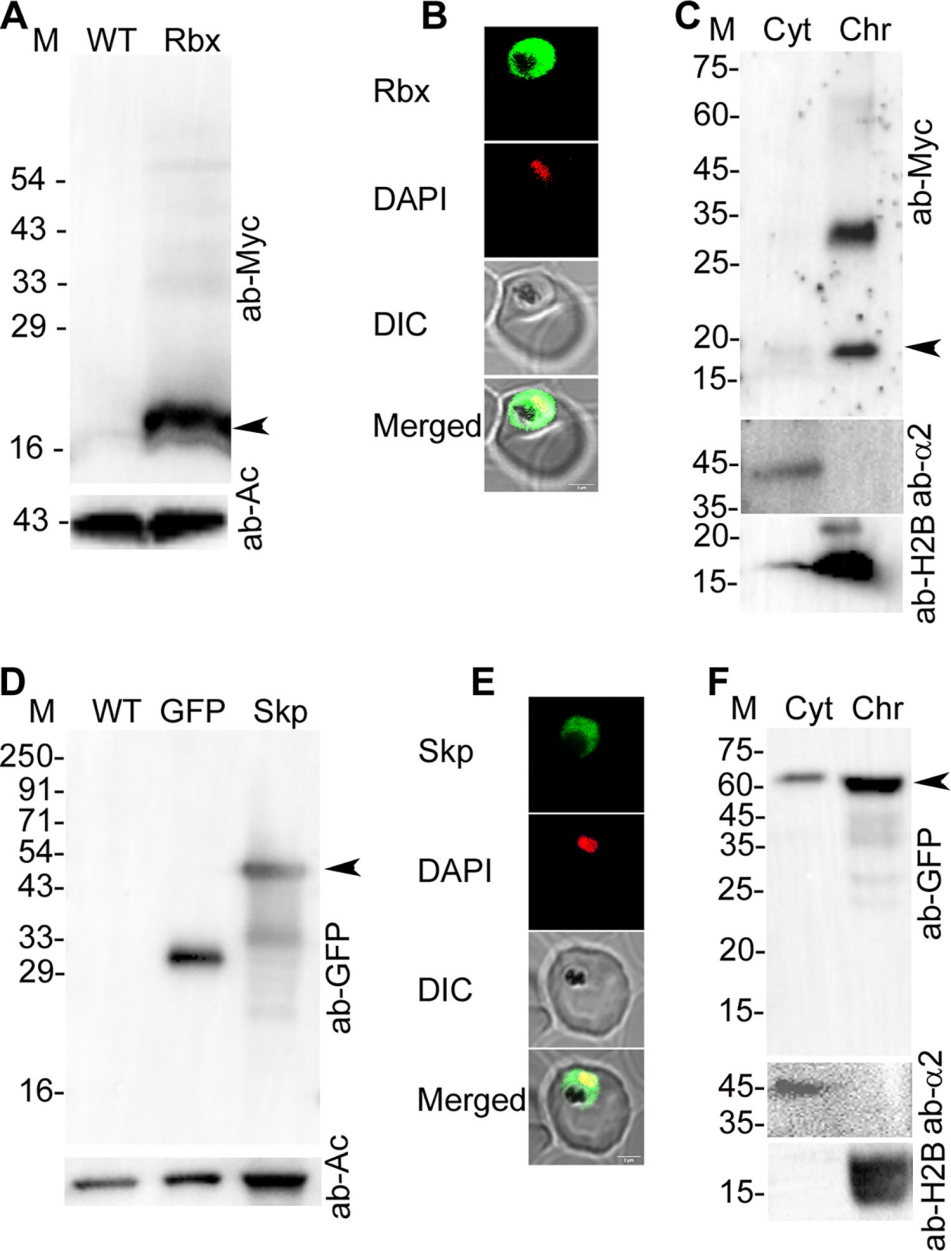
Localization of PfRbx1 and PfSkp1. **A.** The western blot of wild type (WT) and PfRbx1myc-epi (Rbx) parasite lysates shows expression of PfRbx1myc (ab-Myc) and β-actin as a loading control (ab-Ac). The arrow indicates PfRbx1myc protein (~15 kDa). **B.** The confocal images of PfRbx1myc-epi trophozoite show colocalization of PfRbx1myc with the nuclear stain DAPI (Pearson’s coefficient 0.73±0.11 from 20 images). The panels show signal for PfRbx1myc (Rbx), DAPI, bright field with RBC and parasite boundaries (DIC) and overlap of the three images (Merged). **C.** The cytoplasmic (Cyt) and chromatin (Chr) fractions of PfRbx1myc-epi trophozoites were assessed for the presence of PfRbx1myc (ab-Myc), cytoplasmic protein α2 proteasome subunit (ab-α2) and nuclear protein histone-2B (ab-H2B) by western blotting. The arrow indicates PfRbx1myc protein (~15 kDa). **D.** The western blot of wild type (WT), GFP control (GFP) and PfSkp1GFP-epi (Skp) parasite lysates shows expression of PfSkp1GFP or GFP (ab-GFP) and β-actin as a loading control (ab-Ac). The arrow indicates PfSkp1GFP protein (~48 kDa). **E.** The confocal images of fixed PfSkp1GFP-epi trophozoite show colocalization of PfSkp1GFP with the nuclear stain DAPI. The panels show signal for PfSkp1GFP (Skp), DAPI, bright field with RBC and parasite boundaries (DIC), and overlap of the three images (Merged). **F.** The western blot of cytoplasmic (Cyt) and chromatin (Chr) fractions of PfSkp1GFP-epi trophozoites indicates the presence of PfSkp1GFP (ab-GFP), cytoplasmic protein α2 proteasome subunit (ab-α2), and nuclear protein histone-2B (ab-H2B). The arrow indicates PfSkp1GFP protein (~48 kDa). The protein size markers (M) are in kDa, and the size scale bars are shown in the merged image panels.

The coding regions of PfRbx1 with C-terminal Myc-tag (PfRbx1myc) and PfSkp1 with C-terminal GFP-tag (PfSkp1GFP) were episomally expressed in *P*. *falciparum* D10 strain, and the recombinant parasites (PfRbx1myc-epi and PfSkp1GFP-epi) were assessed for localization of tagged-proteins. Western blots of PfRbx1myc-epi and PfSkp1GFP-epi parasites showed expression of the respective fusion proteins (Fig 3A and 3D), which were localized throughout the parasite except the food vacuole and colocalized with the nuclear stain DAPI (Pearson’s coefficient: 0.73±0.11 for PfRbx1myc, 0.55±0.12 for PfSkp1GFP) (Fig 3B and 3E), indicating that PfRbx1 and PfSkp1 are localized to the cytoplasm and nucleus. PfRbx1myc and PfSkp1GFP proteins were also observed in the chromatin fraction along with the control proteins (H2B for nuclear fraction and the α2 proteasome subunit for cytoplasmic fraction) (Fig 3C and 3F). In addition to the expected PfRbx1myc band, a prominent higher molecular weight band was also observed in the chromatin fraction of PfRbx1, which may be a covalently modified form of PfRbx1. The signal intensity of PfRbx1 and PfSkp1 in the cytoplasmic fraction was less than that in the chromatin fraction, which could be due to association of a large fraction of these proteins with protein complexes that are not soluble in the extraction buffer used for isolation of cytoplasmic fraction. On the other hand, the chromatin fraction was obtained by solubilizing the purified chromatin fraction in SDS-PAGE sample buffer, which would extract most of the proteins by breaking the majority of interactions. The nucleocytoplasmic localization and chromatin association of PfCullin-1, PfCullin-2, PfRbx1 and PSkp1 suggest their roles in both the compartments and DNA-associated processes.

### PfCullin-1 forms an SCF-like complex

Since cullin-1, Rbx1 and Skp1 together with other proteins form SCF E3 ubiquitin ligase in several model organisms, we asked if *Plasmodium* has an SCF-like complex. We immunoprecipitated PfCullin-1/GFP, PfRbx1myc, PfSkp1GFP from PfCul1GFPKI, PfRbx1myc-epi and PfSkp1GFP-epi parasites, respectively, using Myc-trap (for PfRbx1myc-epi) or GFP-trap (for PfCul1GFPKI and PfSkp1GFP-epi) antibodies (S4 Fig). As a control, wild type *P*. *falciparum* D10 and GFP-expressing parasites (GFP control) were also processed for immunoprecipitation using Myc-trap and GFP-trap antibodies, respectively (S4 Fig). The immunoprecipitates (IP) were subjected to LC-MS/MS and the proteins present were analysed.

Twenty-five proteins were reproducibly present in PfCullin-1/GFP immunoprecipitates from two of the three biological repeats (S2 Table), including PfCullin-1 and PfRbx1. PfNEDD8 was present in one of the repeats; conjugation of NEDD8 to the cullin subunit of CRLs promotes ubiquitination activity, including the SCF. Rbx1 binds to the C-terminal of cullin, and recruits the E2-ubiquitin conjugate. The presence of PfNEDD8 in PfCullin-1/GFP immunoprecipitates corroborates our previous report of the presence of PfCullin-1 in PfNEDD8 immunoprecipitate, and substantiates neddylation of PfCullin-1 [6]. DDI1 and Histone H2B variant, which are relevant to DNA-associated processes, were also present in the PfCullin-1/GFP immunoprecipitates.

Forty-two proteins were present in PfRbx1myc immunoprecipitates from two of the three biological repeats (S3 Table). Some of the top hits included PfRbx1, PfCullin-1 and PfNEDD8, which are well known components of an SCF. Tubulin gamma chain was also a hit, which is required for microtubule nucleation and is a component of the microtubule organizing centre (MTOC) and substrate of human CRL4 [7]. PfAtg18, cell cycle proteins (nucleosome assembly proteins, actin related protein, regulator of chromosome condensation), spliceosome components, chromatin re-modellers, and proteins involved in chromosome condensation were also present in the PfRbx1myc immunoprecipitates.

Eighteen proteins were reproducibly present in the PfSkp1GFP immunoprecipitates from two of the three biological repeats (S4 Table), including PfSkp1, PfCullin-1, a putative FBXO1 protein (PfFBXO1), and calcyclin binding protein (PfCacyBP), which are also the known components of human SCF, suggesting that these proteins form an SCF complex in *P*. *falciparum*. Polyubiquitin (pUB), DDI1 that is involved in DNA-protein crosslink repair in *P*. *falciparum* [8], DNA repair and recombination protein RAD54, exported protein PHISTb that is involved in host cell remodelling, and anti-oxidant protein DJ1 were some other proteins present in the PfSkp1GFP immunoprecipitates.

**Fig 4:**
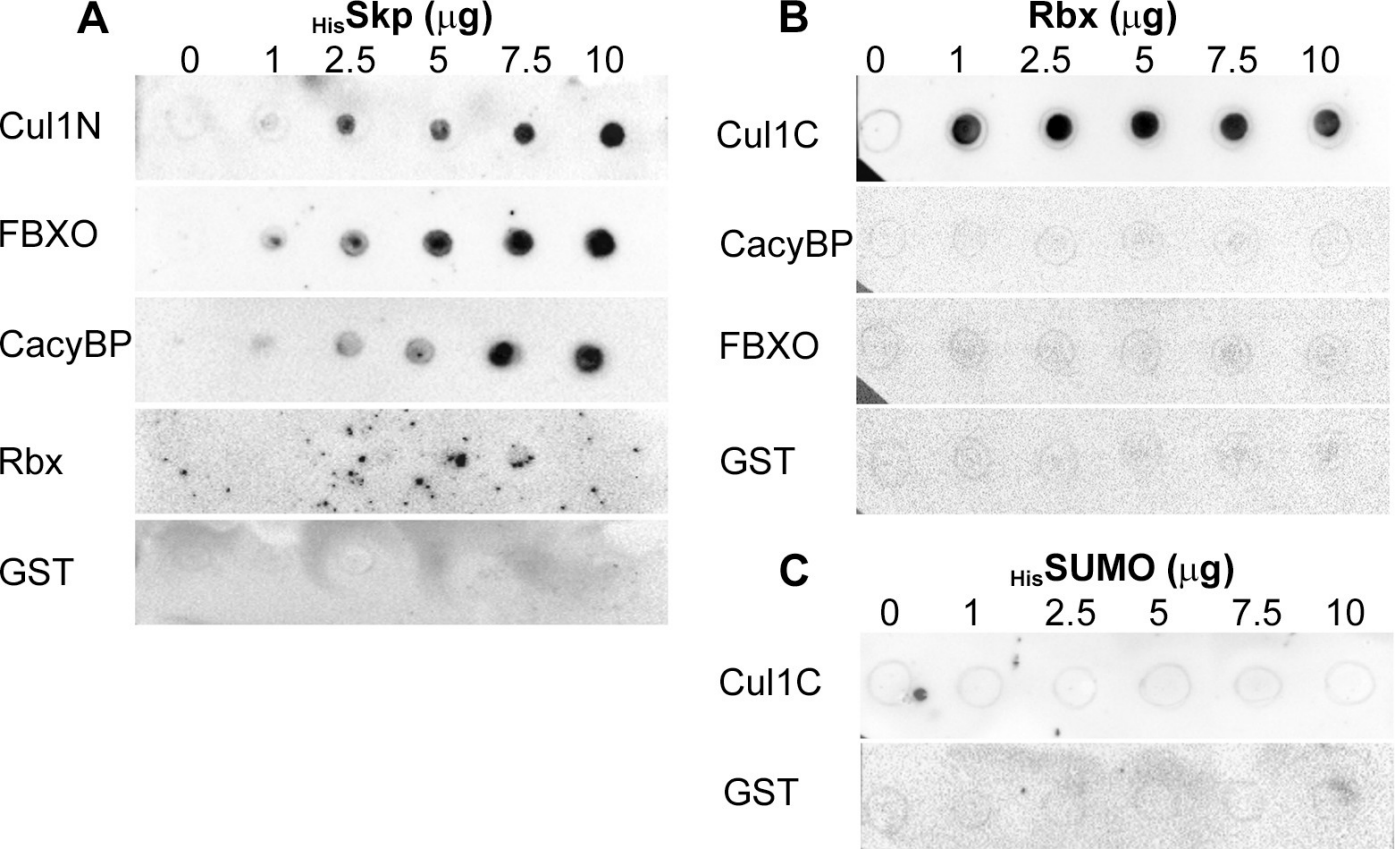
Interactions among PfSCF subunits. **A.** Blots containing spots of the indicated amounts of recombinant _His_Skp were overlaid with recombinant GST/Cul1N (Cul1N), GST/FBXO1 (FBXO), GST/CacyBP (CacyBP), GST/Rbx (Rbx) and GST proteins. The blots were probed with anti-GST antibodies, which revealed interaction of _His_Skp with Cul1N, FBXO, and CacyBP proteins. **B.** The blots containing spots of the indicated amounts of recombinant _His_SUMO/Rbx (Rbx) were overlaid with recombinant GST/Cul1C (Cul1C), GST/CacyBP (CacyBP), GST/FBXO1 (FBXO), and GST proteins. The blots were probed with anti-GST antibodies, which revealed interaction of Rbx with Cul1C. **C.** The blots containing spots of the indicated amounts of recombinant 6×His/SUMO (_His_SUMO) were overlaid with recombinant GST/Cul1C (Cul1C) and GST proteins, and probed with anti-GST antibodies. The blots indicated that _His_SUMO and GST do not interact.

The presence of PfCullin-1, PfNEDD8, PfRbx1, PfSkp1, PfFBXO1 and PfCacyBP in the immunoprecipitates suggested the presence of a typical SCF-like complex in *P*. *falciparum*. In human SCF, cullin-1 binds with Skp1 via its N-terminal and with Rbx1 via its C-terminal. Skp1 associates with FBXO1 through its C-terminal, which then binds to the substrate to be ubiquitinated. Rbx1 recruits an E2-ubiquitin conjugate, and NEDD8 conjugation to cullin-1 promotes transfer of ubiquitin to the substrate [9]. To check whether these proteins directly interact with each other, we produced GST-tagged recombinant N- and C-terminals of PfCullin-1 (GST/Cul1N, 1–300 aa; GST/Cul1C, 601–829 aa), PfFBXO1 (GST/FBXO), PfCacyBP (GST/CacyBP), PfRbx1 (GST/Rbx), and only GST using a bacterial expression system. We also produced 6×His/SUMO-tagged PfRbx1 (_His_SUMO/Rbx), 6×His/SUMO only (_His_SUMO), and 6×His-tagged PfSkp1 (_His_Skp) using a bacterial expression system (S5 Fig). The recombinant proteins were assessed for interaction by dot blot protein overlay assay. _His_Skp interacted with GST/Cul1N, GST/FBXO and GST/CacyBP, but not with GST/Rbx and GST (Fig 4), indicating that the interaction is specific. _His_SUMO/Rbx interacted with GST/Cul1C, but not with GST/CacyBP, GST/FBXO and GST (Fig 4). _His_SUMO alone did not interact with GST/Cul1C and GST, indicating that the interaction of _His_SUMO/Rbx with GST/Cul1C is specific. Thus, our immunoprecipitation and protein overlay data for the first time demonstrate that *P*. *falciparum* contains an SCF-like complex of PfCullin-1, PfSkp1, PfRbx1, PfFBXO1 and PfCacyBP. Furthermore, the presence of PfNEDD8 in the immunoprecipitates of PfCullin-1/GFP and PfRbx1myc, and our previous report of neddylation of PfCullin-1 indicate the PfSCF undergoes neddylation.

### PfCullin-2 appears to form a CRL4B-like complex

**Fig 5:**
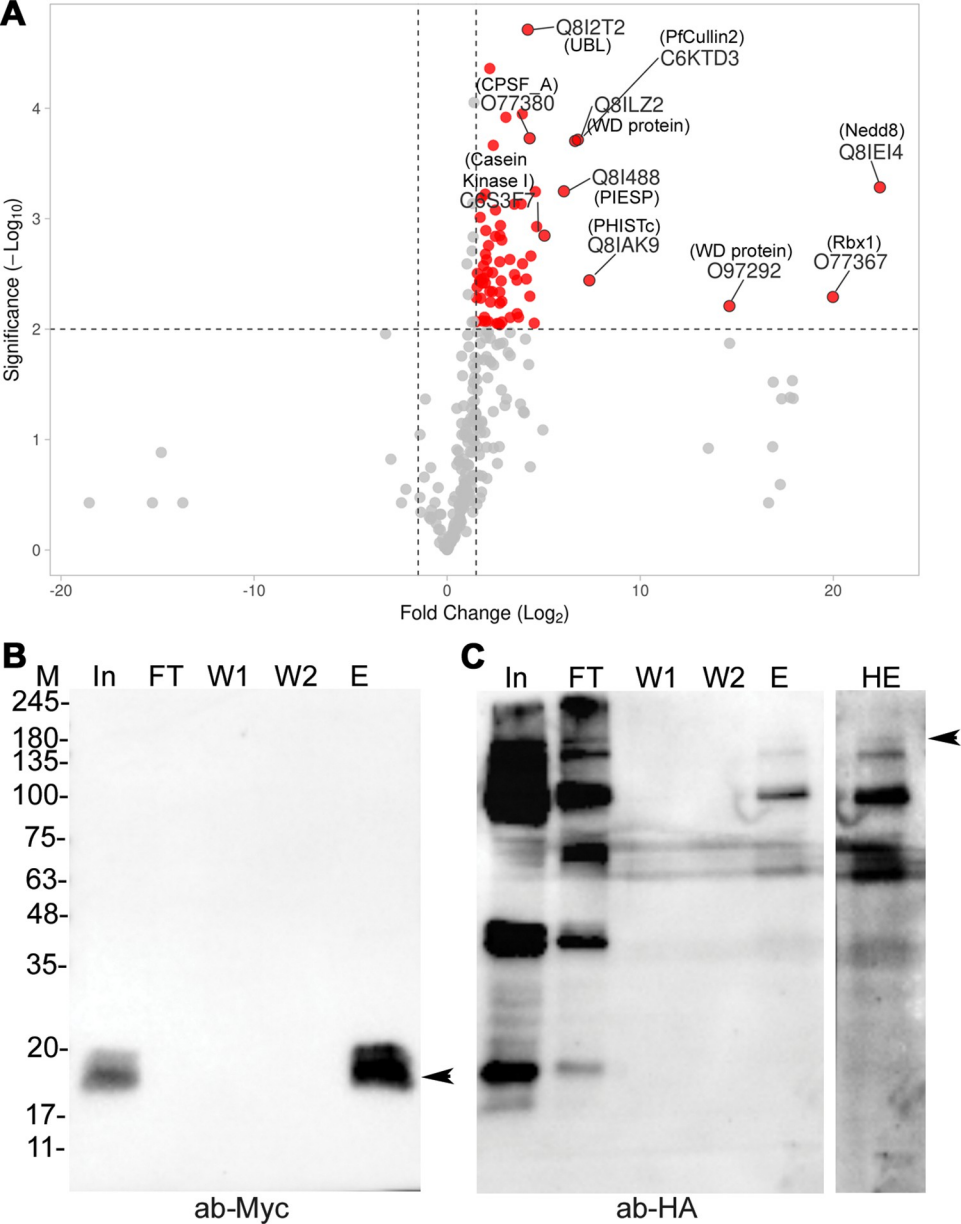
Analysis of the immunoprecipitated proteins and co-immunoprecipitation of PfCullin-2 with PfRbx1. **A.** The plot shows Log2-fold-enrichment (X-axis) of the proteins identified in PfCullin-2 immunoprecipitates (S6A Fig) over those in the control immunoprecipitates (S6B Fig) with significant -Log10 *P*-value (Y-axis). The top 10 hits are labelled. **B.** Equal amounts of PfRbx1myc-epi and PfCul2KD_D10_ parasite lysates were mixed and processed for immunoprecipitation using Myc-Trap antibodies. Aliquots of the mixed lysate (In), flow through (FT), washes (W1, W2) and eluate (E) samples were evaluated for the presence of Rbx1myc by western blotting using anti-Myc antibodies (ab-Myc). The arrow indicates PfRbx1myc protein (~15 kDa). **C.** The blot in B was stripped and probed for PfCullin-2/cDD_HA_ using anti-HA antibodies (ab-HA). The arrow indicates full-length PfCullin-2/cDD_HA_ protein (~155 kDa) at higher exposure (HE). The sizes of protein markers (M) are in kDa.

As cullin is an essential core component of CRLs, we investigated whether PfCullin-2 forms a CRL. We immunoprecipitated PfCullin-2/cDD_HA_ from PfCul2KD_D10_ parasites and subjected the immunoprecipitates to mass spectrometry (S6 Fig). Forty-nine proteins were present in two of the three biological repeats (S5 Table). Among the top 10 hits that are relevant to CRLs were PfCullin-2, cleavage and polyadenylation specificity factor subunit A (PfCPSF_A), two WD40 repeat proteins (PfWD40A and PfWD40B), PfNEDD8, and PfRbx1 (Fig 5A). Sequence analysis of these hits indicated similarity in domain architecture with human Cullin-4B E3 ubiquitin ligase subunits, which includes Cullin-4B as a scaffold protein, DNA damage-specific binding protein 1 (DDB1) as an adaptor protein, Rbx1 as a ubiquitin-E2 recruiter, NEDD8 that promotes the ligase activity, and several WD40 repeat proteins that function as substrate receptors [51]. DDB1 binds to the N-terminal of Cullin-4B and recruits the substrate recognition subunit DDB1-CUL4-associated factor (DCAF), which is a WD40 repeat-containing FBXO protein. Rbx1 binds to the C-terminal of Cullin-4B.

PfCullin-2 shares maximum sequence identity with human Cullin-4B (HsCullin-4B) among all human cullins. Like HsCullin-4B, PfCullin-2 has a long N-terminus upstream of the putative cullin homology domain (703 aa in PfCullin-2 and 216 aa in HsCullin-4B) and a putative nuclear localization signal (S7 Fig), suggesting that PfCullin-2 could be the *Plasmodium* counterpart of HsCullin-4B. A BLAST search of the Uniprot database with PfCPSF_A amino acid sequence identified DDB1 proteins of *A*. *thaliana* (2e-15), *Xenopus laevis* (5e-15), *Rattus norvegicus* (5e-14), *Homo sapiens* (2e-13) and *Mus musculus* (2e-13) as the top five hits. PfCPSF_A also shares domain architecture with DDB1 proteins, suggesting that it could be the *P*. *falciparum* DDB1 homolog (S7 Fig). A BLAST search of the PlasmoDB with human DCAF1 did not show up any homolog. Human DCAF1, PfWD40A and PfWD40B share WD40 domain, and WD40 repeat proteins have been shown to function as substrate receptors for human CRL4 and some E3 ubiquitin ligases [51,52]. It is likely that PfWD40A and PfWD40B serve as substrate receptors. Consistent with our previous report of the presence of PfCullin-2 in PfNEDD8 immunoprecipitates, the presence of PfNEDD8 in PfCullin-2/cDD_HA_ immunoprecipitates indicates neddylation of PfCullin-2 [45].

Attempts to express PfCPSF_A, PfWD40A, PfWD40B and PfCullin-2 in *E*. *coli* were unsuccessful, and we could not assess their direct interaction with each other *in vitro*. Nonetheless, successful co-immunoprecipitation of PfCullin-2/cDD_HA_ with PfRbx1myc from the mixed lysate of PfCul2KD_D10_ and PfRbx1myc-epi parasites strengthens PfRbx1-PfCullin-2 interaction (Fig 5B). The presence of PfCPSF_A, WD40 repeat proteins, PfNEDD8 and PfRbx1 as top hits in the PfCullin-2/cDD_HA_ immunoprecipitates, co-IP of PfCullin-2 with PfRbx1, similarity of PfCPSF_A with HsDDB1 and that of PfCullin-2 with HsCullin-4B together indicate that PfCullin-2 forms a human Cullin-4B-like ubiquitin ligase, which we have termed the *P*. *falciparum* CRL4 (PfCRL4).

PfCullin-2/cDD_HA_ immunoprecipitates also contained several proteins associated with diverse pathways, including phosphorylation (casein kinase 1), virulence (parasite-infected erythrocyte surface protein 2, KAHRP, translocon component PTEX88), exported proteins (exported protein 1 and PHISTc), transport to nucleus (exportin-7 and karyopherin alpha), and RBC invasion by merozoites (rhoptry neck protein 3 and rhoptry neck protein 5). Gene ontology analysis of the proteins identified in the PfCullin-2 immunoprecipitates also showed enrichment of similar pathways, including ubiquitin-proteasomal degradation, protein translation and silencing, membrane protein targeting, DNA damage recognition and repair, metabolism of RNA and proteins, and virulence (S8 Fig). This implicates PfCRL4 in a variety of pathways, and more work will be required to determine a direct link of PfCRL4 association with these pathways.

### PfSCF and PfCRL4 are functional ubiquitin E3 ligases

**Fig 6:**
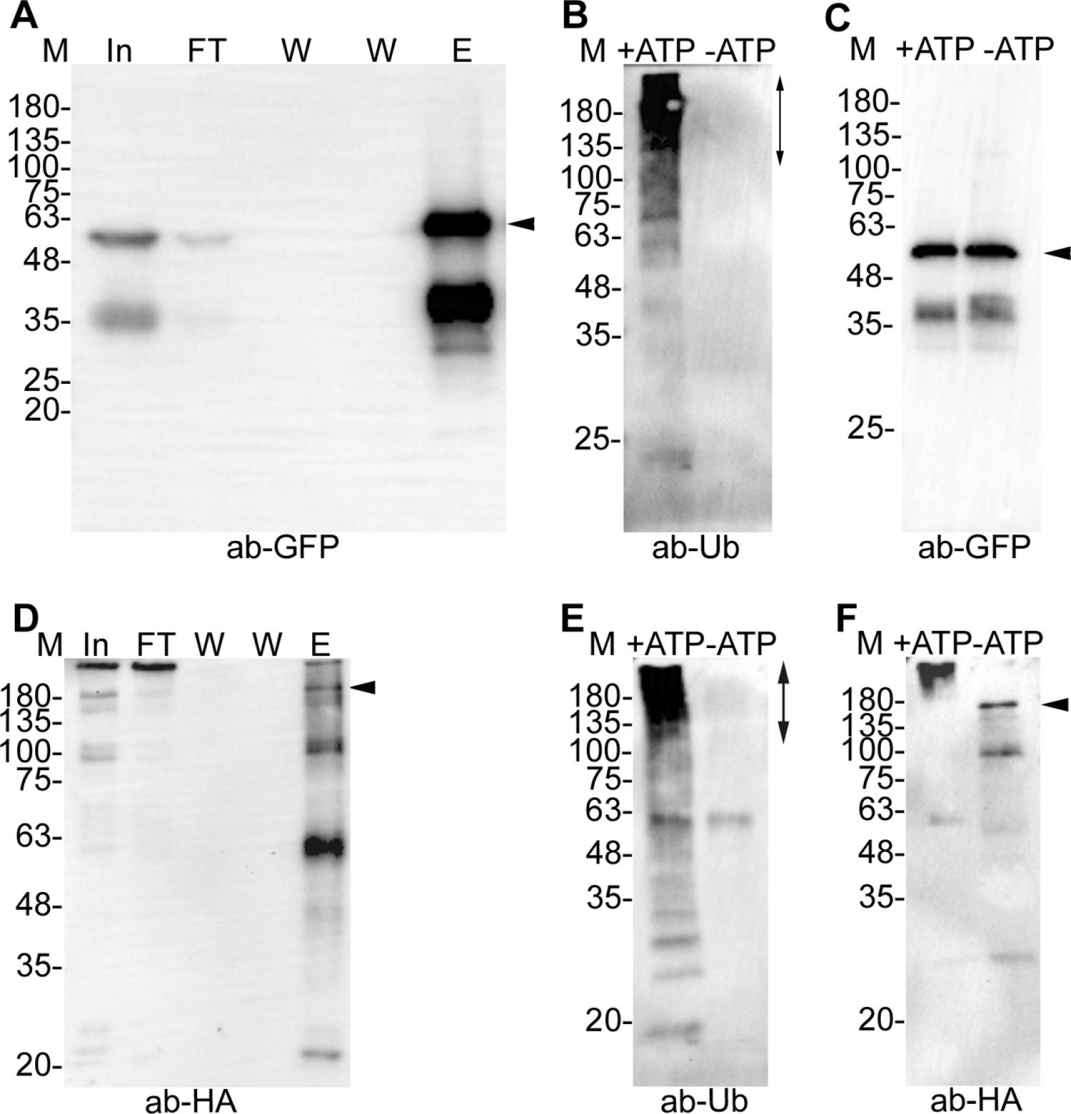
Ubiquitination activity of PfSCF and PfCRL4. PfSkp1GFP-epi and PfCul2KD_D10_ parasite lysates were processed for immunoprecipitation of PfSkp1GFP and PfCullin-2/cDD_HA_ using GFP-Trap and anti-HA antibodies, respectively. The bead-bound immunoprecipitates were washed, eluted or added to ubiquitination reaction with (+ATP) or without (-ATP) ATP as has been described in the methods section. **A.** Aliquots of the parasite lysate input (In), flow through (FT), washes (W), and eluate (E) were evaluated for Skp1GFP by western blotting using anti-GFP antibodies (ab-GFP). **B.** The western blot of PfSkp1GFP-containing ubiquitination reactions with (+ATP) or without (-ATP) ATP was probed with anti-ubiquitin antibodies (ab-Ub). The vertical arrow indicates predominant ubiquitination. **C.** The blot in B was stripped and re-probed with anti-GFP antibodies (ab-GFP) to show that the reactions contained similar amount of PfSkp1GFP. **D.** As in A, the samples of PfCullin-2/cDD_HA_ immunoprecipitation were evaluated for PfCullin-2/cDD_HA_ by western blotting using anti-HA antibodies (ab-HA). **E.** The western blot of PfCullin-2/cDD_HA_-containing ubiquitination reactions with (+ATP) or without (-ATP) ATP was probed with anti-ubiquitin antibodies (ab-Ub). The vertical arrow indicates predominant ubiquitination. **F.** The blot in E was stripped and probed with anti-HA antibodies (anti-HA). The full-length PfCullin-2/cDD_HA_ in +ATP lane migrated at higher size than that in–ATP lane and corresponds to the size of top most ubiquitination signal in +ATP lane, suggesting auto-ubiquitination of PfCullin-2. The sizes of protein markers (M) are in kDa, and arrows indicate the respective proteins in the blots.

We next asked whether PfSCF and PfCRL4 are functional E3 ubiquitin ligases. Although *in vitro* assembled PfSCF and PfCRL4 complexes would have been ideal for ubiquitination activity, attempts to obtain full-length recombinant PfCullin-1, PfCullin-2 and other subunits were unsuccessful due to their large sizes. We used bead-bound PfSkp1GFP immunoprecipitate, which contained most of the PfSCF components (PfCullin-1, PfSkp1, PfFBXO1 and PfCacyBP) except PfRbx1, along with recombinant GST/Rbx, ubiquitin-activating enzyme E1 and ubiquitin-conjugating enzyme E2 for ubiquitination assay in the presence or absence of ATP. The immunoprecipitate showed ATP-dependent ubiquitination activity (Fig 6A–6C), indicating that PfSCF is a functional ubiquitin E3 ligase. Similarly, the bead-bound PfCullin-2/cDD_HA_ immunoprecipitate, which contained all the core components (PfCullin-2, PfCPSF_A, PfRbx1 and WD40 repeat proteins), showed ATP-dependent ubiquitination activity (Fig 6D–6F), indicating that PfCRL4 is a functional ubiquitin E3 ligase. Interestingly, PfCullin-2 appeared to undergo auto-ubiquitination, which could be a regulatory mechanism of PfCRL4 function. Of note, a putative ubiquitin carboxy-terminal hydrolase was consistently co-purified with PfCullin-2/cDD_HA_, which may modulate the activity of PfCRL4.

### PfCRL4 is crucial for asexual and sexual erythrocytic stage development

PfCullin-2 expression in asexual erythrocytic stages suggested a role for it during asexual stage parasite development. Hence, we generated a conditional PfCullin-2 knock-down line in *P*. *falciparum* 3D7 strain by replacing the wild type PfCullin-2 gene with PfCullin-2 coding sequence fused to HA-tagged mutant *E*. *coli* DHFR coding sequence (cDD_HA_). cDD or cDD-fusion protein is stable when bound to trimethoprim (TMP), but undergoes proteasomal degradation in the absence of TMP, allowing inducible knock-down at the protein level [53]. Accordingly, these parasites will have stable PfCullin-2/cDD_HA_ protein in the presence of trimethoprim (+TMP), which would be depleted in the absence of trimethoprim (-TMP). As cullin protein is the scaffold for CRL assembly, depletion of PfCullin-2 would prevent the formation of PfCRL4 complex and would indicate the effect of PfCRL4 depletion on parasite development.

**Fig 7:**
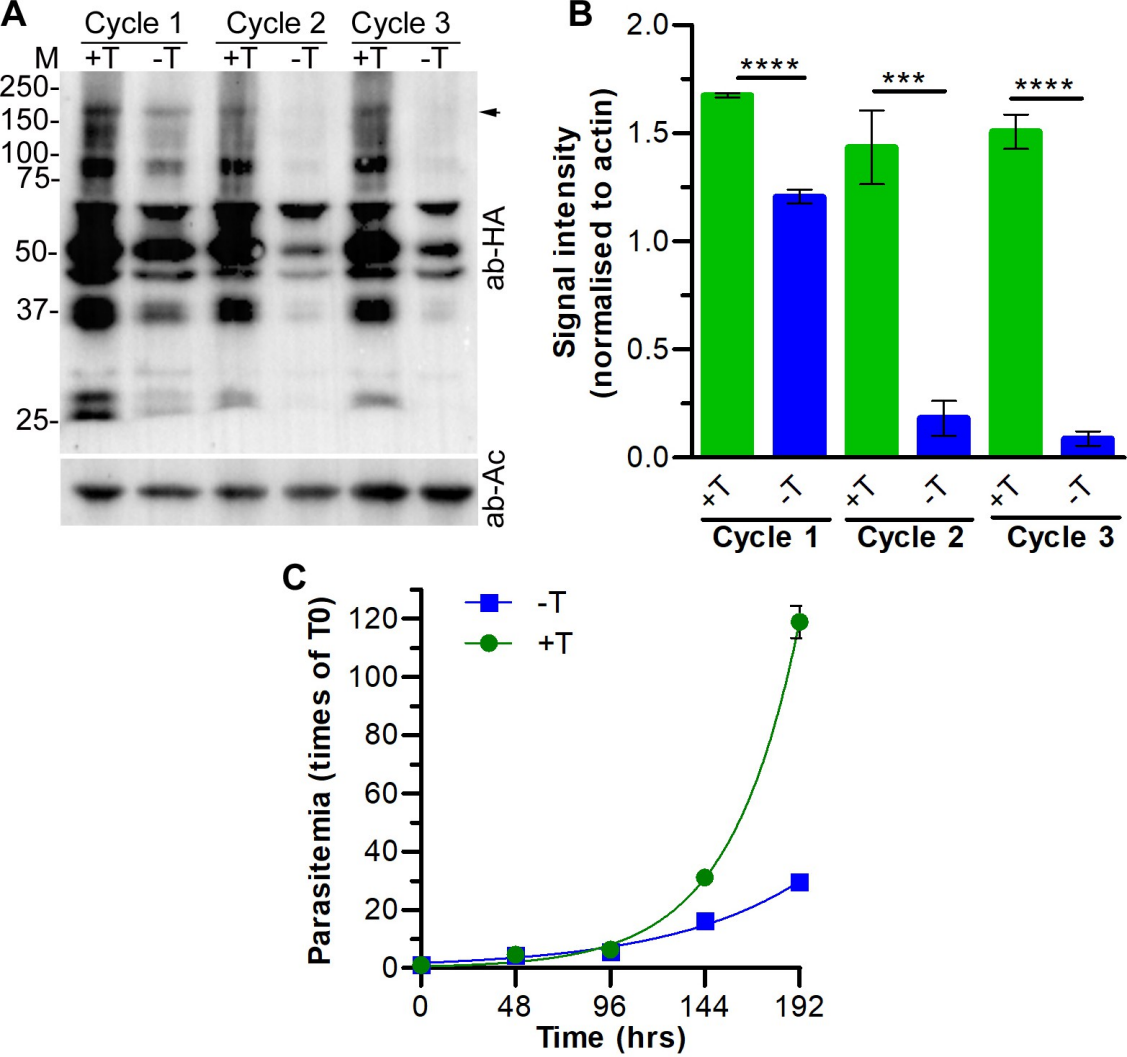
Effect of PfCullin-2 knock-down on asexual erythrocytic stage development. **A.** Synchronized PfCull2KD_3D7_ parasites were cultured with (+T) or without (-T) trimethoprim for three consecutive cycles, each spanning approximately 48 hrs. Equal aliquots of the cultures at the trophozoite stage were harvested in each cycle, and parasite lysates were assessed for PfCullin-2/cDD_HA_ protein level by western blotting using anti-HA (ab-HA) and anti-β-actin (ab-AC) antibodies. The arrow indicates full-length PfCullin-2/cDD_HA_ and the sizes of protein markers (M) are in kDa. **B.** The signal intensity of full-length PfCullin-2/cDD_HA_ and β-actin bands in A were measured, and the signal intensity of full-length PfCullin-2/cDD_HA_ was normalised with the signal intensity of corresponding β-actin band. The plot shows signal intensity of full-length PfCullin-2/cDD_HA_ band on Y-axis for the indicated parasite lysates on X-axis. The data is mean of three independent experiments with SD error bar. **C.** Synchronized PfCull2KD_3D7_ parasites were cultured with (+T) or without (-T) trimethoprim for four consecutive cycles. Parasitemia was determined at the beginning and end of each cycle, and shown as fold multiplication of the starting parasitemia over time. The data is mean of three independent experiments with SD error bar.

**Fig 8:**
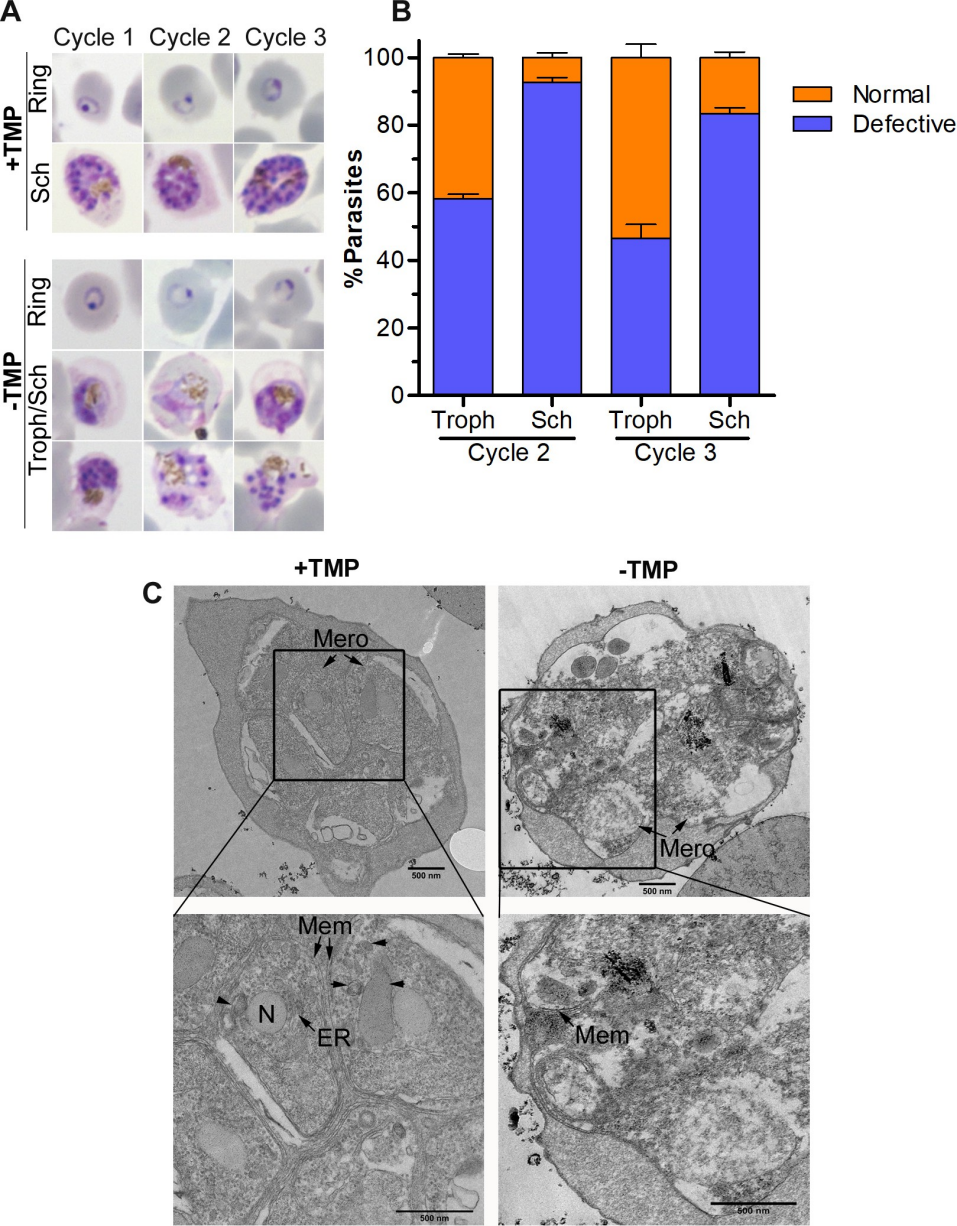
Effect of PfCullin-2 knock-down on parasite morphology. Synchronized PfCull2KD_3D7_ parasites were cultured with (+TMP) or without (-TMP) trimethoprim for three consecutive cycles, and parasites were observed for morphology at the end of each cycle. **A.** Shown are the Giemsa-stained smears of PfCull2KD_3D7_ parasite cultures from three cycles (Cycle 1–3). **B.** The plot shows percentage of normal and morphologically defective (fragmented cell, indistinct nuclei and scattered hemozoin) trophozoites (Troph) and schizonts (Sch) in cycle 2 and cycle 3. The data is from two experiments, each based on at least 100 trophozoites/schizonts observed. **C.** Shown are the TEM images of 3^rd^ cycle schizonts with multiple merozoites (Mero). The zoomed-in section shows clearly delimited membraned structures (N: nucleus, ER: endoplasmic reticulum, Mem: membrane) in +TMP parasites, which are poorly visible in -TMP parasites.

PCR and western blot of a cloned recombinant line (PfCul2KD_3D7_) confirmed the replacement of wild type PfCullin-2 with PfCullin-2/cDD_HA_ coding sequence and expression of PfCullin-2/cDD_HA_ protein, respectively (S9A and S9B Fig). IFA showed PfCullin-2/cDD_HA_ expression in asexual stages of PfCul2KD_3D7_ parasites (S9C Fig). PfCul2KD_3D7_ parasites cultured in the absence of TMP showed decreased PfCullin-2/cDD_HA_ protein level and significantly lower parasitemia than those grown in the presence of TMP (Fig 7A–7C). The knock-down effect was partial in the 1^st^ cycle and became prominent in 2^nd^ and 3^rd^ cycles. Hence, we primarily focussed on 2^nd^ and 3^rd^ cycle parasites for further evaluation of the effect of PfCullin-2 depletion on parasite development. Giemsa smears of PfCul2KD_3D7_ parasites cultured without TMP, but not those cultured with TMP, revealed clear morphological defects (distorted membrane, scattered hemozoin and shrunken nuclei) in a large number of trophozoites/schizonts (Fig 8A and 8B). Transmission electron micrographs of PfCul2KD_3D7_ parasites cultured with TMP showed clear demarcation of merozoites inside the schizont with distinct outer membrane and intracellular organelles, whereas the schizonts of parasites cultured without TMP showed loss of overall morphology with poor demarcation of the merozoite membrane and intracellular organelles (Fig 8C). Scanning electron microscopy images of PfCul2KD_3D7_ parasites cultured without TMP showed aberrant surface topology in 58% of the cells as compared to only 5.6% of the cells in parasites cultured with TMP (S10 Fig). Together, these data indicate that PfCRL4 is critical for asexual erythrocytic development and membrane integrity.

**Fig 9:**
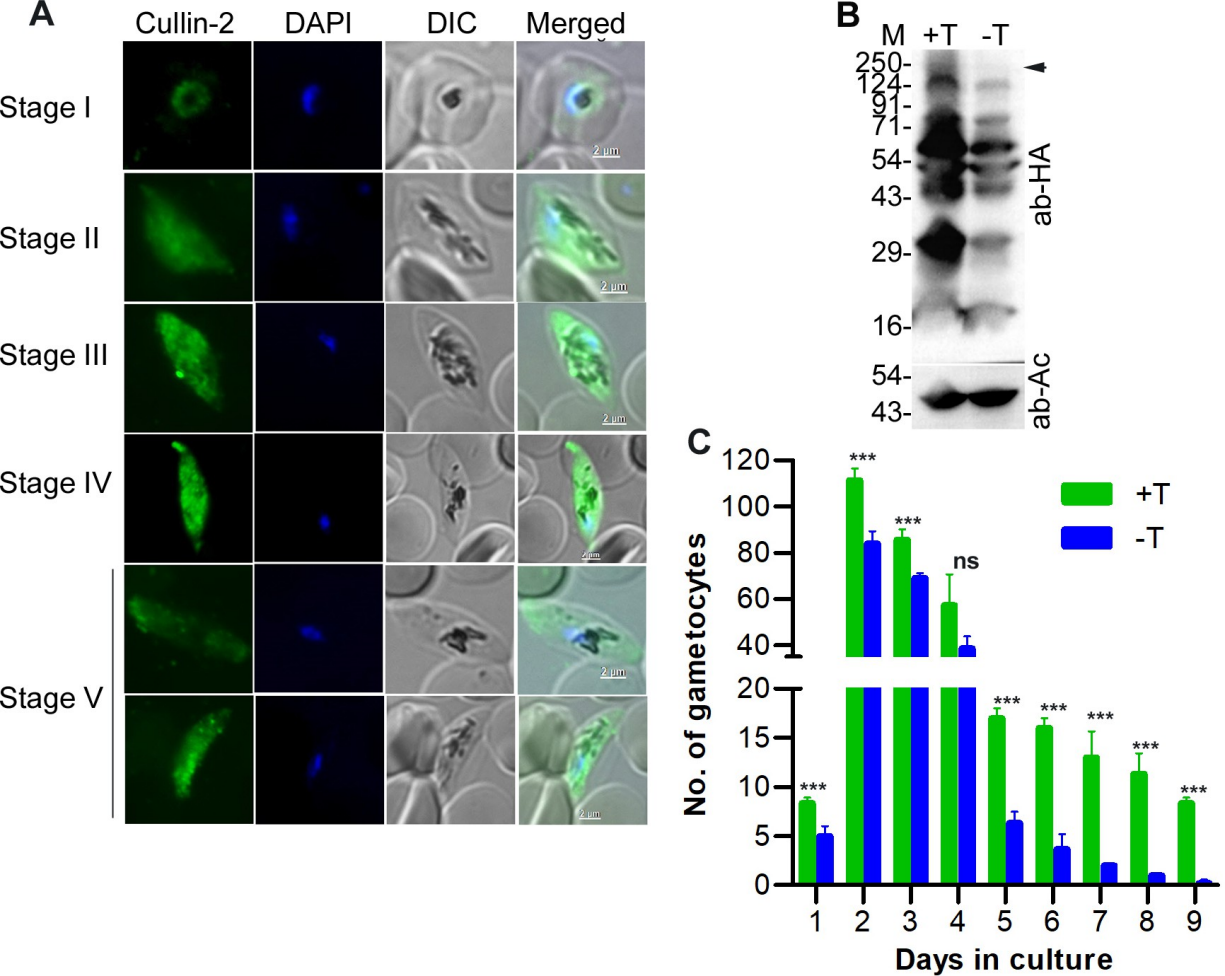
Effect of PfCullin-2 knock-down on sexual erythrocytic stage development. **S**ynchronized PfCull2KD_3D7_ parasite cultures with (+T) or without (-T) trimethoprim were used to induce gametocytogenesis, and treated with NAG to eliminate asexual stages. **A.** The +T culture was evaluated for expression of PfCullin-2/cDD_HA_ in gametocytes by IFA using anti-HA antibodies. The images for the indicated gametocyte stages show PfCullin-2/cDD_HA_ signal (Cullin-2), nuclear staining (DAPI), parasite and erythrocyte boundaries in the infected cell (DIC), and overlap of all the three images (Merged). The size scale bars are shown in the merged image panels. **B.** Parasites were isolated from +T and–T cultures on day-4, and assessed for PfCullin-2/cDD_HA_ protein level by western blotting using anti-HA (ab-HA) and anti-β-actin (ab-AC) antibodies. The full-length PfCullin-2/cDD_HA_ is indicated with arrow, and the sizes of protein markers (M) are in kDa. **C.** The number of gametocytes/2000 cells in +T and -T cultures were estimated, and shown as the number of gametocytes over days in culture. The data is mean of three independent experiments with SD error bar.

The IFA of PfCul2KD_3D7_ gametocytes indicated prominent expression of PfCullin-2/cDD_HA_ in all gametocyte stages (Fig 9A), which prompted us to assess the effect of PfCullin-2 knock-down during sexual erythrocytic stage development. We induced gametocytogenesis and maintained gametocytes with or without TMP in the culture. The western blot of day 4 gametocytes without TMP showed decreased PfCullin-2 protein level as compared to those grown with TMP (Fig 9B), and there was a significant reduction in the number of gametocytes in -TMP culture (Fig 9C), indicating an important role of PfCRL4 during sexual erythrocytic stage development.

### PfCullin-2 knock-down altered the levels of proteins associated with proteasome, lipid biosynthesis and transport, protein translation and folding, DNA repair, and apicoplast

**Fig 10:**
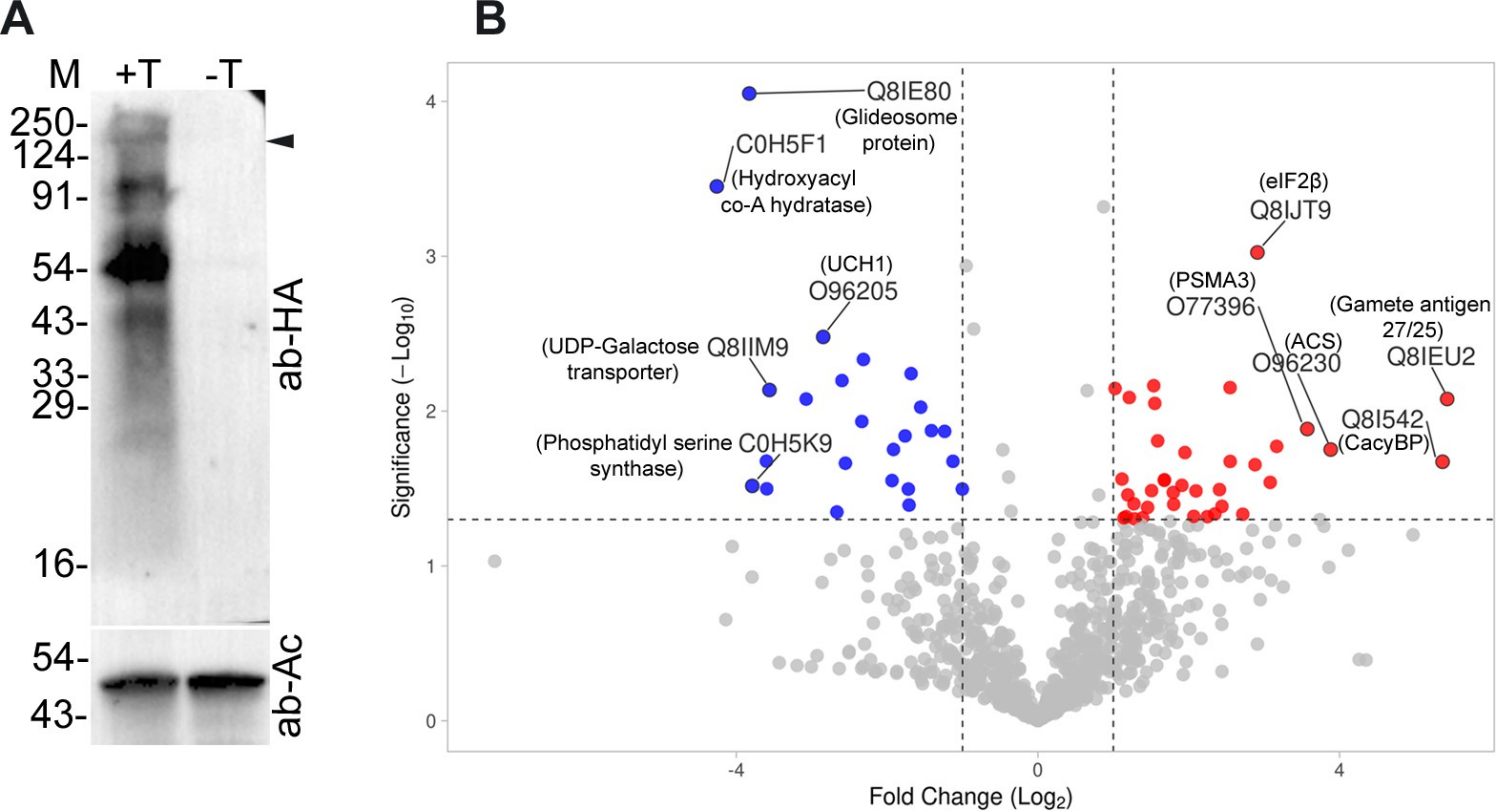
Changes in protein levels upon PfCullin-2 knock-down. Synchronized PfCul2KD_3D7_ parasites were grown with (+T) or without (-T) trimethoprim for three cycles, and the lysates of 3^rd^ cycle trophozoites were compared for PfCullin-2/cDD_HA_ protein level by western blotting and changes in the proteome by LFQ. **A.** The western blot shows PfCullin-2/cDD_HA_ (ab-HA) and β-actin (ab-Ac) protein levels. The full-length PfCullin-2/cDD_HA_ is indicated with arrow, and the sizes of protein markers (M) are in kDa. **B.** The volcano plot shows up- and down-regulated proteins with at least 2-fold change (*P*-value of ≤0.05) in–T parasites. The X-axis shows Log2 fold change and Y-axis shows significance as -Log10 *P*-value (cut off 1.3 = 0.05).

CRLs are involved in ubiquitination-dependent turnover and signalling of a large number of proteins, and gene ontology analysis of the PfCullin-2 interactome suggested association of PfCullin-2 in multiple pathways (S8 Fig). Hence, we quantitated and compared the proteomes of 3^rd^ cycle PfCul2KD_3D7_ trophozoites cultured in the presence and absence of TMP by label free quantification (LFQ) method. PfCullin-2 was absent in the western blot as well as the mass spectrometry data of -TMP parasites (Fig 10A), which confirmed successful knock-down. Compared to the +TMP, -TMP parasite sample had 128 proteins down-regulated (log2 fold change = ≤1), 199 proteins up-regulated (log2 fold change = ≥1) and 557 proteins unchanged (log2 fold change between = ±1) in two biological replicates. Of these up- or down-regulated proteins, 37 proteins were up-regulated and 23 proteins were down-regulated with a *P*-value of ≤0.05 (Figs 10B and S11 and S6 Table). We analysed significantly up- or down-regulated proteins for pathway analysis (S11 Fig). Some of the up-regulated proteins are associated with proteasome and peptidases, transcription/translation and protein folding, ion transport, DNA replication and repair, and metabolism. The majority of down-regulated proteins are associated with lipid biosynthesis and transport, nucleoside/metabolite transport, and ribosome biogenesis. Proliferating cell nuclear antigen 2 (PCNA2) has been shown to be associated with DNA damage in malaria parasites [54], and it was significantly elevated, suggesting DNA damage stress in PfCullin-2-depleted parasites. Seven apicoplast-associated proteins, a major site of fatty acid synthesis, and 8 proteins associated with lipid synthesis and transport were found to be differentially regulated. Significant up- and down-regulation of these proteins could compromise lipid biosynthesis and subsequently membrane integrity, which might be responsible for the disrupted morphology of PfCullin-2-depleted trophozoites/schizonts. Further investigation is required to understand how PfCRL4 is associated with the above pathways.

### PfCullin-2 knock-down increased susceptibility to DNA damage and ER stress, and decreased mitochondrial membrane potential

**Fig 11:**
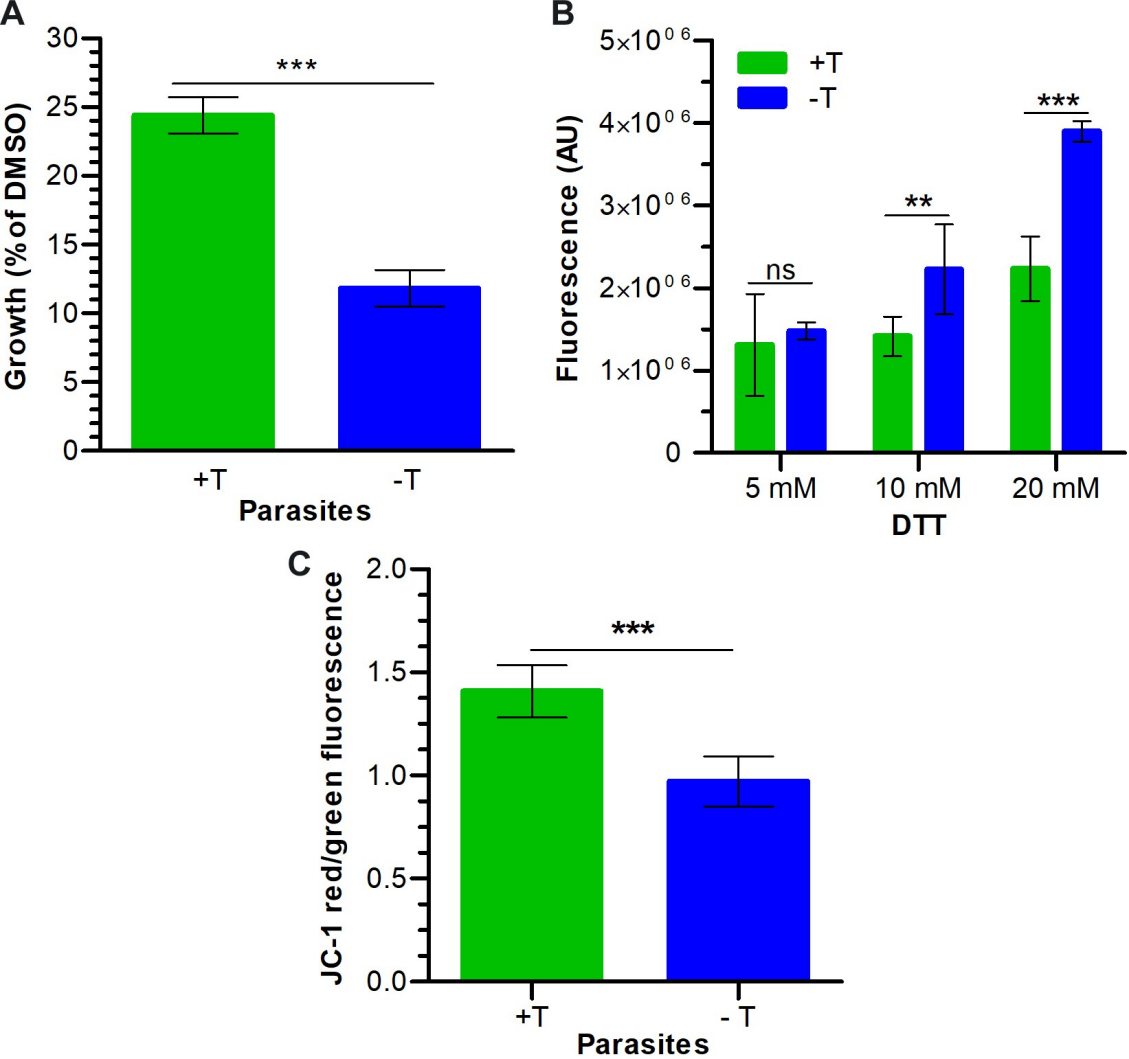
Effect of PfCullin-2 knock-down on susceptibility to stresses. PfCul2KD_3D7_ parasites were grown with (+T) or without (-T) trimethoprim for 3 cycles, and the 3^rd^ cycle trophozoite stage parasites were evaluated for mitochondrial membrane potential, and susceptibility to DNA-damaging agent and ER stress. **A.** The trophozoite stage parasites were treated with DMSO or the DNA-damaging agent MMS for 6 hours, and the number of fresh rings formed 12 hrs after the treatment was counted. The number of fresh rings in each culture is shown as % of its DMSO control for the same. **B.** The trophozoite stage parasites were treated with the indicated concentrations of DTT and ROS production was measured using H_2_DCFDA. The plot shows fluorescence values in arbitrary units (AU) upon DTT treatment. **C.** The trophozoite stage parasites were stained with JC-1, and the JC-1 red and green fluorescence were measured. The plot shows the ratio of JC-1 red/green fluorescence (Y-axis) for the indicated parasites (X-axis). Each data is mean of three independent experiments with SD error bar.

The proliferating cell nuclear antigen 2 (PCNA2) was upregulated upon PfCullin-2-depletion, which is an indicator of DNA damage response [55], and suggested that these parasites might have genotoxic stress-like condition. We evaluated PfCul2KD_3D7_ parasites for susceptibility to the DNA-damaging agent methyl methanesulfonate (MMS), which has been shown to alkylate DNA [56]. PfCul2KD_3D7_ parasites were grown with or without TMP for 3 cycles, the 3^rd^ cycle trophozoite stage parasites were treated with DMSO or MMS, and the number of rings in each case was counted to assess recovery. The MMS-treated -TMP parasites produced almost 50% lesser number of rings than +TMP parasites (Fig 11A), indicating increased susceptibility to DNA-damaging agent upon PfCullin-2-depletion, which is in agreement with upregulation of PCNA2 and association of PfCRL4 with DNA damage recognition and repair pathways.

PfCullin-2-depleted parasites had disrupted morphology and dysregulation of several proteins associated with lipid biosynthesis and transport, proteasome and peptidases, protein translation and folding, which suggested perturbed proteostasis and ER stress. DTT has been shown to induce ER stress in a variety of cells, including *Plasmodium* [11], which results in the production of reactive oxygen species (ROS) that converts non-fluorescent 5-(and-6)-carboxy-2’,7’-dichlorofluorescein diacetate (H_2_DCFDA) into fluorescent DCF, and this increase in fluorescence is directly proportional to ROS. Accordingly, PfCul2KD3_D7_ parasites were grown with or without TMP for 3 cycles, and the 3^rd^ cycle parasites were treated with DTT followed by with H_2_DCFDA. The PfCul2KD3_D7_ parasites grown without TMP showed more fluorescence than those grown with TMP condition, indicating increased ER stress upon PfCullin-2 depletion (Fig 11B).

PfCullin-2-depleted parasites also showed altered levels of three proteins associated with mitochondria, which prompted us to assess these parasites for mitochondrial membrane potential as a measure of mitochondrial activity. For this, PfCul2KD_3D7_ parasites were grown with or without TMP for 3 cycles, the 3^rd^ cycle trophozoite stage parasites were stained with JC-1, and the red/green ratio was determined (Fig 11C). The -TMP parasites showed lower red/green ratio of J-aggregates than +TMP parasites, indicating decreased membrane potential, which is an indicator of increased mitochondrial stress.

### PfCullin-2 knock-down blocked cell division

**Fig 12:**
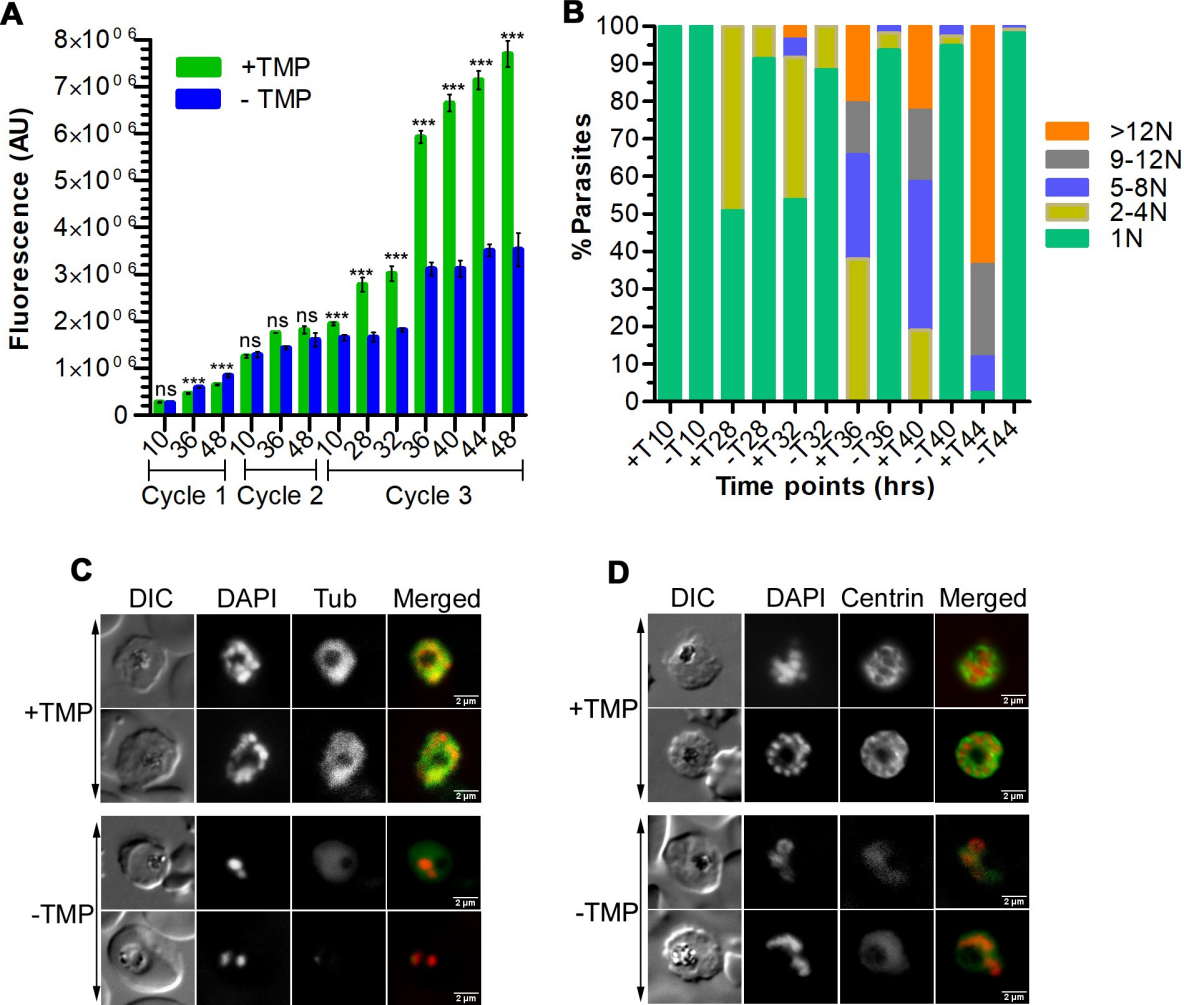
Effect of PfCullin-2 knock-down on cell division. Synchronized PfCul2KD_3D7_ parasites were grown with (+TMP) or without (-TMP) trimethoprim for three cycles, beginning with equal parasitemia. **A.** Equal aliquots of the cultures were collected at different time points over three cycles, stained with SYBR green I, and the fluorescence of each sample was measured. The plot shows fluorescent in arbitrary units (AU) on Y-axis for samples corresponding to the indicated time points of each cycle (X-axis). The data is from a representative of three experiments, each done in triplicate. “*” indicates *P*-value for the significance of difference between +TMP and -TMP data. **B.** The aliquots of 3^rd^ cycle cultures were smeared on glass slides, stained with DAPI, and the number of nuclei/parasite were counted using a microscope. The plot shows percentage of parasites with the indicated number of nuclei/parasite (Y-axis) at the indicated time points (X-axis). The data is based on at least 100 parasites for each time point from three experiments. **C and D.** Trophozoite/schizont stages during the 3^rd^ cycle were processed for IFA using anti-α-tubulin (C) or anti-centrin (D) antibodies. The panels are for the bright field image showing parasite and RBC boundaries (DIC), nuclear stain (DAPI), α-tubulin (Tub) or centrin, and the overlap of all three images (Merged). The size scale bar is in the merged image.

PfCullin-2-depleted parasites showed significantly reduced growth, which prompted us to compare the total DNA content and number of nuclei/parasite in PfCul2KD_3D7_ parasites grown with or without TMP for 3 cycles. The DNA contents of +TMP and -TMP parasites increased as parasites matured and were largely similar until the 3^rd^ cycle ring stage (10 hr time point) (Fig 12A). As expected, the DNA content of +TMP parasites increased with maturation in the 3^rd^ cycle, whereas the DNA content of -TMP parasites was significantly lower than that of +TMP parasites (Fig 12A). Since the total DNA content does not accurately reflect the division state of parasite, we counted the number of nuclei/parasite at different time points during the 3^rd^ cycle. The number of multinucleated cells (2-4N to >12N) increased with the maturation of +TMP parasites, whereas the majority of -TMP parasites had single nucleus (1N) throughout the development, indicating replication arrest (Fig 12B).

To further examine nuclear division, we examined the 3^rd^ cycle trophozoites/schizonts (40 hr time point) of +TMP and -TMP parasites for mitotic structures (spindle and microtubule organizing centre) using antibodies to α-tubulin and centrin-1, which have been used successfully earlier for studying *Plasmodium* cell division in [57–59]. The +TMP parasites showed prominent tubulin- and centrin-labeled structures in association with each nucleus, which were significantly diminished in -TMP parasites (Fig 12C and 12D). In particular, the +TMP parasites showed prominent tubular centrin signal with each nucleus, whereas the -TMP parasites had faint and diffuse centrin signal, suggesting that mitotic structures did not form upon PfCullin-2 depletion. The decreased DNA content, less number of nuclei/parasite and diminished mitotic structures in PfCullin-2-depleted parasites together indicate that PfCRL4 is required for cell division.

## Discussion

Cullin-RING ubiquitin E3 ligases (CRLs) are critical for protein balance, genomic integrity, and cell division fidelity [29–31]. CRLs are associated with several human diseases and are the focus of many ongoing drug discovery efforts [60]. However, the core components and roles of CRLs during the development of *Plasmodium* are not known. In this study, we demonstrate two functional CRLs in the human malaria parasite *P*. *falciparum*: a typical SCF-like (PfSCF) and a human CRL4-like (PfCRL4) ubiquitin E3 ligase. PfSCF comprises of PfCullin-1, PfSkp1, PfRbx1, PfFBXO1 and PfCacyBP. PfCRL4 appears to contain PfCullin-2, PfCPSF_A, two WD40 repeat proteins, and PfRbx1. PfCRL4 is crucial for cell division and membrane integrity, and likely contributes to proper functioning of mitochondria, ER, and genomic maintenance.

We analyzed putative cullin, Skp1 and Rbx1 sequences of representative apicomplexan parasites to identify their closest *Plasmodium* and human counterparts. All the apicomplexan parasites analyzed in this study have 2–3 putative cullins, one Skp1 and one Rbx1, which suggests that there are at least two CRLs, including an SCF-like ubiquitin E3 ligase. We could not annotate apicomplexan cullins as a particular human cullin homolog due to their low sequence identity with human cullins (<35%), which was also reflected as separation of *P*. *falciparum* cullins from those of model organisms on the phylogenetic tree. This suggests parasite-specific adaptation in function and composition of *P*. *falciparum* CRLs to regulate the complex and multi-stage parasite development. This is also supported by the absence of canonical CRL substrates like cyclins and CDKs in *P*. *falciparum* [61].

PfCullin-1 and PfCullin-2 expression was predominant in trophozoite and schizont stages, which corresponds to their reported transcription profiles [47,48]. The nucleocytoplasmic localization of PfCullin1, PfCullin-2, PfSkp1, and PfRbx1 suggests function in both the compartments, which is consistent with the reported localization and function of most of the CRLs in model systems [62].

Mass spectrometry of the immunoprecipitates followed by direct protein-protein interaction confirmed that PfCullin-1, PfSkp1, PfFBXO1, PfCacyBP and PfRbx1 form a SCF-like complex (PfSCF). PfSkp1 and PfRbx1 likely function as substrate adaptor and ubiquitin E2 recruiter, respectively. PfFBXO1 has an F-box motif, suggesting that it is an F-box protein and likely functions as a substrate receptor, as has been reported for F-box proteins [63]. Human CacyBP has been shown to have roles in p53-induced degradation of non-phosphorylated human β-catenin [64], cytoskeleton organization, dephosphorylation of ERK1/2, cell proliferation and cancer development [65]. However, the homologs of p53, β-catenin and ERK1/2 appear to be absent in *Plasmodium*, suggesting that PfCacyBP has *Plasmodium*-specific roles.

PfCullin-2 likely forms a human CRL4B-like complex (PfCRL4), as several top hits (PfCullin-2, PfCPSF_A, PfRbx1 and WD40 repeat proteins) present in the PfCullin-2 immunoprecipitates share domain architecture with human CRL4B core subunits. The human CRL4B core subunits include HsCullin-4B, HsDDB1, HsDCAF1 and HsRbx1, which serve as a scaffold protein, substrate adaptor, substrate receptor and ubiquitin E2 recruiter, respectively [51]. PfCullin-2, like HsCullin-4B, has a long N-terminus upstream of the predicted cullin homology domain (CH) and a putative nuclear localization signal (NLS). A BLAST search of the Uniprot database using PfCPSF_A sequence identified DDB1 proteins, suggesting that it could be an HsDDB1 homolog. HsDDB1 has been proposed to interact with several WD40 repeat proteins, including DCAF1, which have been shown or predicted to function as substrate receptors [66]. Several F-box proteins with WD40 repeats function as substrate receptors [63], and PfWD40 repeat proteins may serve as substrate receptors. Notably, PfCullin-2 immunoprecipitates also contained a putative ubiquitin carboxy-terminal hydrolase, which showed most similarity with ubiquitin carboxy-terminal hydrolase 7 (UCH7) proteins, and we have termed it the *P*. *falciparum* ubiquitin carboxy-terminal hydrolase 7 (PfUCH7). The *H*. *sapiens* ubiquitin specific protease 7 (USP7) has been shown to be involved in a variety of pathways and associated with multiple ubiquitin E3 ligases, including MDM2 and RAD18. This association has been shown to be important for regulation of HsUSP7 and the interacting ligases [67]. PfUCH7 or its *Plasmodium* orthologs have not been characterized yet to our knowledge, and it may regulate PfCRL4 function by performing ubiquitin chain editing and/or deubiquitinating PfCRL4 subunits, which agrees with autoubiquitination of PfCullin-2.

Due to the large sizes of most of the PfSCF and PfCRL4 subunits and AT-richness of their coding genes, we could not produce these subunits as recombinant proteins for assembling the complexes *in vitro* to perform functional assays. Hence, we evaluated PfSkp1 and PfCullin-2 immunoprecipitates for ubiquitination activity. Both the immunoprecipitates showed ATP-dependent ubiquitination activity, indicating that PfSCF and PfCRL4 complexes are functional ubiquitin E3 ligases. PfCullin-2 appeared to undergo autoubiquitination, which may regulate PfCRL4 functions, as autoubiquitination has been shown to regulate the stability and function of several ubiquitin E3 ligase [68]. Since the ubiquitinated proteins are the co-immunoprecipitated proteins, these could be the substrates of PfSCF or PfCRL4. Further investigation is needed to identify specific substrates of PfSCF and PfCRL4, which will provide insights into their specific targets and roles in specific biological pathways. A previous study also identified Cullin-1, Skp1, Rbx1, and FBXO1 in the Skp1-immunoprecipitate of *T*. *gondii*, and proposed an SCF-like CRL in *T*. *gondii* [69]. Rashpa, *et*. *al*. in a recent study performed extensive immunoprecipitation of Rbx1, Skp1 and FBXO1 of the mouse malaria parasite *P*. *berghei*, and proposed that these proteins form an SCF in *P*. *berghei* [44]. While our results for PfSCF are in agreement with these two reports, we extended the composition, confirmed one-one interaction between different components, and demonstrated a functional SCF in the human malaria parasite *P*. *falciparum*. The identification of functional PfCRL4 in our study is the first report for *Plasmodium* to our knowledge.

PfCul2KD_3D7_ parasites showed depletion of PfCullin-2 in the absence of trimethoprim (-TMP), which would prevent the formation of the PfCRL4 complex, thereby allowed us a comparative assessment of -TMP and +TMP parasites. The PfCullin-2-depleted parasites (-TMP) showed significantly decreased growth as compared with the parasites grown in the presence of trimethoprim (+TMP), and the knock-down effect was partial in the 1^st^ cycle and became prominent in the 2^nd^ and 3^rd^ cycles. The partial knock-down effect could be due to essentiality of PfCullin-2 for parasite development, which has also been observed for other essential proteins [70,71]. We speculate that there could be some level of functional redundancy in PfCRL4 functions, which may compensate for it for a short period but not for prolonged parasite development. This is somewhat similar to the delayed death phenomenon observed in *P*. *falciparum* upon treatment with translation inhibitor antibiotics [72]. PfCullin-2 depletion did not change the overall ubiquitination activity, as the whole cell lysates or extracts of +TMP and -TMP parasites showed similar ubiquitination signal (S12 Fig). These results suggest that PfCullin-2 ubiquitinates a selected set of parasite proteins, which warrants for the identification of its substrates. Furthermore, *P*. *falciparum* has several putative ubiquitin E3 ligases [73], which would contribute to the overall ubiquitination activity of the parasite, and could mask contribution of a single ubiquitin E3 ligase to the total ubiquitination activity.

Gene ontology analysis of the PfCullin-2 interactome suggests roles for it in several pathways, which is in-line with up- and down-regulation of proteins belonging to various metabolic pathways in PfCullin-2-depleted parasites. Notably, the levels of 7 apicoplast-associated proteins and 8 proteins associated with lipid synthesis/transport were highly altered in PfCullin-2-depleted parasites. Apicoplast is an essential, non-photosynthetic plastid remnant that provides isoprenoid precursors, fatty acids and heme for parasite development [74–76]. We speculate that significant change in the levels of the apicoplast-associated proteins and the proteins associated with lipid synthesis/transport in PfCullin-2-depleted parasites would adversely affect apicoplast and lipid biosynthesis, thereby affecting the membrane integrity and biogenesis. Inhibition of apicoplast-associated processes has been shown to cause delayed parasite death in subsequent cycles [77], which is similar to the delayed effects of PfCullin-2-depletion in our study. Further investigation is required to identify a direct link between PfCRL4 and apicoplast. A link between lipid metabolism and ER stress has been shown both at cellular and organismal level [78,79], which is in-line with our results. Deletion of CRL4 *in S*. *cerevisiae*, mammalian cells, *C*. *elegans*, and plants has been shown to cause compromised DNA repair and replication [31,51,80], and this is in agreement with our data showing increased susceptibility to DNA-damaging agents upon PfCullin-2 depletion. These data together with gene ontology analysis explain decreased mitochondrial membrane potential, and increased susceptibility to DNA damaging agents and ER stress upon PfCullin-2-depletion.

A large number of PfCullin-2-depleted trophozoites/schizonts were morphologically defective. Compared to +TMP schizonts, the -TMP schizonts had decreased number of merozoites with poorly demarcated outer merozoite membrane and membraned structures, indicating a defect in the formation and segregation of merozoites and suggesting a key role of PfCRL4 in cell division. As has been explained earlier, the morphological abnormalities of PfCullin-2-depleted parasites could be due to impaired lipid biosynthesis. Compared to the +TMP parasites, PfCullin-2-depleted parasites had less total DNA content, fewer number of nuclei/parasite, and diminished mitotic structures, which confirmed a block in cell division. Nuclear localization and chromatin-association of PfCullin-2, the presence of several nuclear proteins in the PfCullin-2 immunoprecipitate, and altered levels of several nuclear proteins upon PfCullin-2 depletion suggest that PfCullin-2 regulates several nuclear processes. Depletion of PfCullin-2 could have adversely affected these processes, causing a block in cell division.

Rashpa, *et*. *al*. recently reported that genetic ablation or depletion of the *P*. *berghei* SCF component FBXO1 blocked cell division during erythrocytic schizogony, prevented gamete egress from the host erythrocyte, and affected integrity of apical and the inner membrane complexes in merozoite and ookinete [44]. Our results with *P*. *falciparum* complement the findings of Rashpa, *et*. *al*., and these two studies together indicate that *Plasmodium* SCF and CRL4 have essential, but non-overlapping, functions during the development of malaria parasites.

**Fig 13:**
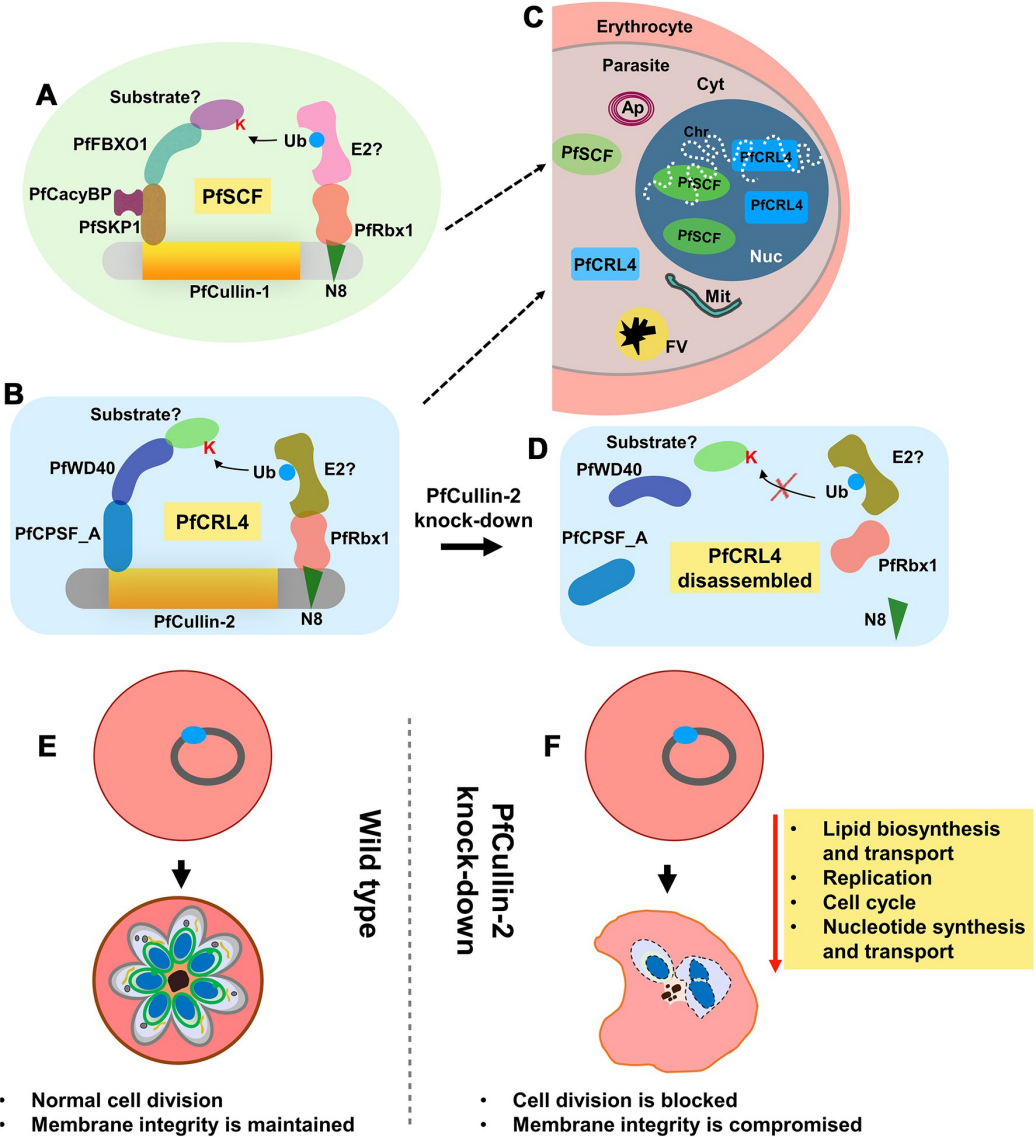
Highlights of the study. PfCullin-1 and PfCullin-2 serve as scaffolds for assembly of the indicated proteins into functional PfSCF (A) and PfCRL4 (B) ubiquitin E3 ligases, respectively. Both the complexes localize (C) to cytoplasm (Cyt) and nucleus (Nuc), and associate with chromatin (Chr), suggesting their roles in these compartments, including DNA-associated processes. Apicoplast (Ap), mitochondria (Mit) and food vacuole (FV) with haemozoin (black structure) are also shown in the parasite within the erythrocyte. PfCullin-2 knock-down at protein level would prevent assembly of the indicated subunits into functional PfCRL4 (D), thereby affecting the associated processes. Wild type asexual erythrocytic parasite developed normally from ring to multinucleate schizonts (E), whereas PfCullin-2 knock-down parasites exhibited down-regulation of several key pathways, resulting in a block in cell division and compromised membrane integrity (F). Contrary to the wild type schizont that showed multiple nuclei with distinct outer membrane and intracellular membraned structures, the PfCullin-2 knock-down schizont had less number of nuclei and poorly demarcated membraned structures.

In summary, our study shows that *P*. *falciparum* Cullin-1 and Cullin-2 form functional PfSCF and PfCRL4 ligases, which likely have roles in the cytoplasmic, nuclear and DNA-associated processes. PfCRL4 is crucial for asexual and sexual erythrocytic development, and has key roles in cell division and maintenance of membrane integrity (Fig 13). Our findings will pave the way for similar studies in other apicomplexan parasites, which is necessary to uncover the biology and drug-target potential of CRLs in parasitic protozoa.

## Materials and methods

### Materials

All the routinely used biochemicals were of molecular biology grade and procured from MERCK or similar sources unless otherwise stated. Cell culture reagents were from Lonza and Thermo Fisher Scientific. Restriction enzymes and DNA modifying enzymes were from New England Biolabs and Thermo Fisher Scientific. DNA isolation kits were from QIAGEN and MACHEREY-NAGEL. ProLong Diamond Antifade Mountant, Hoechst, DAPI, SuperSignal Chemiluminescent substrate and 2’,7’-dichlorodihydrofluorescein diacetate acetyl ester (H_2_DCFDA) were from Thermo Fisher Scientific. Secondary antibodies were from Thermo Fisher Scientific and Jackson ImmunoResearch. Primary antibodies were from Cell Signalling Technologies, Thermo Fisher Scientific, MERCK and ChromoTek. WR99210 was a kind gift from David Jacobus (Jacobus Pharmaceutical, Princeton, U.S.A.). Protease inhibitor cocktail, trimethoprim and N-acetylglucosamine (GlcNAc) were from MERCK. Glutaraldehyde, formaldehyde, osmium tetroxide, lead citrate solution, and sodium cacodylate buffer were from Electron Microscopy Sciences. Uranyl acetate was from Loba Chemie and epoxy resin constituents were from TED PELLA. *P*. *falciparum* 3D7 and D10 strains were obtained from the Malaria Research and Reagent Reference Resource centre (MR4). All the plastic ware was from standard manufacturers such as Corning Inc, Nalgene, Thermo Fisher Scientific and Tarsons.

### Methods

#### Ethics statement

Human blood was collected after obtaining written informed consent from the subjects according to the protocols (IEC-38/2015, IEC-38-R1/2015, IEC-38-R2/2015 and IEC-38-R3/2015) approved by the Institutional Ethics Committee (IEC) of Centre for Cellular and Molecular Biology. All the studies abide by the Declaration of Helsinki principles.

#### Sequence analysis and phylogeny

For identification of cullin homologs in *P*. *falciparum*, the amino acid sequences of *H*. *sapiens* cullins (HsCullin-1 (Q13616), HsCullin-2 (Q13617), HsCullin-3 (Q13618), HsCullin-4A (Q13619), HsCullin-4B (Q13620), HsCullin-5 (Q93034), HsCullin-7 (Q14999), and HsCullin-9 (Q8IWT3)) and *S*. *cerevisiae* cullins (ScCullin-1 (YDL132W), ScCullin-3 (YGR003W), and ScCullin-8 (P47050)) were used as queries in BLAST searches of the *Plasmodium* genome database PlasmoDB (https://plasmodb.org/plasmo/app) and the NCBI non-redundant protein sequence database (https://blast.ncbi.nlm.nih.gov/Blast.cgi). For identification of Rbx1 and Skp1 homologs in *P*. *falciparum*, the amino acid sequences of *H*. *sapiens* Rbx1 (HsRbx1 (P62877)) and Skp1 (HsSkp1 (P63208)), and *S*. *cerevisiae* Rbx1 (ScRbx1 (Q08273)) and Skp1 (ScSkp1 (P52286)) were used as queries in BLAST searches of the *Plasmodium* genome database PlasmoDB. To search for cullin, Rbx1 and Skp1 homologs in apicomplexan parasites, amino acid sequences of *P*. *falciparum* cullin-1 (PfCullin-1), cullin-2 (PfCullin-2), Rbx1 (PfRbx1) and Skp1 (PfSkp1) were used as queries in the BLAST searches of the eukaryotic pathogen genomics database resource EuPathDB (https://veupathdb.org/veupathdb/app). Sequence alignment was performed using Clustal Omega (https://www.ebi.ac.uk/Tools/msa/clustalo/), and amino acid sequences were analysed for conserved domains and motifs using CD-BLAST program (https://www.ncbi.nlm.nih.gov/Structure/cdd/wrpsb.cgi). For phylogeny, amino acid sequences were aligned using the MAFFT program (https://www.ebi.ac.uk/Tools/msa/mafft/), the phylogenetic tree was constructed using IQ-TREE (http://iqtree.cibiv.univie.ac.at/) with 1000 bootstrap alignments [81,82], and the tree was visualized with iTOL (https://itol.embl.de/).

#### Parasite culture

Whole human blood was collected, peripheral blood mononuclear cells were removed by washing the blood with RBC storage medium (RPMI 1640 supplemented with 2 g/l glucose, 25 mg/l gentamicin, 300 mg/l glutamine), and RBCs were stored at 50% haematocrit in RBC storage medium at 4°C until used. *P*. *falciparum* 3D7 and D10 strains were grown at 37°C in a mixed gas environment (5% CO_2_, 5% O_2_ and 90% N_2_) in RPMI 1640-albumax medium with human erythrocytes at 2% haematocrit (RPMI 1640 supplemented with 2 g/l sodium bicarbonate, 2 g/l glucose, 25 mg/ml gentamicin, 300 mg/l glutamine, 100 mM hypoxanthine, 0.5% albumax II) [83]. Parasites were synchronized by sorbitol treatment when the majority of parasites were at ring stage [84], and parasites were isolated from infected RBCs by saponin lysis. Parasite pellets were used immediately in experiments or stored at −80°C until further use. Genomic DNA was isolated from late trophozoite/schizont stage parasites using the Puregene Blood Core kit as recommended by the manufacturer.

#### Construction of transfection plasmids

The sequences of primers used in this study are mentioned in S7 Table. The complete open reading frames of PfRbx1 and PfSkp1 were PCR amplified from *P*. *falciparum* genomic DNA using primers PfRbx-F/PfRbxmyc-R and PfSkp1-F/PfSkp1-R, respectively. PfRbxmyc-R contains Myc-tag coding sequence for addition to the C-terminus of PfRbx1. The PfRbx1myc PCR fragment was cloned into the pGEM-T vector, and subcloned into the pNC plasmid at BglII/XhoI sites to obtain pNC-PfRbx1myc transfection plasmid; pNC has been described earlier [45]. The PfSkp1 PCR fragment was cloned into the pGT-GFP vector at BglII/KpnI sites upstream of the GFP coding sequence [85], excised with BglII/XhoI to obtain PfSkp1/GFP, which was cloned into the similarly digested pNC vector to obtain pNC-PfSkp1/GFP transfection plasmid. PfCullin-1 locus was targeted for single cross over homologous recombination. The PfCullin-1 coding region corresponding to C-terminal 500 amino acid residues was PCR amplified from *P*. *falciparum* 3D7 genomic DNA using PfCul1Fki/PfCul1Rki primers, digested with ApaI/AgeI, and cloned into the similarly digested pNC-GFP vector upstream of the GFP coding sequence to obtain pNC-PfCullin-1/GFPki transfection plasmid. Double cross-over homologous recombination approach was used to target the PfCullin-2 locus. The PfCullin-2 coding sequence corresponding to C-terminal 409 amino acid residues (flank1) and 867 bp 3’-UTR of PfCullin-2 (flank2) were PCR amplified from *P*. *falciparum* 3D7 genomic DNA using PfCullin2F1kiFP/PfCullin2F1kiRP and PfCullin2F2F/PfCullin2F2R primers, respectively. The flank1 and flank2 PCR fragments were sequentially cloned into the HBA18/cDD_HA_kd plasmid at NotI-KpnI and AvrII-KasI sites, respectively, to obtain HB-PfCul2/cDD_HA_ plasmid. The HBA18/cDD_HA_kd plasmid has been described earlier [86]. A synthetic gene coding for the *Bacillus subtilis* glmS ribozyme and PvAc-3’U was obtained from (GenScript), and cloned into the HB-PfCul2/cDD_HA_ plasmid at XhoI/AgeI site, replacing the existing PvAc-3’U fragment to generate HBglm-PfCul2/cDD_HA_ transfection plasmid. The coding regions and flanks in all the transfection plasmids were sequenced to ensure that they were free of undesired mutations, and the presence of different regions was confirmed by digestion with region-specific restriction enzymes. The transfection plasmids were purified using the NucleoBond Xtra Midi plasmid DNA purification kit.

#### Transfection of parasites

Freshly thawed cultures of *P*. *falciparum* D10 and 3D7 strains were used for transfection. *P*. *falciparum* D10 early ring stage parasites were transfected with 50–100 μg of the desired transfection plasmid (pNC-PfRbx1myc, pNC-PfSkp1/GFP, pNC-PfCullin-1/GFPki, and HBglm-PfCul2/cDD_HA_) by the infected RBC (iRBC) method [87,88]. Similarly, *P*. *falciparum* 3D7 early ring stage parasites were transfected with HBglm-PfCul2/cDD_HA_ transfection plasmid. The transfected cultures were maintained in the presence of 1 μg/ml blasticidin (pNC-PfRbx1myc, pNC-PfSkp1/GFP and pNC-PfCullin-1/GFPki) or 1 nM WR99210 (HBglm-PfCul2/cDD_HA_) from day 2–12, followed by no drug till day 18, and then again in the presence of the drug until the emergence of resistant parasites. For pNC-PfCullin-1/GFPki and HBglm-PfCul2/cDD_HA_ transfected parasites, genomic DNA was isolated at every 3–4 weeks intervals and assessed for plasmid integration into the target site by PCR using primer sets specific for 5’-integration (PfCullin-1: Cull1-FseqP3/GFPSeqR1, PfCullin-2: PfCul2-5con/PvAc-Rseq), 3’-integration (PfCullin-1: PcDT5U-RP/PfCul1-3con, PfCullin-2; Hrp2-seqF/PfCul2-3con), wild type locus (PfCullin-1: Cull1FseqP1/PfCul1-3con, PfCullin-2: PfCullin2-5con/PfCullin2-3con) and a positive control target (PfCullin-1: PfRbxF/PfRbxR, PfCullin-2: PfAtg18-5con/3con). Once plasmid integration at the target locus was observed (transfected with pNC-PfCullin-1/GFPki or HBglm-PfCul2/cDD_HA_), parasites were cloned by dilution cloning method, and cloned lines were again evaluated for the presence of integration locus by PCR. A GFP-expressing parasite line (GFPcon) was used as a control in various experiments, which has been described earlier [86].

#### Expression and localization of proteins

Recombinant parasites carrying the pNC-PfRbx1myc (PfRbx1myc-epi) or pNC-PfSkp1/GFP (PfSkp1GFP-epi) plasmid would express PfRbx1myc or PfSkp1GFP fusion protein, respectively. Cloned recombinant parasites with integration at the PfCullin-1 locus (PfCul1GFPKI) of PfCullin-2 locus (PfCul2KD_D10_ and PfCul2KD_3D7_) would express PfCullin-1/GFP or PfCullin-2/cDD_HA_ fusion protein, respectively. Recombinant parasites were evaluated for expression and localization of the target fusion proteins. Mixed stage cultures of wild type *P*. *falciparum* D10, GFPcon, PfRbx1myc-epi and PfSkp1GFP-epi parasites were harvested at 10% parasitemia. The parasite pellets were resuspended in 4× pellet volume of the SDS-PAGE sample buffer (1× buffer contains 50 mM Tris-HCl, 20% glycerol, 2% SDS, 1% β-mercaptoethanol, 0.01% bromophenol blue, pH 6.8), the lysates were run on 12% SDS-PAGE, and the proteins were transferred onto the Immobilon-P membrane. The membrane was blocked (blocking buffer contained 3% skim milk in TBS-T (10 mM Tris, 15 mM NaCl, 0.1% Tween-20, pH 7.4)), incubated with mouse anti-Myc (for PfRbx1myc) or rabbit anti-GFP (for PfSkp1GFP) antibodies (at 1/1000 dilution in blocking buffer), followed by HRP-conjugated goat anti-mouse or anti-rabbit IgG (at 1/20000 dilution in blocking buffer). The signal was developed using the SuperSignal Chemiluminescent Substrate. The blot was stripped and re-probed for β-actin as a loading control with mouse anti-β-actin-HRP antibodies (at 1/30000 dilution in blocking buffer).

Cultures of the PfCul1GFPKI, PfCul2KD_D10_ and PfCul2KD_3D7_ parasites were synchronized and harvested at ~10% parasitemia at different time points during the 48 hour-long asexual erythrocytic development. The parasite pellets were processed for western blotting using mouse anti-HA (for PfCul2KD_D10_ and PfCul2KD_3D7_, at 1/1000 dilution in blocking buffer) or rabbit anti-GFP (for PfCul1GFPKI, at 1/1000 dilution in blocking buffer) antibodies, followed by HRP-conjugated goat anti-mouse or anti-rabbit IgG (at 1/20000 dilution in blocking buffer) as has been described above for PfRbx1myc-epi and PfSkp1GFP-epi parasites. The blot was stripped and re-probed for β-actin with mouse anti-β-actin-HRP antibodies (at 1/30000 dilution in blocking buffer).

For localization of target proteins by immunofluorescence assay (IFA), synchronized cultures of recombinant parasites (PfRbx1myc-epi, PfCul2KD_D10_ and PfCul2KD_3D7_) were collected at various stages during the 48 hour-long asexual erythrocytic development, washed with PBS, immobilized on a poly L-Lysine coated slide, fixed (3% paraformaldehyde and 0.01% glutaraldehyde), permeabilized with 0.1% Triton X-100 (v/v in PBS), and blocked (blocking buffer contained 3% BSA in PBS). The slides were incubated with mouse anti-Myc or mouse anti-HA (1/100 in blocking buffer) antibodies, followed by Cyanine 3-conjugated goat anti-mouse IgG (at 1/1000 dilution in blocking buffer) and DAPI (10 μg/ml). The slides were air-dried, mounted with ProLong Gold anti-fade reagent, and covered with a coverslip. The edges of the mounted area were sealed with nail polish, the slide was observed under 100× objective of ZEISS Axioimager microscope fitted with Zeiss AxioCam HRm camera to capture images using Zeiss AxioCam HRm, which were analysed using the Axiovision software. For live-cell fluorescence microscopy, different stages of PfSkp1GFP-epi and PfCul1GFPKI parasites were collected, immobilized on a poly L-Lysine coated slide, and stained with Hoechst (1 μg/ml). The slides were observed, images were captured and analysed as has been described for IFA. For confocal microscopy, the IFA slides (PfRbx1myc-epi and PfCul2KDD_10_) and fixed slides (PfSkp1GFP-epi and PfCul1GFPKI) were viewed using the Leica TCS SP8 confocal laser scanning microscope, Z-section images were captured, and edited using the Leica Application Suite, Fiji and Adobe Photoshop softwares.

#### Fractionation of recombinant parasites

We prepared cytosolic and chromatin fractions of recombinant parasites by chromatin enrichment for proteomics (ChEP) method [89], and evaluated the fractions for the presence of target proteins by western blotting. Briefly, a 50 ml synchronous culture (at ~10% parasitemia) of each of the PfRbx1myc-epi, PfSkp1GFP-epi, and PfCul1GFPKI parasites was grown in the presence of 1 μg/ml blasticidin till 30-hour stage, and parasites were purified by saponin lysis method. Similarly, a 50 ml synchronous culture of PfCull2KD_D10_ parasites was grown with 10 μM trimethoprim till 30-hour stage, and parasites were purified by saponin lysis method. The parasite pellets were fixed (1% formaldehyde in PBS), and treated with 0.125 M glycine (in PBS) to quench the reactive groups. The cells were resuspended in 500 μl of nuclear extraction buffer (25 mM Tris-Cl, 10 mM KCl, 0.1 mM EDTA, 0.1 mM EGTA, 1 mM DTT, pH 7.4, protease inhibitor cocktail, and 0.25% NP-40), the lysate was passed twice through a 25G needle, centrifuged at 2300g for 20 min at 4°C, and the supernatant was saved as a cytosolic fraction. The pellet, which contains nuclei, was washed with 500 μl of nuclear extraction buffer with RNAse A (200 μg/ml) followed by twice with PBS. The pellet was washed with Tris-SDS buffer (50 mM Tris-Cl, 10 mM EDTA, pH 7.4, 4% SDS, protease inhibitor cocktail) and urea buffer (10 mM Tris-Cl, 1 mM EDTA, 8 M urea, pH 7.4). The final pellet was resuspended in 500 μl of storage buffer (10 mM Tris-Cl, 1 mM EDTA, 25 mM NaCl, 10% glycerol, pH 7.4, and protease inhibitor cocktail), and the suspension was sonicated (Diagenode Bioruptor UCD-200) at 4°C for 5 min to solubilize chromatin completely. The sonicated sample was centrifuged at 15000g for 30 minutes at 4°C, the supernatant containing solubilized chromatin was mixed with equal volume of 2× SDS-PAGE sample buffer, and incubated at 98°C for 30 min to reverse the cross-links. The chromatin and cytosolic samples were assessed for purity by western blotting using rabbit anti-Histone 2B (1/1000 diluted in blocking buffer) and rabbit anti-α2 subunit of 20S proteasome (at 1/1000 dilution in blocking buffer) antibodies, followed by appropriate secondary antibodies. Both chromatin and cytosolic samples were evaluated for the presence of PfRbx1myc, PfSkp1GFP, PfCullin-1/GFP and PfCullin-2/cDD_HA_ proteins by western blot using mouse anti-Myc (PfRbx1myc), anti-HA (PfCullin-2/cDD_HA_) or anti-GFP (PfSkp1GFP, PfCullin-1/GFP) antibodies, followed by appropriate HRP-conjugated secondary antibodies as has been described in the “expression and localization of proteins” section.

#### Immunoprecipitation and mass spectrometry

Asynchronous cultures of PfRbx1myc-epi, PfSkp1GFP-epi, PfCul1GFPKI, wild type *P*. *falciparum* D10 and GFP control parasites were harvested at 10–15% parasitemia, and processed for immunoprecipitation as has been described earlier [86]. Briefly, the parasite pellets were resuspended in 5× pellet volume of the lysis buffer (10 mM Tris, 0.5 mM EDTA, 100 mM NaCl, 0.5% NP-40, pH 7.5, EDTA free protease inhibitor cocktail), subjected to freeze-thaw, the suspension was passed through a 26.5G needle, and the sample was incubated in ice for 30 min. The lysate was centrifuged at 25000g for 30 min at 4°C, the supernatant was transferred to a fresh tube, the pellet was re-extracted with 3× pellet volume of the lysis buffer and this supernatant was combined with the first supernatant. The supernatant was incubated with Myc-Trap (PfRbx1myc-epi and wild type *P*. *falciparum* D10) or GFP-Trap (PfSkp1GFP-epi, PfCul1GFPKI, and GFP control) antibodies (15 μl slurry/2 mg protein; ChromoTek) for 2 hours at 4°C with gentle shaking. The flow through was discarded, and the beads were washed with wash buffer 1 (10 mM Tris, 100 mM NaCl, 0.5 mM EDTA, pH 7.5, EDTA free protease inhibitor cocktail) followed by wash buffer 2 (10 mM Tris, 150 mM NaCl, 0.5 mM EDTA, pH 7.5, EDTA free protease inhibitor cocktail). The beads were boiled in 100 μl of 2× SDS PAGE sample buffer for 15 min to elute the bound proteins. A 20 μl aliquot of the eluate along with appropriate controls (input, flow through and washes) were checked for the presence of bait by western blotting using mouse anti-Myc or rabbit anti-GFP antibodies, followed by with appropriate secondary antibodies as described in the “expression and localization of proteins” section. The remaining eluate was processed for LC-MS/MS.

Asynchronous cultures of wild type *P*. *falciparum* D10 and PfCull2KD_D10_ (cultured with 10 μM trimethoprim) parasites were harvested at 10–15% parasitemia, the parasite pellets were lysed (lysis buffer: 10 mM Tris-Cl, 150 mM NaCl, 0.5 mM EDTA, 0.1% NP-40, 10% glycerol, pH 7.5, protease inhibitor cocktail), and processed further as has been described above. The supernatant was incubated with 5 μg of rabbit anti-HA antibodies for overnight with gentle mixing, the suspension was mixed with 10 μl of protein A/G magnetic beads (pre-equilibrated with wash buffer (10 mM Tris, 150 mM NaCl, 0.5 mM EDTA, 0.1% NP-40, 10% glycerol, pH 7.5)) for 2 h at 4°C, and processed further until the elution step as has been described above. A 20 μl aliquot of the eluate along with appropriate controls (input, flow through, washes) were assessed for the presence of PfCullin-2/cDD_HA_ by western blotting using mouse anti-HA antibody, followed by appropriate secondary antibody. The remaining eluate was processed for LC-MS/MS.

For LC-MS/MS, 80 μl of the eluate was stacked on a gradient SDS-PAGE gel until the protein marker ladder started resolving. The protein band was excised, digested with trypsin, peptides were extracted, vacuum dried, resuspended in 11 μl of 2% formic acid, and a 10 μl of the peptide sample was run on the Q Exactive HF (Thermo Fischer Scientific) to perform HCD mode fragmentation and LC-MS/MS analysis as has been described earlier [86]. The raw data files were acquired on the proteome discoverer v2.2 (Thermo Fischer Scientific), analysed, and the predicted peptide sequences were searched against the Uniprot database of *P*. *falciparum* 3D7 using the Sequest HT algorithm. The analysis criteria used were trypsin specificity, maximum two missed cleavages, and some variable modifications (oxidation of methionine, carbamidomethylation of cysteine, deamidation of asparagine/glutamine). Other criteria were precursor tolerance of 5 ppm, fragmentation tolerance of 0.05 Da, and 1% peptide FDR threshold. The protein hits from Rbx1myc and PfCullin-2/cDD_HA_ data were compared with those from the wild type *P*. *falciparum* D10 data. The proteins identified in PfSkp1GFP and PfCullin-1/GFP immunoprecipitates were compared with the proteins identified in GFPcon sample. Proteins exclusively present in PfRbx1myc, PfSkp1GFP, PfCullin-1/GFP, and PfCullin2/cDD_HA_ samples were considered genuine if reproducibly present in at least 2 of the 3 biological replicates with minimum 1 unique peptide.

We performed gene ontology analysis of the proteins exclusively present in the PfCullin-2 immunoprecipitates using ShinyGO (http://bioinformatics.sdstate.edu/go74/). The data was sorted on the basis of fold-enrichment and the number of proteins from IP/MS results involved in that pathway.

#### Production of recombinant proteins

The complete coding regions of PfRbx1, *P*. *falciparum* calcyclin binding protein (PfCacyBP), PfSkp1, and the N-terminal coding region of PfCullin-1 (PfCul1N, 1–300 aa) were PCR amplified from *P*. *falciparum* genomic DNA using PfRbx-F/PfRbx-R, PfCal-Fexp/PfCal-Rexp, PfSkp1-Fexp/PfSkp1-Rexp, PfNCul1-F/PfNCul1-R primers, respectively. The PfCullin-1 C-terminal coding region (PfCul1C, 601–829 aa) and complete coding region of PfFBXO1 were amplified from *P*. *falciparum* cDNA using PfC-Cul1-Fexp/PfC-Cul1-Rexp and PfFbox-Fexp/PfFbox-Rexp primers, respectively. The PCR fragments were cloned into the pJET1.2 vector, sequenced to ensure that they were free of mutations, and subcloned into the pGEX-6P-1 expression vector at BamHI/XhoI site to obtain pGEX6P1/PfRbx1, pGEX6P1/PfCul1N, pGEX6P1/PfCul1C, pGEX6P1/PfCacyBP, pGEX6P1/PfSkp1, and pGEX6P1/PfFBXO1 expression plasmids. These expression plasmids were transformed into BL21(DE3) *E*. *coli* cells, which would express the recombinant proteins with an N-terminal GST-tag (GST/Cul1N, GST/Cul1C, GST/FBXO, GST/CacyBP, and GST/Rbx). We also transformed BL21(DE3) *E*. *coli* cells with pGEX6P-1 to produce GST protein. GST, GST/Rbx, GST/Cul1N and GST/Cul1C were purified from the soluble fraction of the respective IPTG-induced cells under native conditions using the Protino Glutathione Agarose 4B resin as recommended by the manufacturer and reported earlier [86]. Briefly, the induced cell pellet was resuspended in lysis buffer (PBS with 1 mM DTT and 1 mg/ml lysozyme), the lysate was sonicated (5 s pulses at 20% amplitude for 5–30 min depending on the sample volume), centrifuged at 18000g for 30 min at 4°C, and the supernatant was incubated with Protino Glutathione Agarose 4B. The resin was washed with PBS and bound proteins were eluted (50 mM Tris, 20 mM GSH, pH 7.5). Elution fractions were run on 12% SDS–PAGE, and fractions containing pure or enriched in recombinant protein were pooled and concentrated (30 kDa cut off Amicon Ultra centricon) with simultaneous buffer exchange to 20 mM Tris–Cl, 50 mM NaCl, pH 8.0 at 4°C. The protein amount in the concentrated sample was estimated by BCA assay, and the proteins were stored at -80°C until further use.

GST/FBXO and GST/CacyBP were purified under denaturing conditions from the respective IPTG-induced cells. Briefly, the induced cell pellet was resuspended in lysis buffer (PBS with 10 mM DTT and 8M urea; 5 ml/g weight of the pellet), sonicated (5 s pulses at 20% amplitude for 5–30 min depending on the sample volume), centrifuged, and the supernatant was dialysed sequentially against decreasing concentrations of the urea buffer (6 M, 5 M, 4 M, 3 M, and 2 M urea in PBS with 10 mM DTT, each for every 2 hours at 4°C). The dialysed sample was incubated with Protino Glutathione Agarose 4B (pre-equilibrated with 2 M urea in PBS with 10 mM DTT, 0.125 ml resin/5g of initial cell pellet), the resin was washed with 2 M urea buffer, and bound proteins were eluted (2M urea buffer in PBS with 100 mM GSH). Elution fractions were run on 12% SDS–PAGE, and fractions containing pure or enriched in the recombinant protein were pooled, concentrated (30 kDa cut off Amicon Ultra centricon) with simultaneous buffer exchange to 20 mM Tris-Cl, 50 mM NaCl, pH 8.0 at 4°C, the protein amount was estimated by BCA assay, and stored at -80°C until further use.

PfSkp1 was also subcloned into the pET28a at BamHI/XhoI sites to obtain pET28a-PfSkp1, which was transformed into Rosetta-gami 2(DE3) pLysS cells to express with N-terminal His-tag (_His_Skp). Recombinant _His_Skp was purified from the soluble fraction of IPTG-induced cells using Ni-NTA chromatography under native conditions as has been described earlier [90]. The eluates were run on a 12% SDS-PAGE, fractions containing pure proteins were pooled and concentrated (10 kDa cut off Amicon Ultra centricon) with simultaneous buffer exchange to 20 mM Tris-Cl, 50 mM NaCl, pH 8.0 at 4°C. The protein amount was estimated by BCA, and the concentrated protein sample was stored at -80°C until further use.

PfRbx1 was also subcloned into pSMO at StuI/HindIII sites to obtain pSMO-PfRbx1 plasmid and transformed into Rosetta-gami 2(DE3) pLysS cells, which would express it with an N-terminal His-SUMO-tag (_His_SUMO/Rbx). The pSMO vector has been reported earlier [45]. The recombinant _His_SUMO/Rbx protein was purified from the soluble fraction of IPTG-induced cells by Ni-NTA affinity chromatography under native conditions as has been described earlier [45]. Elution fractions containing the pure protein were pooled and concentrated (10 kDa cut off Amicon Ultra centricon) with simultaneous buffer exchange to 20 mM Tris-Cl, 50 mM NaCl, pH 8.0 at 4°C. The protein amount was estimated by BCA, and the concentrated protein sample was stored at -80°C until further use. In parallel, we also transformed Rosetta-gami 2(DE3) pLysS cells with pSMO plasmid, and purified the _His_SUMO protein as has been reported previously [45].

#### Dot blot protein overlay assay

For assessing interaction of recombinant _His_PfSkp1 with recombinant GST, GST/PfRbx1, GST/PfCacyBP, GST/PfCul1N and GST/FBXO1 proteins, different amounts of purified _His_PfSkp1 were spotted on the nitrocellulose membrane, the membrane was blocked (3% BSA in PBS-T), and then overlaid with 10 μg of purified GST, GST/PfRbx1, GST/PfCacyBP, GST/PfCul1N or GST/FBXO1 for 12 h at 4°C with gentle shaking. The membrane was washed with PBS-T, incubated with mouse anti-GST antibodies (at 1/5000 in PBS-T), followed by HRP conjugated goat anti-mouse antibodies. The signal was developed using the SuperSignal West Pico Chemiluminescent kit. For interaction of recombinant _His_SUMO/PfRbx1 with recombinant GST/PfCul1C, GST/PfCacyBP, GST/PfFBXO1 and GST proteins, different amounts of purified _His_SUMO/PfRbx1 were spotted on the nitrocellulose membrane, the membrane was blocked, overlaid with 10 μg of individual recombinant protein, and processed further as has been described above for _His_PfSkp1. As a control to rule out interaction between GST and SUMO tags, different amounts of recombinant _His_SUMO protein was spotted on the nitrocellulose membrane, the membrane was blocked, and overlaid with 10 μg of recombinant GST/PfCul1C or GST protein, and processed further as has been described above for GST/PfSkp1.

#### Co-immunoprecipitation of PfCullin-2 and PfRbx1

Asynchronous cultures of PfRbx1myc-epi and PfCul2KD_D10_ parasites were harvested at ~15% parasitemia, the parasites were purified by the saponin lysis method, and the parasite pellets were resuspended in 4× pellet volume of the lysis buffer (10 mM Tris-Cl, 150 mM NaCl, 0.5 mM EDTA, 0.1% NP-40, 10% glycerol, pH 7.5, protease inhibitor cocktail). Equal amounts of the lysates were mixed, supplemented (with 2 mM ATP, 5 mM MgCl_2_ and 0.2 mM DTT), incubated at room temperature for 2 hrs with gentle mixing, and processed for immunoprecipitation using Myc-Trap antibodies as has been described in the “immunoprecipitation and mass spectrometry” section. The eluate and appropriate controls (input, flow through, and washes) were assessed for the presence of PfRbx1myc by western blotting using mouse anti-Myc antibodies, followed by appropriate secondary antibodies as has been described in the “expression and localization of proteins” section. The blot was stripped and re-probed to check for co-immunoprecipitation of PfCullin-2/cDD_HA_ using rabbit anti-HA antibodies, followed by appropriate secondary antibodies as has been described in the “expression and localization of proteins” section.

#### Ubiquitination activity assays of PfSCF and PfCRL4

PfSkp1GFP-epi and PfCull2KD_D10_ parasites were processed for immunoprecipitation of PfSkp1GFP and PfCullin-2/cDD_HA_, respectively, before the elution step as has been described in the “immunoprecipitation and mass spectrometry” section. The bead-bound PfSkp1GFP and PfCullin-2/cDD_HA_ immunoprecipitates were assessed for ubiquitination activity using a ubiquitin conjugation initiation kit according to the manufacturer’s instructions (Boston Biochem). Briefly, each bead-bound immunoprecipitate was resuspended in 36 μl of the supplied ubiquitination reaction containing ubiquitin E1 enzyme, ubiquitin E2 enzyme, ubiquitin, and 0.5 mM DTT. 1 μg of recombinant GST/PfRbx1 was also added to the PfSkp1GFP ubiquitination reaction. Each reaction was divided into two equal aliquots, one was supplemented with MgCl_2_ and ATP and the other was supplemented with water to make up the volume to 20 μl. Both the reactions were incubated at 37°C for 1 h, stopped by adding 20 μl of 4× SDS-PAGE sample buffer, boiled for 15 minutes, and the supernatants were processed for western blotting using mouse anti-ubiquitin antibodies, followed by with HRP-conjugated goat anti-mouse IgG (at 1/20000 dilution in blocking buffer). The blots were stripped and reprobed for the presence of PfSkp1GFP or PfCullin-2/cDD_HA_ with rabbit anti-GFP antibodies (for PfSkp1GFP) or anti-HA antibodies (for PfCullin-2/cDD_HA_), followed by appropriate secondary antibodies as has been described in the “expression and localization of proteins” section.

#### Effect of PfCullin-2 knock-down on parasite development and ubiquitination activity

PfCull2KD_3D7_ parasites were maintained in the presence of 10 μM trimethoprim (+TMP) as has been described in the “parasite culture” section unless stated otherwise. To check knock-down of PfCullin-2/cDD_HA_ protein, a synchronized culture of PfCull2KD_3D7_ parasites with ~10% rings was divided into two halves. One half was grown with (+TMP) and the other was grown without (-TMP) 10 μM trimethoprim for three consecutive cycles. Equal aliquots of both the cultures were harvested at the trophozoite stage (at ~30 hr) in each cycle, and the parasites were assessed for PfCullin-2/cDD_HA_ protein level by western blotting using mouse anti-HA antibodies, followed by HRP-conjugated goat anti-mouse IgG as described in the “expression and localization of proteins section”. The blot was stripped and re-probed for β-actin as a loading control using mouse anti-β-actin-HRP antibodies.

To assess the effect of PfCullin-2 knock-down on asexual erythrocytic growth, synchronized PfCull2KD_3D7_ parasites were cultured with or without 10 μM TMP in 100 μl volume in a 96-well tissue culture plate, beginning with 4% ring stage parasitemia, for four consecutive cycles. If required, the cultures were diluted 2–4 folds at the end of the 48 hr development cycle and supplemented with fresh medium and RBCs to maintain 3–4% parasitemia. At the end of each cycle, a 50 μl aliquot of each culture was fixed (2% formaldehyde in PBS), stained with YOYO-1 (10 nM YOYO-1 in PBS with 0.1% Triton X-100), and the number of infected cells was determined by counting 10,000 cells using the BD Fortesa FACS Analyzer (λ_ex_ 491 nm and λ_em_ 509 nm). As a control, 100 μl suspension of the day-0 culture was also processed for FACS. The parasitemia at the end of each cycle was adjusted for dilution factor, normalized to day-0 parasitemia, and plotted as fold-multiplication over growth cycles using GraphPad Prism. In parallel, a 50 μl aliquot of each culture at the end of each cycle was processed for Giemsa staining, and assessed for parasite morphology using 100× objective of a light microscope (Axio-Imager Z2).

To evaluate the effect of PfCullin2 knock-down on sexual erythrocytic stages, PfCul2KD_3D7_ gametocytes were obtained by following the “crash method” [91]. Briefly, a synchronized PfCul2KD_3D7_ culture with 5% rings at 4% haematocrit was cultured in the presence of 10 μM TMP until 8–10% parasitemia with daily media change. The culture was divided into two equal aliquots, one was supplemented with 50 mM GlcNAc and 10 μM TMP and the other was supplemented with 50 mM GlcNAc only. Both the cultures were grown for 4 days with daily change of media and the supplements. On day 5 onward, the cultures were maintained with or without TMP, Giemsa-stained smears were made daily, and observed for gametocytes/2000 cells. The number of gametocytes was plotted over days using GraphPad Prism software. On day 4, a 20 ml aliquot of each culture was assessed for PfCullin-2/cDD_HA_ protein level by western blotting using mouse anti-HA antibodies, followed by HRP-conjugated goat anti-mouse IgG as described in the “expression and localization of proteins” section. The blot was stripped and re-probed for β-actin as a loading control with anti-β-actin-HRP antibodies.

For the total ubiquitination activity of PfCull2KD_3D7_ parasites, synchronized ring-stage parasites were cultured with (+TMP) or without (-TMP) 10 μM trimethoprim for 3 consecutive cycles, and equal aliquots of the cultures were harvested at the trophozoite stage (30 hr) in each cycle. For each time point, 1/3^rd^ of the pellet was solubilized in 1× SDS-PAGE sample buffer to obtain whole parasite lysate, and 2/3^rd^ of the pellet was extracted with modified ubiquitination buffer (25 mM Tris, pH 7.6, 50 mM NaCl, 10 mM MgCl2, 0.1% NP-40, 10% glycerol, and protease inhibitor cocktail) to obtain total parasite extract. These extracts were processed for ubiquitination assay with (+ATP) or without (-ATP) ATP as has been described in the “ubiquitination activity assays of PfSCF and PfCRL4” section. The whole cell lysates and ubiquitination reactions were processed for detection of ubiquitinated proteins by western blotting as has been described in the “ubiquitination activity assays of PfSCF and PfCRL4” section.

### Electron microscopy

PfCul2KD_3D7_ parasites were evaluated for morphology and integrity of membraned structures by scanning electron microscopy (SEM) and transmission electron microscopy (TEM). Parasites were processed for SEM as has been reported earlier [92]. Briefly, synchronised PfCul2KD_3D7_ parasites were grown with (+TMP) or without (-TMP) 10 μM trimethoprim until the 3^rd^ cycle late trophozoite/schizont stage, and 10 ml culture with ~10% parasitemia was processed for saponin lysis to purify the parasites. The purified parasites were washed with 500 μl of RPMI 1640, resuspended in 50 μl RPMI 1640, and mixed with 50 μl fixative (2.5% paraformaldehyde, 5% glutaraldehyde, 0.2 M sodium cacodylate buffer, pH 7.4), and incubated for 1 hr at room temperature with slow mixing. The cells were washed twice with sodium cacodylate buffer and gradually dehydrated by incubating with increasing concentration of ethanol (50%, 70%, 95%, and 100%), each incubation was for 3 minutes with fresh solvent. The cells were resuspended in 100 μl water, an aliquot was diluted 100-fold with water and dried on a glass coverslip at room temperature. The coverslip was mounted on the stub and gold spurring was done using the Quorum 150T ES plus. The cells were observed in scanning electron microscope (Hitachi science systems) at 3–5 μm resolution.

For TEM, synchronised PfCul2KD_3D7_ parasites were grown with (+TMP) or without (-TMP) 10 μM trimethoprim until the 3^rd^ cycle late trophozoite/schizont stages, and the parasites were fixed as has been described above for SEM. The fixed cells were washed three times with 0.1 M cacodylate buffer (pH 7.4), and incubated in 1% osmium tetroxide for 30 min to 1 hr at room temperature. The cells were washed twice with cacodylate buffer, stained *en bloc* with 2% uranyl acetate for 20 min, washed twice with the cacodylate buffer, and gradually dehydrated with increasing concentration of ethanol (50%, 70%, 95%, and 100%), each incubation was for 3 min with fresh solvent. The dehydrated sample was incubated and washed twice in propylene oxide for 5 min each. The sample was incubated with a 1:1 and 3:1 mixture of Spurr’s epoxy resin and propylene oxide for 15 and 20 min, respectively, followed by a final wash with pure epoxy resin. Finally, the cells were embedded in epoxy resin with polymerising agent and kept at 62°C for 12–14 hr. The sample was trimmed and sectioned with a glass knife using the ultramicrotome (LEICA EM UC7), and placed on a copper grid. The sample grids were post-stained with uranyl acetate for 1 hr, followed by with lead citrate for 2 min with intermittent washing with water. The sample grids were dried, loaded on the transmission electron microscope (Talos L120C), images were captured with CETA camera at 500 nm, and analysed by Acquisition-Velox software.

#### Label free quantification of proteins

Synchronized PfCull2KD_3D7_ parasites were cultured with (+TMP) or without (-TMP) 10 μM trimethoprim until the 3^rd^ cycle late trophozoite/early schizont stage, and parasites were purified by saponin lysis method. The parasite pellets were resuspended in lysis buffer (100 mM Tris, 4% SDS, 200 mM DTT, 1% Triton X-100, pH 7.5), centrifuged, and the supernatant protein content was estimated by amido black assay. 150 μg of the +TMP or -TMP parasite lysate was mixed with 0.02% bromophenol blue (with 10% glycerol), and ran on 12% SDS-PAGE gel. The gel was stained and each protein lane was cut into 8 slices, and each slice was processed for mass spectrometry as has been described in the “immunoprecipitation and mass spectrometry” section. The MS data of each of the 8 slices of +TMP and -TMP samples were run separately against the *P*. *falciparum* Uniprot database, and abundance values were obtained using the label free quantification tool of Sequest HT. The abundance values from two biological replicates were analysed to obtain log2-fold change of protein levels in -TMP sample as compared to the +TMP sample, and *P*-values for significance of the difference between +TMP and -TMP data sets were calculated using the Student’s T-test. A log2-fold change of ≥+1 was considered as upregulated, ≤-1 was considered as downregulated, and that between +1 to -1 was considered as unchanged. Upregulated and downregulated proteins with *P*-values ≤0.05 were further analysed for differential regulation using the VolcanoseR online tool, and pathway enrichment was obtained by gene set enrichment analysis (GSEA) with the programme R.

#### Assessment of ER stress, DNA damage and mitochondrial membrane potential

ER stress was measured as has been described earlier [93]. Briefly, PfCul2KD_3D7_ parasites were grown with (+TMP) or without (-TMP) 10 μM trimethoprim for 3 cycles. Equal number of 3^rd^ cycle parasites of both the cultures were incubated with different concentrations of DTT (5, 10, and 20 mM) or equal amount of water for 1 hr, followed with 10 μM of the peroxide sensitive dye 2’,7’-dichlorodihydrofluorescein diacetate acetyl ester (H_2_DCFDA) for 30 min in the dark with gentle shaking at room temperature. The fluorescence was measured using the SpectraMax iD3 multimode plate reader (λ_em_ = 530 nm and λ_ex_ = 488 nm).

For measuring the effect of DNA-damaging agent, synchronised PfCul2KD_3D7_ parasites were grown with (+TMP) or without (-TMP) 10 μM trimethoprim until the 3^rd^ cycle mid trophozoite stage, followed by in the presence of 0.005% DMSO or 0.005% MMS for 6 hr. The cultures were washed with cultured medium, supplemented with fresh medium, grown further for 12 hrs, Giemsa stained smears were made, and about 1000 cells were observed for fresh rings.

Mitochondrial stress was measured as membrane potential by treating parasites with JC-1, a cationic mitochondrial membrane potential dye. It forms red aggregates in healthy/polarised mitochondria and remains as a green monomer if mitochondria are unhealthy. The ratio of red/green fluorescence indicates the health status of mitochondria. Asynchronous cultures of PfCul2KD_3D7_ parasites were grown with (+TMP) or without (-TMP) 10 μM trimethoprim for 3 cycles, and equal number of the 3^rd^ cycle parasites were incubated in 2 μM JC-1 (in RPMI 1640) for 20 min at 37°C. The parasites were washed with culture medium, and fluorescence was measured using SpectraMax iD3 multimode plate reader (red: λ_em_ = 590 nm and λ_ex_ = 514 nm; green: λ_em_ = 529 nm and λ_ex_ = 514 nm).

#### Assessment of DNA replication and mitotic structures

PfCul2KD_3D7_ parasites were evaluated for DNA replication by estimating the total DNA content and the number of nuclei. Synchronized PfCul2KD_3D7_ parasites were grown with (+TMP) or without (-TMP) 10 μM trimethoprim for 3 cycles, beginning with equal parasitemia. Equal aliquots of the cultures were taken at 10, 36, and 48 hrs during the first 2 cycles and at 10, 28, 32, 36, 40, and 44 hrs during the 3^rd^ cycle. Half of the each aliquot was fixed (4% paraformaldehyde in PBS) for 45 min at room temperature, treated with 0.1% Triton X-100 (in PBS) to permeabilize the cells, treated with RNase A (0.3 mg/ml) for 20 min at 37°C to remove RNA, and washed with PBS. The samples were stained with SYBR green I (1:2000 v/v in PBS) and the fluorescence of each sample was measured using SpectraMax iD3 multimode plate reader (λem = 530 nm and λex = 488 nm). For microscopy, parasites from the other half of the 3^rd^ cycle aliquots were smeared on glass slides, fixed (3% paraformaldehyde and 0.01% glutaraldehyde in PBS), permeabilized with 0.1% Triton X-100 (v/v in PBS), stained with DAPI, observed under 100× objective of ZEISS Axioimager, and images were captured with Zeiss AxioCam HRm camera. The number of nuclei/cell were counted for each time point.

For the analysis of mitotic structures (spindle fibres and centriolar plaques), parasites were collected at 40 hr stages during the 3^rd^ cycle, and processed for IFA as has been described in the “expression and localization” section. Briefly, for spindle fibres, parasites were stained with alexa-488-conjugated mouse anti-α-tubulin antibodies (at 1/1000 dilution in blocking buffer) and DAPI (10 μg/ml). For centriolar plaques, parasites were stained with rabbit anti-centrin-1 antibodies (at 1/250 dilution in blocking buffer), followed by with alexa-488-conjugated goat anti-rabbit IgG (at 1/1000 in blocking buffer) and DAPI (10 μg/ml). The slides were observed under 100× objective of ZEISS Axioimager and images were captured with Zeiss AxioCam HRm camera. About 100 parasites were observed for staining patterns.

#### Statistical analysis

The data were analyzed to determine significance of difference (*P*-value) between two groups using the t-test. Statistical significance is indicated for data sets showing significant *P*-values (* is p<0.05–0.01, ** is p<0.01–0.001, *** is p<0.001).

## Supporting information

S1 Fig Domain organisation and phylogenetic relatedness of apicomplexan cullin homologs. The amino acid sequences of apicomplexan cullin homologs were analysed for conserved domains and phylogenetic relatedness within apicomplexa and with homologs in model organisms. **A.** The schematic represents domain organization of the indicated cullins with the boundaries of cullin homology domain (CH) and neddylation motif (NM). The red bar in NM is the conserved putative lysine residue that is conjugated to NEDD8. **B.** The phylogram shows that PfCulin-1 (in pink) or PfCulin-2 (in blue) homologs of the indicated apicomplexan parasites cluster together on separate nodes. The cullins (in green) represent the third cullin homolog in the indicated parasites. The annotations are: CHP, conserved hypothetical protein; UP, unspecified product; CFP, cullin-family protein; HP, hypothetical protein; CDCP, cullin domain-containing protein; CLP, cullin like protein; CH, cullin homology domain; Cul, cullin. **C.** The phylogram of PfCullins and their homologs in model organisms shows that PfCullin-1 and PfCullin-2 are distantly related to their homologs in the indicated model organisms. The number on branches denote the strength of clustering (bootstrap value).
(TIF)

S2 Fig Domain organisation of Rbx1 and Skp1 homologs. The amino acid sequences of putative apicomplexan Rbx1 and Skp1 were analysed for conserved domains and phylogenetic relatedness within apicomplexa. **A.** The schematic represents domain organization of the indicated Rbx1 proteins with the boundary of RING_Ubox superfamily (RING_Ubox SF) domain, which contains the consensus zinc finger motif [C-X2-C-X(9–39)-C-X(1–3)-H-X(2–3)-(N/C/H)-X2-C-X(4–48)-C-X2-C]. **B.** The schematic represents domain organization of the indicated Skp1 proteins with the boundaries of the BTB (Broad-Complex, Tramtrack and Bric a brac)/POZ (poxvirus and zinc finger) domain (BPD), Skp1 superfamily (SS) domain, and the sequence shared by BPD and SS regions.
(TIF)

S3 Fig Generation of PfCul1GFPKI and PfCul2KD_D10_ parasites. The endogenous PfCullin-1 and PfCullin-2 coding regions were replaced by PfCullin-1/GFP and PfCullin-2/cDD_HA_ coding sequences in *P*. *falciparum* D10 strain, respectively. Cloned recombinant lines were assessed for integration of the desired DNA into the target locus by PCR and expression of the fusion protein by western blotting. **A.** The schematic represents integration of the transfection plasmid DNA (Trf pDNA) into the endogenous PfCullin-1 locus (WT locus), generating the integration locus (Int locus). Rectangular boxes represent coding regions for PfCullin-1, PfCullin-1-fl1, GFP and BSD. The untranslated regions of PfCullin-1 (5′U and 3′U), the location and orientation of primers (horizontal arrows), the restriction endonuclease sites (vertical lines), and regulatory regions in the linear transfection construct (PfHsp86-3′U, PcDT-5′U and PfHrp2-3′U) are indicated. **B.** The ethidium bromide-stained agarose gel shows PCR products amplified from the genomic DNAs of wild type (WT) and PfCul1GFPKI (Cul1) parasites using locus specific primers (P1 and P2 for 5’ integration (5’-Int), P3 and P4 for 3’ integration (3’-Int), P1 and P4 for wild type (WTsp), and primers specific for the PfRbx1 gene were used as a positive control (Con)). **C.** The lysates of wild type *P*. *falciparum* D10 (WT) and PfCul1GFPKI (Cul1) parasites were assessed for expression of PfCullin-1/GFP by western blotting using anti-GFP (ab-GFP) and anti-β-actin (ab-Ac) antibodies. The arrow indicates the size of truncated PfCullin-1/GFP, and the sizes of protein markers (M) are in kDa. **D.** The schematic represents integration of the linear transfection construct (Trf cassette) into the endogenous PfCullin-2 locus (WT locus), resulting into the generation of integration locus (Int locus). Rectangular boxes represent the coding regions for PfCullin-2, PfCullin-2-fl1, *E*. *coli* mutant DHFR with HA-tag (cDD_HA_) and human DHFR (HD). The flanking untranslated regions (5′U and 3′U), the location and orientation of primers (horizontal arrows), the restriction endonuclease sites (vertical lines) and regulatory regions in the linear transfection construct (PfCullin-2-flank2 (fl2), PvAc-3′U, PyαTb-5′U, and PfHrp2-3′U (Hrp2-3′U)) are indicated. Locus specific primers (P1 and P2 for 5’ integration (5’-Int), P3 and P4 for 3’ integration (3’-Int), P1 and P4 for wild type (WTsp), and primers specific for the PfAtg18 gene were used as a positive control (Con)) were used in PCR to differentiate integration and wild type loci. **E.** The ethidium bromide-stained agarose gel shows PCR products amplified from the wild type (WT) and PfCul2KD_D10_ (Cul2) genomic DNAs using the indicated primer sets. **F.** The lysates of wild type *P*. *falciparum* D10 (WT) and PfCul2KD_D10_ (Cul2) parasites were assessed for expression of PfCullin-2/cDD_HA_ by western blotting using anti-HA (ab-HA) and anti-β-actin (ab-Ac) antibodies. The arrow indicates the size of full-length PfCullin-2/cDD_HA_. The sizes of protein markers (M) are in kDa.
(TIF)

S4 Fig Immunoprecipitation. Lysates of PfCul1GFPKI (Cullin-1) PfSkp1/GFP-epi (Skp), and GFP-expressing control (GFP) parasites were processed for immunoprecipitation using GFP-Trap antibodies. Lysates of PfRbx1myc-epi (Rbx) and wild type *P*. *falciparum* D10 (WT) parasites were processed for immunoprecipitation using Myc-Trap antibodies. Aliquots of the input lysate (In), flow through (FT), wash (W), and eluate (E) for all the immunoprecipitations were assessed for the presence of target proteins using mouse anti-Myc (for Rbx and WT) or rabbit anti-GFP (for Cullin-1, Skp, GFP) antibodies by western blotting. Arrow heads indicate the target proteins, and sizes of protein markers (M) are in kDa.
(TIF)

S5 Fig Production of recombinant PfSCF subunits. **A.** GST alone or GST-tagged PfCacyBP (GST/CacyBP), PfRbx1 (GST/Rbx), PfCullin-1 N-terminal (GST/Cul1N) and C-terminal (GST/Cul1C) fragments, and PfFBXO1 (GST/FBXO) were expressed in *E*. *coli*. The coomassie stained SDS-PAGE gel contains total lysates of un-induced (UI) and IPTG-induced (In) cells, soluble (Sol) and insoluble (Ins) fractions of the induced cells, and purified GST/CacyBP. The lanes GST, GST/Rbx, GST/Cul1N, GST/Cul1C and GST/FBXO contain the respective purified or enriched proteins. **B.** 6×His-tagged PfSkp1 (_His_Skp) was expressed in *E*. *coli* cells. The coomassie stained SDS-PAGE gel contains total lysates of un-induced (UI) and IPTG-induced (In) cells, soluble (Sol) and insoluble (Ins) fractions of the induced cells, and purified protein (Pur). **C.** 6×His-SUMO-tagged PfRbx1 (_His_SUMO/Rbx) or 6×His-SUMO (_His_SUMO) proteins were expressed in *E*. *coli*. The coomassie stained SDS-PAGE gel contains total lysates of un-induced (UI) and IPTG-induced (In) cells, soluble (Sol) and insoluble (Ins) fractions of the induced cells, and purified _His_SUMO/Rbx (Rbx) and _His_SUMO (SUMO) proteins. The protein size markers (M) are in kDa.
(TIF)

S6 Fig Immunoprecipitation of PfCullin-2. Lysates of PfCul2KD_D10_ (**A**) and wild type *P*. *falciparum* D10 (**B**) parasites were processed for immunoprecipitation using rabbit anti-HA antibodies. Aliquots of the parasite lysate inputs (In), flow through (FT), washes (W1, W2), and eluate (E) samples were probed with mouse anti-HA antibodies by western blotting. The sizes of protein markers (M) are in kDa.
(TIF)

S7 Fig Domain organization of CRL4 subunits. The schematic represents predicted domain architecture of the core subunits of human CRL4B and *P*. *falciparum* CRL4. The human CRL4B contains Cullin-4B (HsCul4B), Rbx1 (HsRbx1), DNA damage-specific binding protein 1b (HsDDB1b) and DDB1-Cullin-4-associated factor 1 (HsDCAF1). The *P*. *falciparum* CRL4 complex appears to contain Cullin-2 (PfCul2), Rbx1 (PfRbx1), cleavage and polyadenylation specificity factor subunit A (PfCPSF_A) and WD40 repeat proteins (PfWD40 A and PfWD40 B). The shaded boxes are conserved domains/motifs, which include cullin homology domain (CH) and neddylation motif (NM) in cullins, RING finger domain (R) in Rbx1, methyl methanesulfonate-sensitivity protein 1 (MMS1) and CPSF domains in HsDDB1 and PfCPSF_A proteins, WD40 repeat domain in HsDCAF1 and PfWD40 proteins. PfCPSF_A and PfWD40 A proteins also contain PTZ00341 domain of unknown function, which is present in ring-infected erythrocyte surface antigen (RESA) and several other *Plasmodium* proteins. The number below each domain corresponds to the number of boundary amino acid residues. The protein size is scaled to 1 cm for 100 aa; some regions are interrupted by two vertical lines to fit the protein size.
(JPG)

S8 Fig GO pathway enrichment analysis of the PfCullin-2 interactome. Uniport IDs of all the proteins exclusively present in the PfCullin-2 immunoprecipitate were used to generate biological pathways enrichment. The biological pathways with their fold enrichment and significance (-log10 of FDR) were plotted using the R programme. The plot shows fold enrichment (X-axis) of the pathways (Y-axis) with significant -Log10 FDR.
(TIF)

S9 Fig Generation of PfCul2KD_3D7_ parasites. The endogenous PfCullin-2 coding region was replaced with PfCullin-2/cDD_HA_ coding sequence as shown in SS3D Fig. A cloned line was assessed for the presence of integration locus by PCR and expression of PfCullin-2/cDD_HA_ protein by western blotting. **A.** The ethidium bromide-stained agarose gel shows PCR products amplified from the wild type (WT) and PfCul2KD_3D7_ (Cul2) genomic DNAs using the indicated primer sets. DNA size markers (M) are in kbp. **B.** The lysates of wild type *P*. *falciparum* 3D7 (WT) and PfCul2KD_3D7_ (Cul2) parasites were assessed for expression of PfCullin-2/cDD_HA_ by western blotting using anti-HA (ab-HA) and anti-β-actin (ab-Ac) antibodies. The arrow indicates the size of full-length PfCullin-2/cDD_HA_. **C.** Ring, trophozoite (Troph), and schizont (Sch) stages of PfCul2KD_3D7_ parasites were assessed for localization of PfCullin-2/cDD_HA_ by IFA using anti-HA antibodies. The images are for PfCullin-2/cDD_HA_ signal (Cullin-2), nuclear staining (DAPI), parasite and RBC boundaries (DIC), and overlap of all the three images (Merged). Scale bar is in the merged image.
(TIF)

S10 Fig Scanning electron micrographs of PfCul2KD_3D7_ parasites. PfCul2KD_3D7_ parasites were cultured with (+TMP) or without (-TMP) for three cycles. The 3^rd^ cycle parasites were isolated by saponin lysis and processed for SEM. The images show outer surface of the parasites, which appears smooth in case of +TMP, but distorted in case of -TMP parasites.
(TIF)

S11 Fig Pathway enrichment of differentially regulated proteins in PfCullin-2-depleted trophozoites. PfCul2KD_3D7_ parasites were cultured with or without trimethoprim for three cycles, and the 3^rd^ cycle trophozoites were processed for LFQ. Differentially regulated proteins were used for gene set enrichment analysis (GSEA) using the cluster profiler for gene ontology (GO) term. The plot shows biological pathways on Y-axis and gene enrichment values on X-axis. The size and color of the circle denotes the number of GO terms involved in a particular pathway and their confidence, respectively. Bigger the size and darker the red color of the circle corresponds to higher confidence and enrichment of the pathways.
(JPG)

S12 Fig Effect of PfCullin-2 knock-down on overall ubiquitination. Synchronized PfCull2KD3_D7_ parasites were grown with (+T) or without (-T) trimethoprim for three consecutive cycles (C1, C2 and C3), harvested at the trophozoite stage in each cycle, and processed for PfCullin-2 levels (A), overall ubiquitination profiles (B), and ubiquitination activity (C). **A.** The indicated whole parasite lysates were checked for PfCullin-2 levels (indicated with arrow) using anti-HA antibodies (ab-HA). **B.** The indicated whole parasite lysates were checked for overall ubiquitination profile (indicated with a vertical arrow) using anti-ubiquitin antibodies (ab-Ub). The blot in B was stripped and probed for β-actin levels as a loading control (ab-Ac). **C.** The indicated parasite extracts were assessed for ubiquitination activity without (-A) or with (+A) ATP, and the reactions were processed for ubiquitinated protein levels by western blot using anti-ubiquitin antibodies (ab-Ub). The ubiquitinated proteins are indicated with a vertical arrow. The blots were stripped and probed for β-actin levels as a loading control (ab-Ac). The sizes of protein markers (M) are in kDa.
(TIF)

S1 Table Putative CRL subunits in apicomplexan parasites. PfCullin-1, PfCullin-2, PfRbx1 and PfSkp1 homologs in reference apicomplexan parasite strains of different families.
(DOCX)

S2 Table Proteins identified in the PfCullin1/GFP immunoprecipitate. Shown are the Uniprot (PlasmoDB) ID, score (Sc), coverage (Co) and unique peptides (UP) for each protein identified in three independent biological repeats.
(DOCX)

S3 Table Proteins identified in the PfRbx1myc immunoprecipitate. Shown are the Uniprot (PlasmoDB) ID, score (Sc), coverage (Co) and unique peptides (UP) for proteins present in three independent biological repeats.
(DOCX)

S4 Table Proteins identified in the PfSkp1GFP immunoprecipitate. Shown are the Uniprot (PlasmoDB) ID, score (Sc), coverage (Co) and unique peptides (UP) for each protein present in three independent biological repeats.
(DOCX)

S5 Table Proteins identified in the PfCullin-2/cDD_HA_ immunoprecipitate. Shown are the Uniprot (PlasmoDB) ID, score (Sco), coverage (Cov) and unique peptides (UP) for proteins present in three independent biological repeats. The characterized *Plasmodium* proteins are referenced.
(DOCX)

S6 Table High confidence up- and down-regulated proteins in PfCullin-2-depleted trophozoites. Proteins shown/predicted to be targeted to the apicoplast and mitochondria are highlighted.
(DOCX)

S7 Table Primer used in the study. (DOCX)

## References

[1] World Health Organization. World malaria report 2022. Geneva; 2022.

[2] Skaar JR, Pagano M. Control of cell growth by the SCF and APC/C ubiquitin ligases. Curr Opin Cell Biol. 2009;21(6):816–24. doi: 10.1016/j.ceb.2009.08.004 19775879 PMC2805079

[3] d’Azzo A, Bongiovanni A, Nastasi T. E3 ubiquitin ligases as regulators of membrane protein trafficking and degradation. Traffic. 2005;6(6):429–41. doi: 10.1111/j.1600-0854.2005.00294.x 15882441

[4] Piper RC, Lehner PJ. Endosomal transport via ubiquitination. Trends Cell Biol. 2011;21(11):647–55. doi: 10.1016/j.tcb.2011.08.007 21955996 PMC3225009

[5] Mocciaro A, Rape M. Emerging regulatory mechanisms in ubiquitin-dependent cell cycle control. J Cell Sci. 2012;125(Pt 2):255–63. doi: 10.1242/jcs.091199 22357967 PMC3283867

[6] Jackson SP, Durocher D. Regulation of DNA damage responses by ubiquitin and SUMO. Mol Cell. 2013;49(5):795–807. doi: 10.1016/j.molcel.2013.01.017 23416108

[7] Feng J, Shen WH. Dynamic regulation and function of histone monoubiquitination in plants. Front Plant Sci. 2014;5:83. doi: 10.3389/fpls.2014.00083 24659991 PMC3952079

[8] Ebstein F, Kloetzel PM, Krüger E, Seifert U. Emerging roles of immunoproteasomes beyond MHC class I antigen processing. Cell Mol Life Sci. 2012;69(15):2543–58. doi: 10.1007/s00018-012-0938-0 22382925 PMC11114860

[9] Ng CL, Fidock DA, Bogyo M. Protein degradation systems as sntimalarial therapeutic Targets. Trends in Parasitology. 2017;33(9):731–43. 28688800 10.1016/j.pt.2017.05.009 PMC5656264

[10] Prasad R, Atul, Kolla VK, Legac J, Singhal N, Navale R, et al. Blocking *Plasmodium falciparum* development via dual inhibition of hemoglobin degradation and the ubiquitin proteasome system by MG132. PLoS One. 2013;8(9):e73530. 24023882 10.1371/journal.pone.0073530 PMC3759421

[11] Mata-Cantero L, Chaparro MJ, Colmenarejo G, Cid C, Cortes Cabrera A, Rodriguez MS, et al. Identification of small molecules disrupting the ubiquitin proteasome system in malaria. ACS Infectious Diseases. 2019;5(12):2105–17. doi: 10.1021/acsinfecdis.9b00216 31644867

[12] Simwela NV, Hughes KR, Rennie MT, Barrett MP, Waters AP. Mammalian deubiquitinating enzyme inhibitors display in vitro and in vivo activity against malaria parasites and potentiate artemisinin action. ACS Infectious Diseases. 2021;7(2):333–46. doi: 10.1021/acsinfecdis.0c00580 33400499

[13] Li H, O’Donoghue AJ, van der Linden WA, Xie SC, Yoo E, Foe IT, et al. Structure- and function-based design of *Plasmodium*-selective proteasome inhibitors. Nature. 2016;530(7589):233–6. 26863983 10.1038/nature16936 PMC4755332

[14] Jain J, Jain SK, Walker LA, Tekwani BL. Inhibitors of ubiquitin E3 ligase as potential new antimalarial drug leads. BMC pharmacology & toxicology. 2017;18(1):40. doi: 10.1186/s40360-017-0147-4 28577368 PMC5457628

[15] Kreidenweiss A, Kremsner PG, Mordmüller B. Comprehensive study of proteasome inhibitors against *Plasmodium falciparum* laboratory strains and field isolates from Gabon. Malaria journal. 2008;7:187. 18816382 10.1186/1475-2875-7-187 PMC2569964

[16] Sanchez CP, Liu C-H, Mayer S, Nurhasanah A, Cyrklaff M, Mu J, et al. A HECT ubiquitin-protein ligase as a novel candidate gene for altered quinine and quinidine responses in *Plasmodium falciparum*. PLOS Genetics. 2014;10(5):e1004382. 24830312 10.1371/journal.pgen.1004382 PMC4022464

[17] Dogovski C, Xie SC, Burgio G, Bridgford J, Mok S, McCaw JM, et al. Targeting the Cell Stress Response of *Plasmodium falciparum* to Overcome Artemisinin Resistance. PLOS Biology. 2015;13(4):e1002132. 25901609 10.1371/journal.pbio.1002132 PMC4406523

[18] Bridgford JL, Xie SC, Cobbold SA, Pasaje CFA, Herrmann S, Yang T, et al. Artemisinin kills malaria parasites by damaging proteins and inhibiting the proteasome. Nature communications. 2018;9(1):3801. doi: 10.1038/s41467-018-06221-1 30228310 PMC6143634

[19] Hunt P, Afonso A, Creasey A, Culleton R, Sidhu AB, Logan J, et al. Gene encoding a deubiquitinating enzyme is mutated in artesunate- and chloroquine-resistant rodent malaria parasites. Mol Microbiol. 2007;65(1):27–40. doi: 10.1111/j.1365-2958.2007.05753.x 17581118 PMC1974797

[20] Simwela NV, Hughes KR, Roberts AB, Rennie MT, Barrett MP, Waters AP. Experimentally engineered mutations in a ubiquitin hydrolase, UBP-1, modulate in vivo susceptibility to artemisinin and chloroquine in *Plasmodium berghei*. Antimicrob Agents Chemother. 2020;64(7). 10.1128/AAC.02484-19 PMC7318008 32340987

[21] Komander D, Rape M. The ubiquitin code. Annual review of biochemistry. 2012;81:203–29. doi: 10.1146/annurev-biochem-060310-170328 22524316

[22] Metzger MB, Hristova VA, Weissman AM. HECT and RING finger families of E3 ubiquitin ligases at a glance. J Cell Sci. 2012;125(Pt 3):531–7. doi: 10.1242/jcs.091777 22389392 PMC3381717

[23] Spratt DE, Walden H, Shaw GS. RBR E3 ubiquitin ligases: new structures, new insights, new questions. The Biochemical journal. 2014;458(3):421–37. doi: 10.1042/BJ20140006 24576094 PMC3940038

[24] Cardozo T, Pagano M. Wrenches in the works: drug discovery targeting the SCF ubiquitin ligase and APC/C complexes. BMC Biochem. 2007;8 Suppl 1:S9. doi: 10.1186/1471-2091-8-S1-S9 18047746 PMC2106342

[25] Vodermaier HC. APC/C and SCF: controlling each other and the cell cycle. Curr Biol. 2004;14(18):R787–96. doi: 10.1016/j.cub.2004.09.020 15380093

[26] Petroski MD, Deshaies RJ. Function and regulation of cullin-RING ubiquitin ligases. Nat Rev Mol Cell Biol. 2005;6(1):9–20. doi: 10.1038/nrm1547 15688063

[27] Bosu DR, Kipreos ET. Cullin-RING ubiquitin ligases: global regulation and activation cycles. Cell Div. 2008;3:7. doi: 10.1186/1747-1028-3-7 18282298 PMC2266742

[28] Sarikas A, Hartmann T, Pan Z-Q. The cullin protein family. Genome Biology. 2011;12(4):220. doi: 10.1186/gb-2011-12-4-220 21554755 PMC3218854

[29] Jang SM, Redon CE, Thakur BL, Bahta MK, Aladjem MI. Regulation of cell cycle drivers by Cullin-RING ubiquitin ligases. Exp Mol Med. 2020;52(10):1637–51. doi: 10.1038/s12276-020-00508-4 33005013 PMC8080560

[30] Abbas T, Dutta A. Regulation of mammalian DNA replication via the ubiquitin-proteasome system. Adv Exp Med Biol. 2017;1042:421–54. doi: 10.1007/978-981-10-6955-0_19 29357069 PMC5933532

[31] Zhou P, Yan F. CRL4 ubiquitin pathway and DNA damage response. In: Sun Y, Wei W, Jin J, editors. Cullin-RING ligases and protein neddylation: Biology and Therapeutics. Singapore: Springer Singapore; 2020. p. 225–39. 10.1007/978-981-15-1025-0_14 31898231

[32] Ponts N, Saraf A, Chung DW, Harris A, Prudhomme J, Washburn MP, et al. Unraveling the ubiquitome of the human malaria parasite. J Biol Chem. 2011;286(46):40320–30. doi: 10.1074/jbc.M111.238790 21930698 PMC3220526

[33] Green JL, Wu Y, Encheva V, Lasonder E, Prommaban A, Kunzelmann S, et al. Ubiquitin activation is essential for schizont maturation in *Plasmodium falciparum* blood-stage development. PLoS Pathog. 2020;16(6):e1008640. 32569299 10.1371/journal.ppat.1008640 PMC7332102

[34] Karpiyevich M, Adjalley S, Mol M, Ascher DB, Mason B, van der Heden van Noort GJ, et al. Nedd8 hydrolysis by UCH proteases in *Plasmodium* parasites. PLoS Pathog. 2019;15(10):e1008086. 31658303 10.1371/journal.ppat.1008086 PMC6837540

[35] Jankowska-Döllken M, Sanchez CP, Cyrklaff M, Lanzer M. Overexpression of the HECT ubiquitin ligase PfUT prolongs the intraerythrocytic cycle and reduces invasion efficiency of *Plasmodium falciparum*. Sci Rep. 2019;9(1):18333. 31797898 10.1038/s41598-019-54854-z PMC6893019

[36] Nair SC, Xu R, Pattaradilokrat S, Wu J, Qi Y, Zilversmit M, et al. A *Plasmodium yoelii* HECT-like E3 ubiquitin ligase regulates parasite growth and virulence. Nature communications. 2017;8(1):223. 10.1038/s41467-017-00267-3 PMC5548792 28790316

[37] Maneekesorn S, Knuepfer E, Green JL, Prommana P, Uthaipibull C, Srichairatanakool S, et al. Deletion of *Plasmodium falciparum* ubc13 increases parasite sensitivity to the mutagen, methyl methanesulfonate and dihydroartemisinin. Sci Rep. 2021;11(1):21791. 34750454 10.1038/s41598-021-01267-6 PMC8575778

[38] Artavanis-Tsakonas K, Weihofen WA, Antos JM, Coleman BI, Comeaux CA, Duraisingh MT, et al. Characterization and structural studies of the *Plasmodium falciparum* ubiquitin and Nedd8 hydrolase UCHL3. J Biol Chem. 2010;285(9):6857–66. 20042598 10.1074/jbc.M109.072405 PMC2825479

[39] Frickel E-M, Quesada V, Muething L, Gubbels M-J, Spooner E, Ploegh H, et al. Apicomplexan UCHL3 retains dual specificity for ubiquitin and Nedd8 throughout evolution. Cellular Microbiology. 2007;9(6):1601–10. doi: 10.1111/j.1462-5822.2007.00896.x 17371404

[40] Spork S, Hiss JA, Mandel K, Sommer M, Kooij TW, Chu T, et al. An unusual ERAD-like complex is targeted to the apicoplast of *Plasmodium falciparum*. Eukaryotic cell. 2009;8(8):1134–45. 19502583 10.1128/EC.00083-09 PMC2725561

[41] Agrawal S, Chung D-WD, Ponts N, van Dooren GG, Prudhomme J, Brooks CF, et al. An apicoplast localized ubiquitylation system is required for the import of nuclear-encoded plastid proteins. PLOS Pathogens. 2013;9(6):e1003426. doi: 10.1371/journal.ppat.1003426 23785288 PMC3681736

[42] Guttery DS, Ferguson DJP, Poulin B, Xu Z, Straschil U, Klop O, et al. A putative homologue of CDC20/CDH1 in the malaria parasite is essential for male gamete development. PLOS Pathogens. 2012;8(2):e1002554. doi: 10.1371/journal.ppat.1002554 22383885 PMC3285604

[43] Wall RJ, Ferguson DJP, Freville A, Franke-Fayard B, Brady D, Zeeshan M, et al. *Plasmodium* APC3 mediates chromosome condensation and cytokinesis during atypical mitosis in male gametogenesis. Sci Rep. 2018;8(1):5610. 29618731 10.1038/s41598-018-23871-9 PMC5884774

[44] Rashpa R, Klages N, Schvartz D, Pasquarello C, Brochet M. The Skp1-Cullin1-FBXO1 complex is a pleiotropic regulator required for the formation of gametes and motile forms in *Plasmodium berghei*. Nature communications. 2023;14(1):1312. 10.1038/s41467-023-36999-8 PMC10006092 36898988

[45] Bhattacharjee M, Adhikari N, Sudhakar R, Rizvi Z, Das D, Palanimurugan R, et al. Characterization of *Plasmodium falciparum* NEDD8 and identification of cullins as its substrates. Scientific Reports. 2020;10(1):20220. 33214620 10.1038/s41598-020-77001-5 PMC7677368

[46] Gardner MJ, Hall N, Fung E, White O, Berriman M, Hyman RW, et al. Genome sequence of the human malaria parasite *Plasmodium falciparum*. Nature. 2002;419(6906):498–511. 12368864 10.1038/nature01097 PMC3836256

[47] Bozdech Z, Llinás M, Pulliam BL, Wong ED, Zhu J, DeRisi JL. The Transcriptome of the Intraerythrocytic Developmental Cycle of *Plasmodium falciparum*. PLOS Biology. 2003;1(1):e5. 12929205 10.1371/journal.pbio.0000005 PMC176545

[48] Otto TD, Wilinski D, Assefa S, Keane TM, Sarry LR, Böhme U, et al. New insights into the blood-stage transcriptome of *Plasmodium falciparum* using RNA-Seq. Mol Microbiol. 2010;76(1):12–24. 20141604 10.1111/j.1365-2958.2009.07026.x PMC2859250

[49] Dai Q, Wang H. "Cullin 4 makes its mark on chromatin". Cell Division. 2006;1(1):14. doi: 10.1186/1747-1028-1-14 16831222 PMC1533813

[50] Sweeney MA, Iakova P, Maneix L, Shih FY, Cho HE, Sahin E, et al. The ubiquitin ligase Cullin-1 associates with chromatin and regulates transcription of specific c-MYC target genes. Sci Rep. 2020;10(1):13942. doi: 10.1038/s41598-020-70610-0 32811853 PMC7435197

[51] Jackson S, Xiong Y. CRL4s: the CUL4-RING E3 ubiquitin ligases. Trends in Biochemical Sciences. 2009;34(11):562–70. doi: 10.1016/j.tibs.2009.07.002 19818632 PMC2783741

[52] Schapira M, Tyers M, Torrent M, Arrowsmith CH. WD40 repeat domain proteins: a novel target class? Nature Reviews Drug Discovery. 2017;16(11):773–86. doi: 10.1038/nrd.2017.179 29026209 PMC5975957

[53] Muralidharan V, Oksman A, Iwamoto M, Wandless TJ, Goldberg DE. Asparagine repeat function in a *Plasmodium falciparum* protein assessed via a regulatable fluorescent affinity tag. Proc Natl Acad Sci U S A. 2011;108(11):4411–6. 21368162 10.1073/pnas.1018449108 PMC3060247

[54] Pradhan S, Kalia I, Roy SS, Singh OP, Adak T, Singh AP, et al. Molecular characterization and expression profile of an alternate proliferating cell nuclear antigen homolog PbPCNA2 in *Plasmodium berghei*. IUBMB life. 2019;71(9):1293–301. 30865364 10.1002/iub.2036

[55] Mitra P, Banu K, Deshmukh AS, Subbarao N, Dhar SK. Functional dissection of proliferating-cell nuclear antigens (1 and 2) in human malarial parasite *Plasmodium falciparum*: possible involvement in DNA replication and DNA damage response. The Biochemical journal. 2015;470(1):115–29. 26251451 10.1042/BJ20150452

[56] Wyatt MD, Pittman DL. Methylating agents and dna repair responses: methylated bases and sources of strand breaks. Chemical Research in Toxicology. 2006;19(12):1580–94. doi: 10.1021/tx060164e 17173371 PMC2542901

[57] Ganter M, Goldberg JM, Dvorin JD, Paulo JA, King JG, Tripathi AK, et al. *Plasmodium falciparum* CRK4 directs continuous rounds of DNA replication during schizogony. Nature Microbiology. 2017;2(5):17017. 10.1038/nmicrobiol.2017.17 PMC5328244 28211852

[58] Zeeshan M, Pandey R, Ferguson DJP, Tromer EC, Markus R, Abel S, et al. Real-time dynamics of *Plasmodium* NDC80 reveals unusual modes of chromosome segregation during parasite proliferation. J Cell Sci. 2020;134(5). 10.1242/jcs.245753 PMC7340582 32501284

[59] Morahan BJ, Abrie C, Al-Hasani K, Batty MB, Corey V, Cowell AN, et al. Human aurora kinase inhibitor hesperadin reveals epistatic interaction between *Plasmodium falciparum* PfArk1 and PfNek1 kinases. Communications biology. 2020;3(1):701. 33219324 10.1038/s42003-020-01424-z PMC7679417

[60] Ramachandran S, Ciulli A. Building ubiquitination machineries: E3 ligase multi-subunit assembly and substrate targeting by PROTACs and molecular glues. Curr Opin Struct Biol. 2021;67:110–9. doi: 10.1016/j.sbi.2020.10.009 33271439

[61] Matthews H, Duffy CW, Merrick CJ. Checks and balances? DNA replication and the cell cycle in *Plasmodium*. Parasites & Vectors. 2018;11(1):216. 10.1186/s13071-018-2800-1 PMC5872521 29587837

[62] Seibert V, Prohl C, Schoultz I, Rhee E, Lopez R, Abderazzaq K, et al. Combinatorial diversity of fission yeast SCF ubiquitin ligases by homo- and heterooligomeric assemblies of the F-box proteins Pop1p and Pop2p. BMC Biochem. 2002;3:22. doi: 10.1186/1471-2091-3-22 12167173 PMC128837

[63] Skaar JR, Pagan JK, Pagano M. Mechanisms and function of substrate recruitment by F-box proteins. Nat Rev Mol Cell Biol. 2013;14(6):369–81. doi: 10.1038/nrm3582 23657496 PMC3827686

[64] Matsuzawa SI, Reed JC. Siah-1, SIP, and Ebi collaborate in a novel pathway for beta-catenin degradation linked to p53 responses. Mol Cell. 2001;7(5):915–26. doi: 10.1016/s1097-2765(01)00242-8 11389839

[65] Topolska-Woś AM, Chazin WJ, Filipek A. CacyBP/SIP—Structure and variety of functions. Biochimica et biophysica acta. 2016;1860(1 Pt A):79–85. doi: 10.1016/j.bbagen.2015.10.012 26493724

[66] Jackson S, Xiong Y. Targeting protein ubiquitylation: DDB1 takes its RING off. Nature cell biology. 2009;11(4):379–81. doi: 10.1038/ncb0409-379 19337321 PMC4869686

[67] Kim RQ, Sixma TK. Regulation of USP7: A high incidence of E3 complexes. Journal of molecular biology. 2017;429(22):3395–408. doi: 10.1016/j.jmb.2017.05.028 28591556

[68] de Bie P, Ciechanover A. Ubiquitination of E3 ligases: self-regulation of the ubiquitin system via proteolytic and non-proteolytic mechanisms. Cell death and differentiation. 2011;18(9):1393–402. doi: 10.1038/cdd.2011.16 21372847 PMC3178436

[69] Baptista CG, Lis A, Deng B, Gas-Pascual E, Dittmar A, Sigurdson W, et al. Toxoplasma F-box protein 1 is required for daughter cell scaffold function during parasite replication. PLOS Pathogens. 2019;15(7):e1007946. doi: 10.1371/journal.ppat.1007946 31348812 PMC6685633

[70] Tanneru N, Nivya MA, Adhikari N, Saxena K, Rizvi Z, Sudhakar R, et al. *Plasmodium* DDI1 is a potential therapeutic target and important chromatin-associated protein. International Journal for Parasitology. 2023;53(3):157–75. 36657610 10.1016/j.ijpara.2022.11.007

[71] Florentin A, Cobb DW, Fishburn JD, Cipriano MJ, Kim PS, Fierro MA, et al. PfClpC Is an essential Clp chaperone required for plastid integrity and Clp protease stability in *Plasmodium falciparum*. Cell reports. 2017;21(7):1746–56. 29141210 10.1016/j.celrep.2017.10.081 PMC5726808

[72] Dahl EL, Rosenthal PJ. Multiple antibiotics exert delayed effects against the *Plasmodium falciparum* apicoplast. Antimicrob Agents Chemother. 2007;51(10):3485–90. 17698630 10.1128/AAC.00527-07 PMC2043295

[73] Hamilton MJ, Lee M, Le Roch KG. The ubiquitin system: an essential component to unlocking the secrets of malaria parasite biology. Mol Biosyst. 2014;10(4):715–23. doi: 10.1039/c3mb70506d 24481176 PMC3990246

[74] Okada M, Rajaram K, Swift RP, Mixon A, Maschek JA, Prigge ST, et al. Critical role for isoprenoids in apicoplast biogenesis by malaria parasites. eLife. 2022;11:e73208. doi: 10.7554/eLife.73208 35257658 PMC8959605

[75] Yeh E, DeRisi JL. Chemical rescue of malaria parasites lacking an apicoplast defines organelle function in blood-stage *Plasmodium falciparum*. PLOS Biology. 2011;9(8):e1001138. 21912516 10.1371/journal.pbio.1001138 PMC3166167

[76] Buchanan HD, Goodman CD, McFadden GI. Roles of the apicoplast across the life cycles of rodent and human malaria parasites. The Journal of eukaryotic microbiology. 2022;69(6):e12947. doi: 10.1111/jeu.12947 36070203 PMC9828729

[77] Kennedy K, Crisafulli EM, Ralph SA. Delayed death by plastid inhibition in apicomplexan parasites. Trends in Parasitology. 2019;35(10):747–59. doi: 10.1016/j.pt.2019.07.010 31427248

[78] Basseri S, Austin RC. Endoplasmic reticulum stress and lipid metabolism: mechanisms and therapeutic potential. Biochem Res Int. 2012;2012:841362. doi: 10.1155/2012/841362 22195283 PMC3238353

[79] Xu J, Taubert S. Beyond Proteostasis: Lipid metabolism as a new player in ER homeostasis. metabolites. 2021;11(1). doi: 10.3390/metabo11010052 33466824 PMC7830277

[80] Panagopoulos A, Taraviras S, Nishitani H, Lygerou Z. CRL4Cdt2: Coupling genome stability to ubiquitination. Trends in Cell Biology. 2020;30(4):290–302. doi: 10.1016/j.tcb.2020.01.005 32044173

[81] Nguyen L-T, Schmidt HA, von Haeseler A, Minh BQ. IQ-TREE: A fast and effective stochastic algorithm for estimating maximum-likelihood phylogenies. Molecular Biology and Evolution. 2014;32(1):268–74. doi: 10.1093/molbev/msu300 25371430 PMC4271533

[82] Minh BQ, Nguyen MAT, von Haeseler A. Ultrafast approximation for phylogenetic bootstrap. Molecular Biology and Evolution. 2013;30(5):1188–95. doi: 10.1093/molbev/mst024 23418397 PMC3670741

[83] Trager W, Jensen JB. Human malaria parasites in continuous culture. Science. 1976;193(4254):673–5. doi: 10.1126/science.781840 781840

[84] Lambros C, Vanderberg JP. Synchronization of *Plasmodium falciparum* erythrocytic stages in culture. The Journal of parasitology. 1979;65(3):418–20. 383936

[85] Sijwali PS, Rosenthal PJ. Functional evaluation of *Plasmodium* export signals in *Plasmodium berghei* suggests multiple modes of protein export. PLoS One. 2010;5(4):e10227. 20419102 10.1371/journal.pone.0010227 PMC2856681

[86] Sudhakar R, Das D, Thanumalayan S, Gorde S, Sijwali PS. *Plasmodium falciparum* Atg18 localizes to the food vacuole via interaction with the multi-drug resistance protein 1 and phosphatidylinositol 3-phosphate. The Biochemical journal. 2021;478(9):1705–32. 33843972 10.1042/BCJ20210001

[87] Govindarajalu G, Rizvi Z, Kumar D, Sijwali PS. Lyse-reseal erythrocytes for transfection of *Plasmodium falciparum*. Scientific Reports. 2019;9(1):19952. 31882761 10.1038/s41598-019-56513-9 PMC6934678

[88] Fidock DA, Wellems TE. Transformation with human dihydrofolate reductase renders malaria parasites insensitive to WR99210 but does not affect the intrinsic activity of proguanil. Proc Natl Acad Sci U S A. 1997;94(20):10931–6. doi: 10.1073/pnas.94.20.10931 9380737 PMC23535

[89] Batugedara G, Lu XM, Saraf A, Sardiu ME, Cort A, Abel S, et al. The chromatin bound proteome of the human malaria parasite. Microbial genomics. 2020;6(2). doi: 10.1099/mgen.0.000327 32017676 PMC7067212

[90] Tanneru N, Nivya MA, Adhikari N, Saxena K, Rizvi Z, Sudhakar R, et al. *Plasmodium* DDI1 is a potential therapeutic target and important chromatin-associated protein. bioRxiv. 2022:2021.10.29.466443. 10.1016/j.ijpara.2022.11.007 36657610

[91] Saliba KS, Jacobs-Lorena M. Production of *Plasmodium falciparum* gametocytes in vitro. Methods in molecular biology (Clifton, NJ). 2013;923:17–25. 10.1007/978-1-62703-026-7_2 PMC4672730 22990768

[92] Rudlaff RM, Kraemer S, Marshman J, Dvorin JD. Three-dimensional ultrastructure of *Plasmodium falciparum* throughout cytokinesis. PLOS Pathogens. 2020;16(6):e1008587. 32511279 10.1371/journal.ppat.1008587 PMC7302870

[93] Chaubey S, Grover M, Tatu U. Endoplasmic reticulum stress triggers gametocytogenesis in the malaria parasite. J Biol Chem. 2014;289(24):16662–74. doi: 10.1074/jbc.M114.551549 24755215 PMC4059112

