# Supplementary figures and images for: Plasmodium falciparum contains functional SCF and CRL4 ubiquitin E3 ligases, and CRL4 is critical for cell division and membrane integrity
Source: PLoS Pathog. 2024 Feb 28;20(2):e1012045. doi: 10.1371/journal.ppat.1012045 (PMC10927090; doi:10.1371/journal.ppat.1012045)

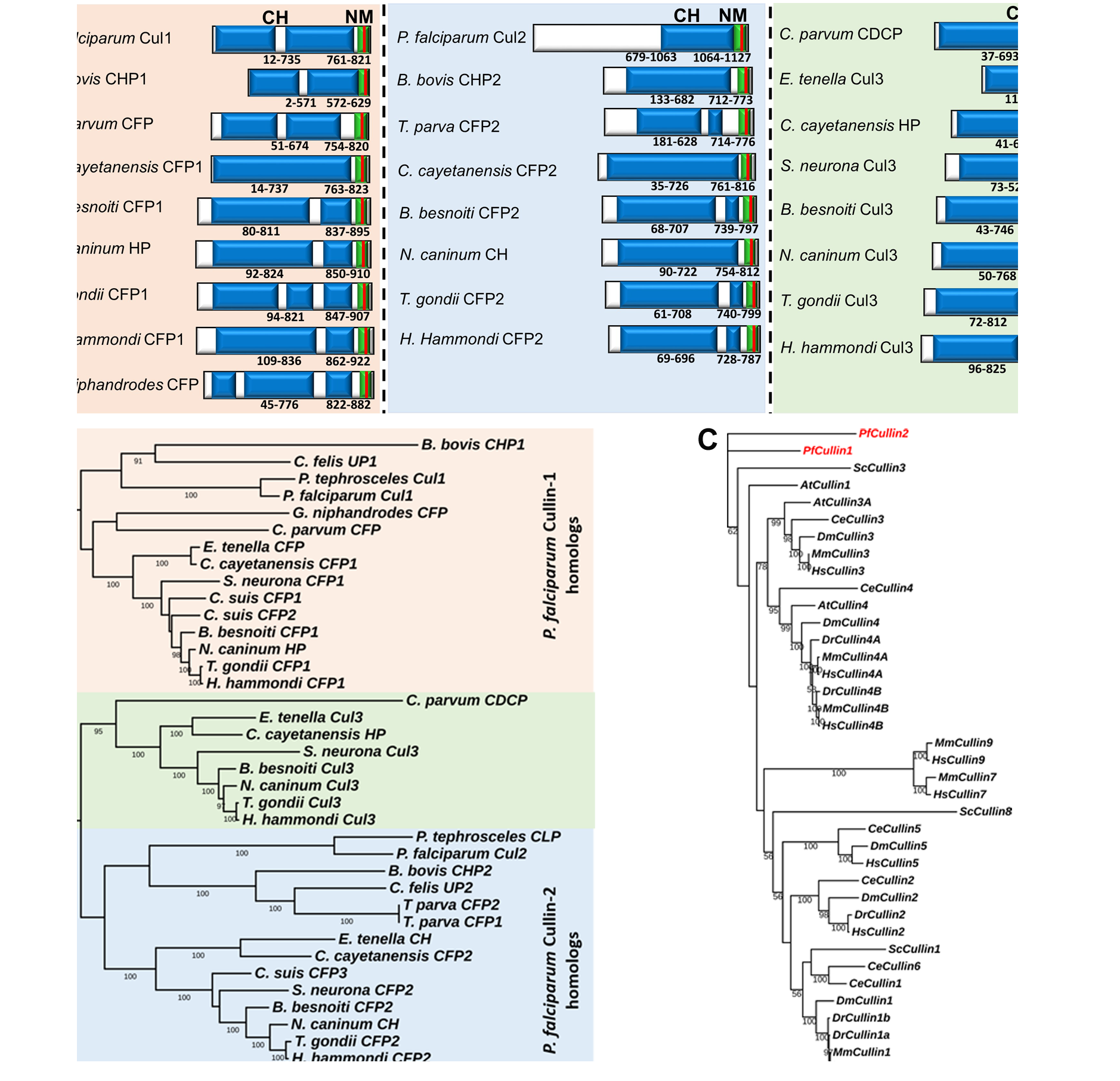

Supplement: S1 Fig — The amino acid sequences of apicomplexan cullin homologs were analysed for conserved domains and phylogenetic relatedness within apicomplexa and with homologs in model organisms. A. The schematic represents domain organization of the indicated cullins with the boundaries of cullin homology domain (CH) and neddylation motif (NM). The red bar in NM is the conserved putative lysine residue that is conjugated to NEDD8. B. The phylogram shows that PfCulin-1 (in pink) or PfCulin-2 (in blue) homologs of the indicated apicomplexan parasites cluster together on separate nodes. The cullins (in green) represent the third cullin homolog in the indicated parasites. The annotations are: CHP, conserved hypothetical protein; UP, unspecified product; CFP, cullin-family protein; HP, hypothetical protein; CDCP, cullin domain-containing protein; CLP, cullin like protein; CH, cullin homology domain; Cul, cullin. C. The phylogram of PfCullins and their homologs in model organisms shows that PfCullin-1 and PfCullin-2 are distantly related to their homologs in the indicated model organisms. The number on branches denote the strength of clustering (bootstrap value). (TIF) [file ppat.1012045.s001.tif]

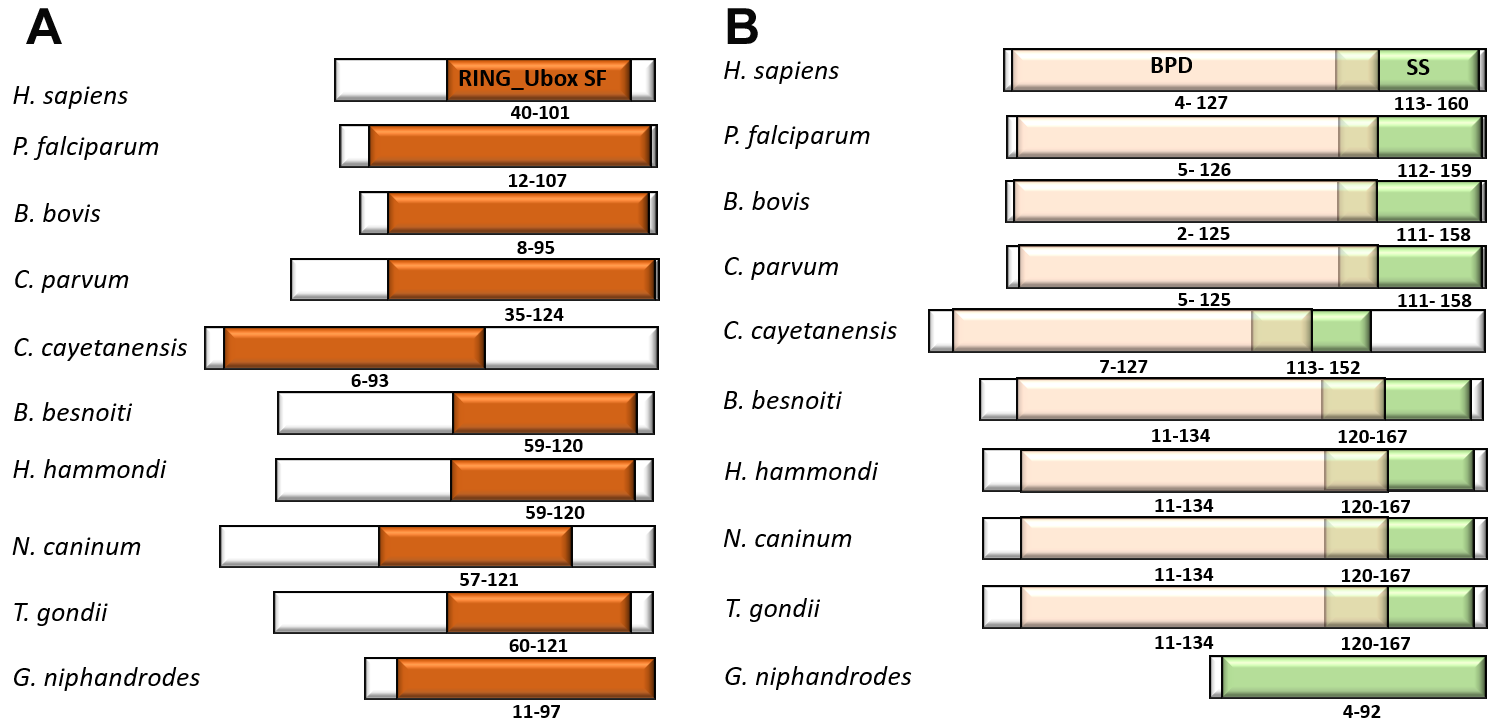

Supplement: S2 Fig — The amino acid sequences of putative apicomplexan Rbx1 and Skp1 were analysed for conserved domains and phylogenetic relatedness within apicomplexa. A. The schematic represents domain organization of the indicated Rbx1 proteins with the boundary of RING_Ubox superfamily (RING_Ubox SF) domain, which contains the consensus zinc finger motif [C-X2-C-X(9–39)-C-X(1–3)-H-X(2–3)-(N/C/H)-X2-C-X(4–48)-C-X2-C]. B. The schematic represents domain organization of the indicated Skp1 proteins with the boundaries of the BTB (Broad-Complex, Tramtrack and Bric a brac)/POZ (poxvirus and zinc finger) domain (BPD), Skp1 superfamily (SS) domain, and the sequence shared by BPD and SS regions. (TIF) [file ppat.1012045.s002.tif]

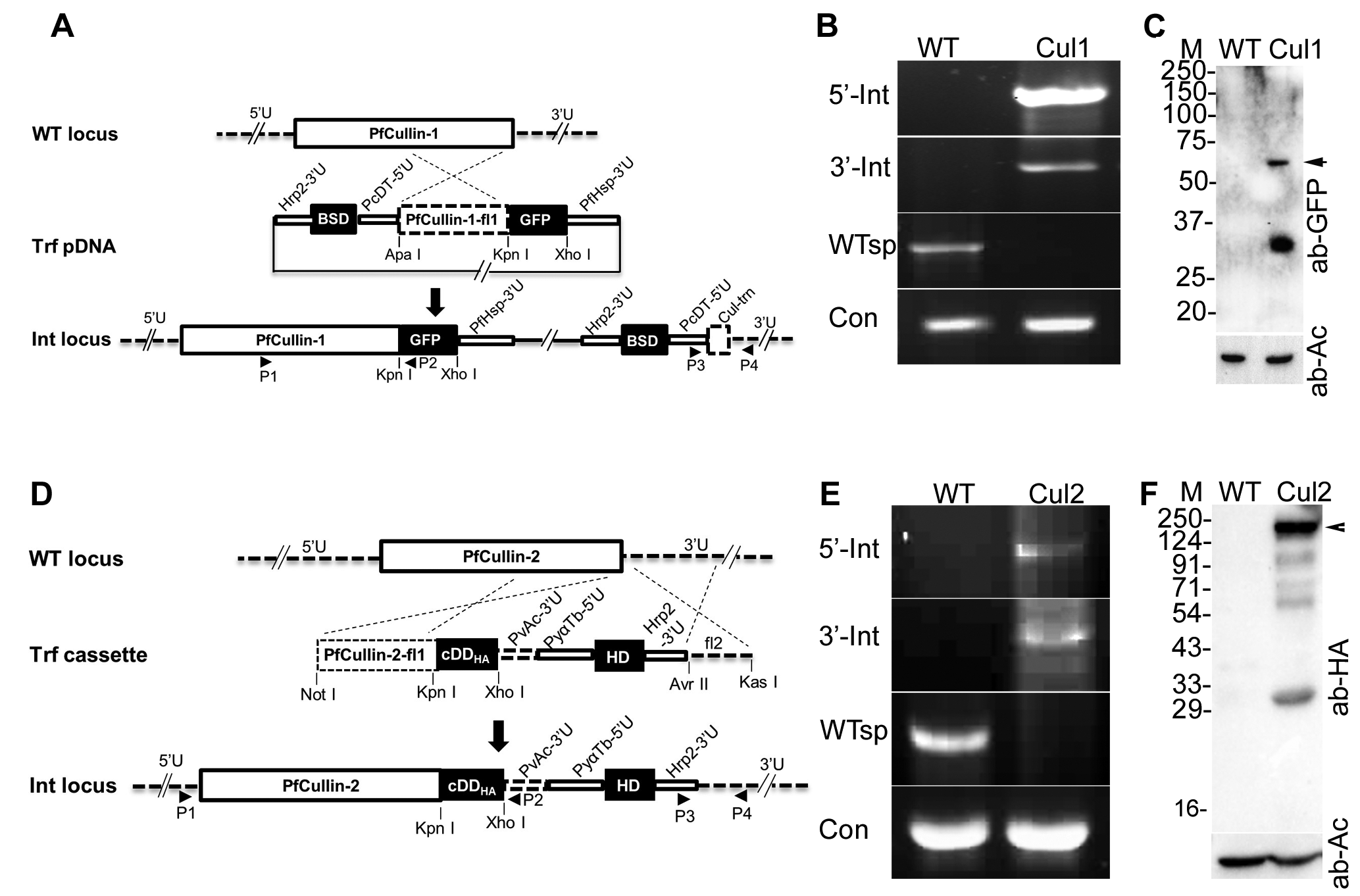

Supplement: S3 Fig — The endogenous PfCullin-1 and PfCullin-2 coding regions were replaced by PfCullin-1/GFP and PfCullin-2/cDDHA coding sequences in P. falciparum D10 strain, respectively. Cloned recombinant lines were assessed for integration of the desired DNA into the target locus by PCR and expression of the fusion protein by western blotting. A. The schematic represents integration of the transfection plasmid DNA (Trf pDNA) into the endogenous PfCullin-1 locus (WT locus), generating the integration locus (Int locus). Rectangular boxes represent coding regions for PfCullin-1, PfCullin-1-fl1, GFP and BSD. The untranslated regions of PfCullin-1 (5′U and 3′U), the location and orientation of primers (horizontal arrows), the restriction endonuclease sites (vertical lines), and regulatory regions in the linear transfection construct (PfHsp86-3′U, PcDT-5′U and PfHrp2-3′U) are indicated. B. The ethidium bromide-stained agarose gel shows PCR products amplified from the genomic DNAs of wild type (WT) and PfCul1GFPKI (Cul1) parasites using locus specific primers (P1 and P2 for 5’ integration (5’-Int), P3 and P4 for 3’ integration (3’-Int), P1 and P4 for wild type (WTsp), and primers specific for the PfRbx1 gene were used as a positive control (Con)). C. The lysates of wild type P. falciparum D10 (WT) and PfCul1GFPKI (Cul1) parasites were assessed for expression of PfCullin-1/GFP by western blotting using anti-GFP (ab-GFP) and anti-β-actin (ab-Ac) antibodies. The arrow indicates the size of truncated PfCullin-1/GFP, and the sizes of protein markers (M) are in kDa. D. The schematic represents integration of the linear transfection construct (Trf cassette) into the endogenous PfCullin-2 locus (WT locus), resulting into the generation of integration locus (Int locus). Rectangular boxes represent the coding regions for PfCullin-2, PfCullin-2-fl1, E. coli mutant DHFR with HA-tag (cDDHA) and human DHFR (HD). The flanking untranslated regions (5′U and 3′U), the location and orientation of primers (h [file ppat.1012045.s003.tif]

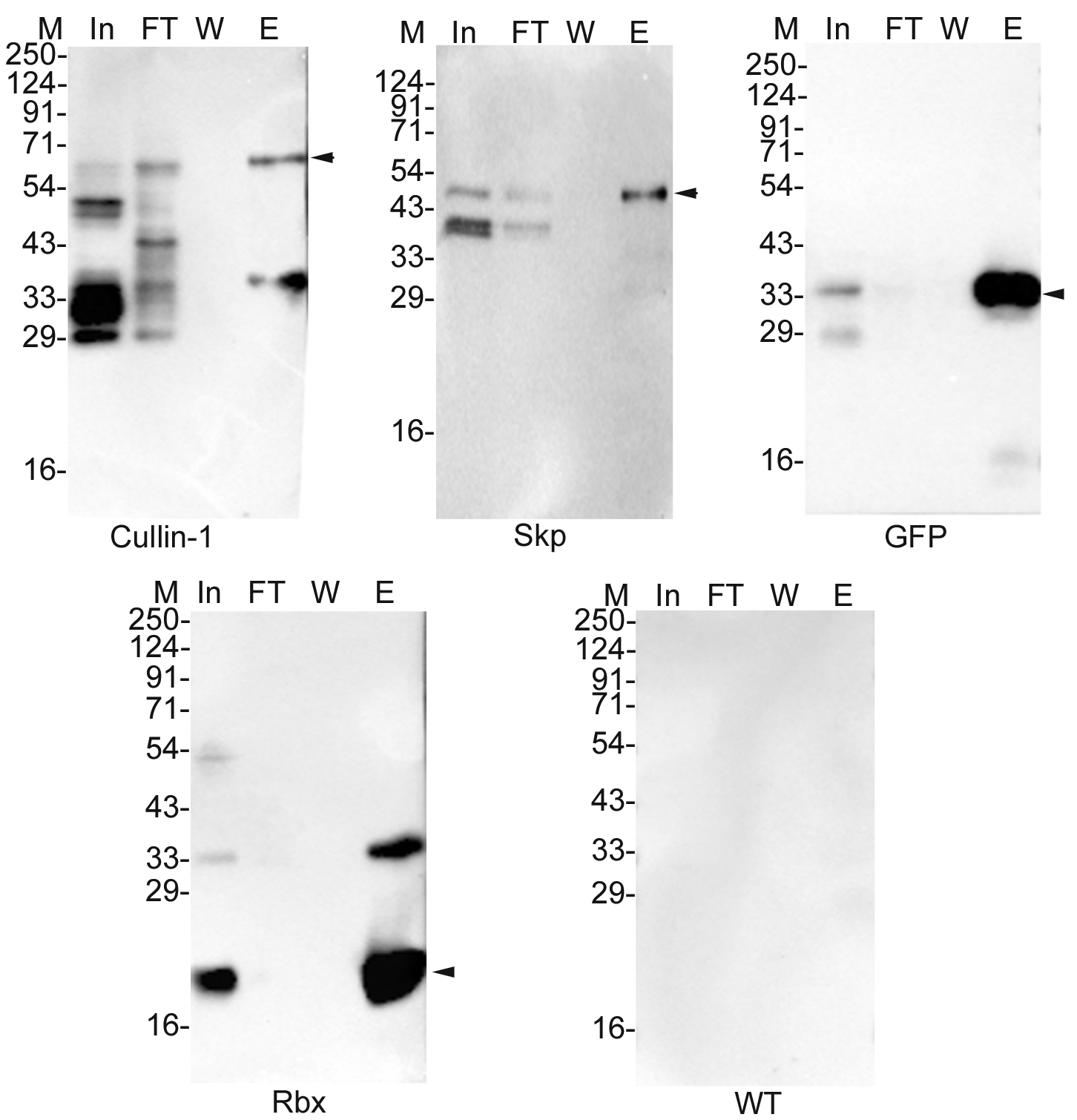

Supplement: S4 Fig — Lysates of PfCul1GFPKI (Cullin-1) PfSkp1/GFP-epi (Skp), and GFP-expressing control (GFP) parasites were processed for immunoprecipitation using GFP-Trap antibodies. Lysates of PfRbx1myc-epi (Rbx) and wild type P. falciparum D10 (WT) parasites were processed for immunoprecipitation using Myc-Trap antibodies. Aliquots of the input lysate (In), flow through (FT), wash (W), and eluate (E) for all the immunoprecipitations were assessed for the presence of target proteins using mouse anti-Myc (for Rbx and WT) or rabbit anti-GFP (for Cullin-1, Skp, GFP) antibodies by western blotting. Arrow heads indicate the target proteins, and sizes of protein markers (M) are in kDa. (TIF) [file ppat.1012045.s004.tif]

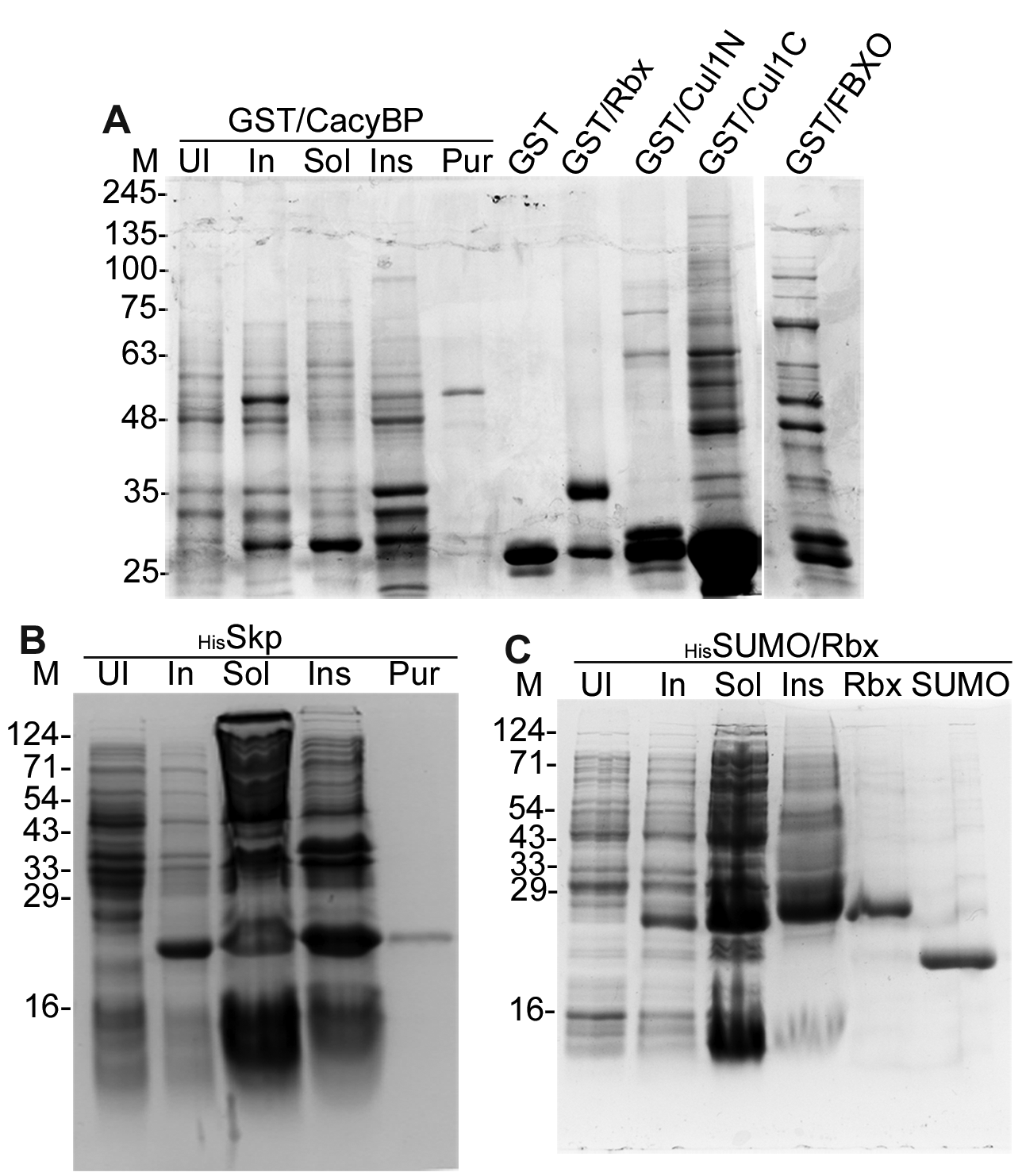

Supplement: S5 Fig — A. GST alone or GST-tagged PfCacyBP (GST/CacyBP), PfRbx1 (GST/Rbx), PfCullin-1 N-terminal (GST/Cul1N) and C-terminal (GST/Cul1C) fragments, and PfFBXO1 (GST/FBXO) were expressed in E. coli. The coomassie stained SDS-PAGE gel contains total lysates of un-induced (UI) and IPTG-induced (In) cells, soluble (Sol) and insoluble (Ins) fractions of the induced cells, and purified GST/CacyBP. The lanes GST, GST/Rbx, GST/Cul1N, GST/Cul1C and GST/FBXO contain the respective purified or enriched proteins. B. 6×His-tagged PfSkp1 (HisSkp) was expressed in E. coli cells. The coomassie stained SDS-PAGE gel contains total lysates of un-induced (UI) and IPTG-induced (In) cells, soluble (Sol) and insoluble (Ins) fractions of the induced cells, and purified protein (Pur). C. 6×His-SUMO-tagged PfRbx1 (HisSUMO/Rbx) or 6×His-SUMO (HisSUMO) proteins were expressed in E. coli. The coomassie stained SDS-PAGE gel contains total lysates of un-induced (UI) and IPTG-induced (In) cells, soluble (Sol) and insoluble (Ins) fractions of the induced cells, and purified HisSUMO/Rbx (Rbx) and HisSUMO (SUMO) proteins. The protein size markers (M) are in kDa. (TIF) [file ppat.1012045.s005.tif]

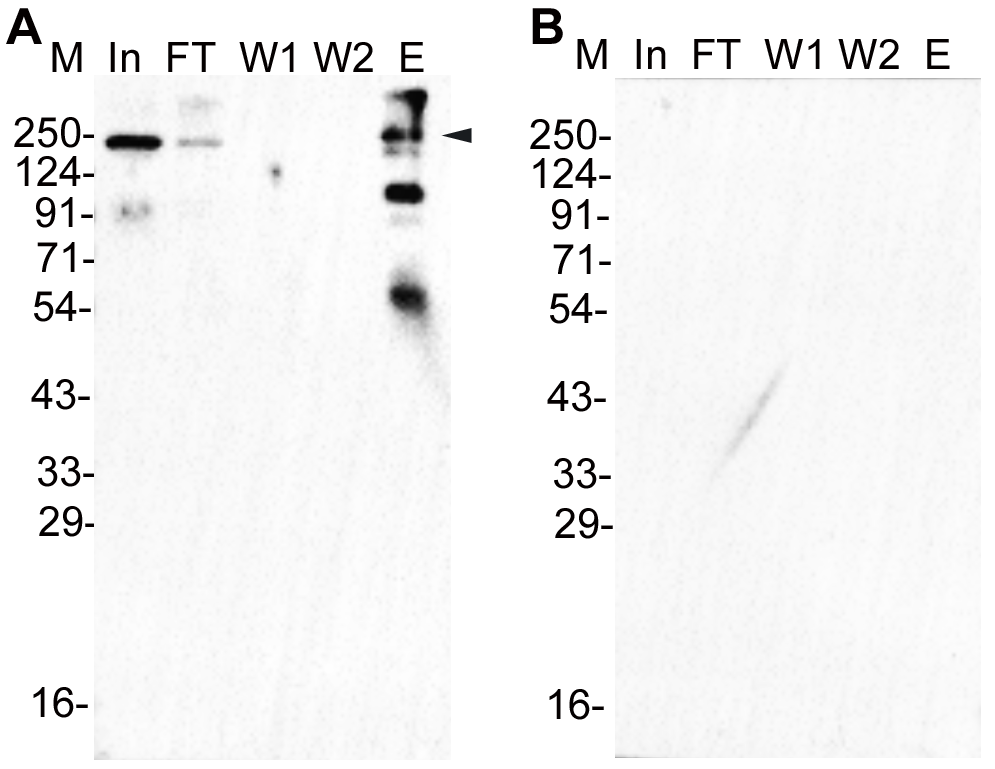

Supplement: S6 Fig — Lysates of PfCul2KDD10 (A) and wild type P. falciparum D10 (B) parasites were processed for immunoprecipitation using rabbit anti-HA antibodies. Aliquots of the parasite lysate inputs (In), flow through (FT), washes (W1, W2), and eluate (E) samples were probed with mouse anti-HA antibodies by western blotting. The sizes of protein markers (M) are in kDa. (TIF) [file ppat.1012045.s006.tif]

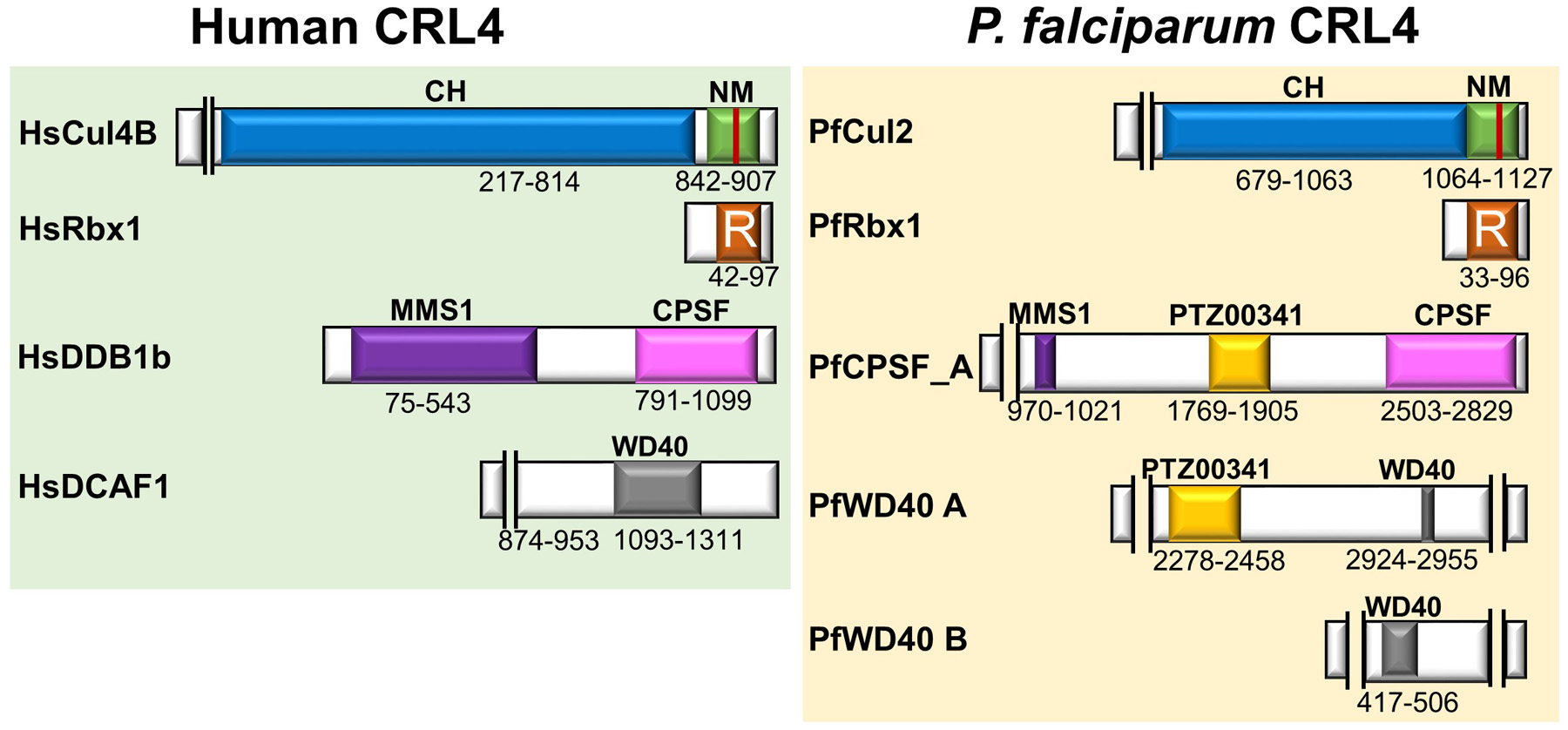

Supplement: S7 Fig — The schematic represents predicted domain architecture of the core subunits of human CRL4B and P. falciparum CRL4. The human CRL4B contains Cullin-4B (HsCul4B), Rbx1 (HsRbx1), DNA damage-specific binding protein 1b (HsDDB1b) and DDB1-Cullin-4-associated factor 1 (HsDCAF1). The P. falciparum CRL4 complex appears to contain Cullin-2 (PfCul2), Rbx1 (PfRbx1), cleavage and polyadenylation specificity factor subunit A (PfCPSF_A) and WD40 repeat proteins (PfWD40 A and PfWD40 B). The shaded boxes are conserved domains/motifs, which include cullin homology domain (CH) and neddylation motif (NM) in cullins, RING finger domain (R) in Rbx1, methyl methanesulfonate-sensitivity protein 1 (MMS1) and CPSF domains in HsDDB1 and PfCPSF_A proteins, WD40 repeat domain in HsDCAF1 and PfWD40 proteins. PfCPSF_A and PfWD40 A proteins also contain PTZ00341 domain of unknown function, which is present in ring-infected erythrocyte surface antigen (RESA) and several other Plasmodium proteins. The number below each domain corresponds to the number of boundary amino acid residues. The protein size is scaled to 1 cm for 100 aa; some regions are interrupted by two vertical lines to fit the protein size. (JPG) [file ppat.1012045.s007.jpg]

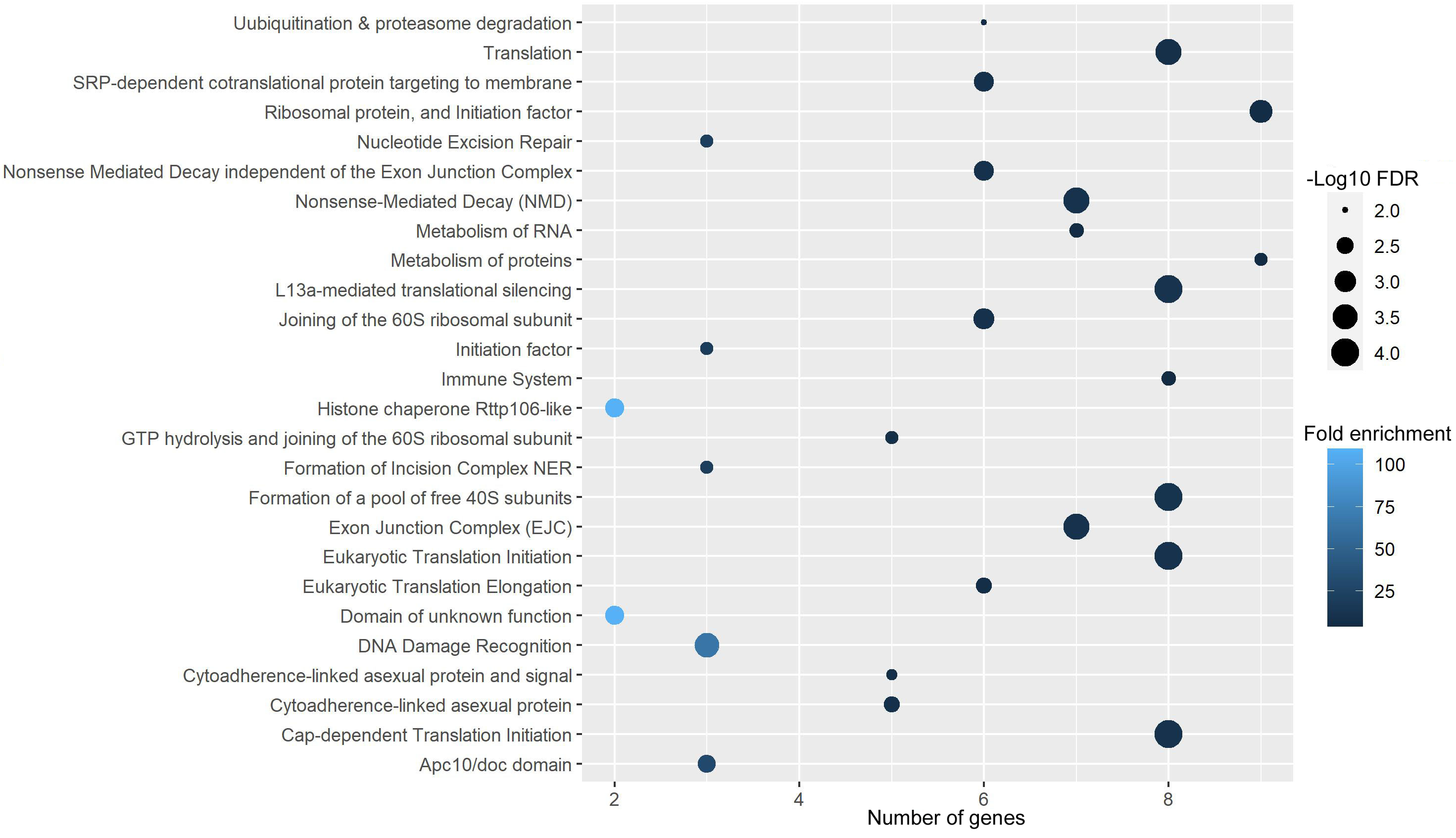

Supplement: S8 Fig — Uniport IDs of all the proteins exclusively present in the PfCullin-2 immunoprecipitate were used to generate biological pathways enrichment. The biological pathways with their fold enrichment and significance (-log10 of FDR) were plotted using the R programme. The plot shows fold enrichment (X-axis) of the pathways (Y-axis) with significant -Log10 FDR. (TIF) [file ppat.1012045.s008.tif]

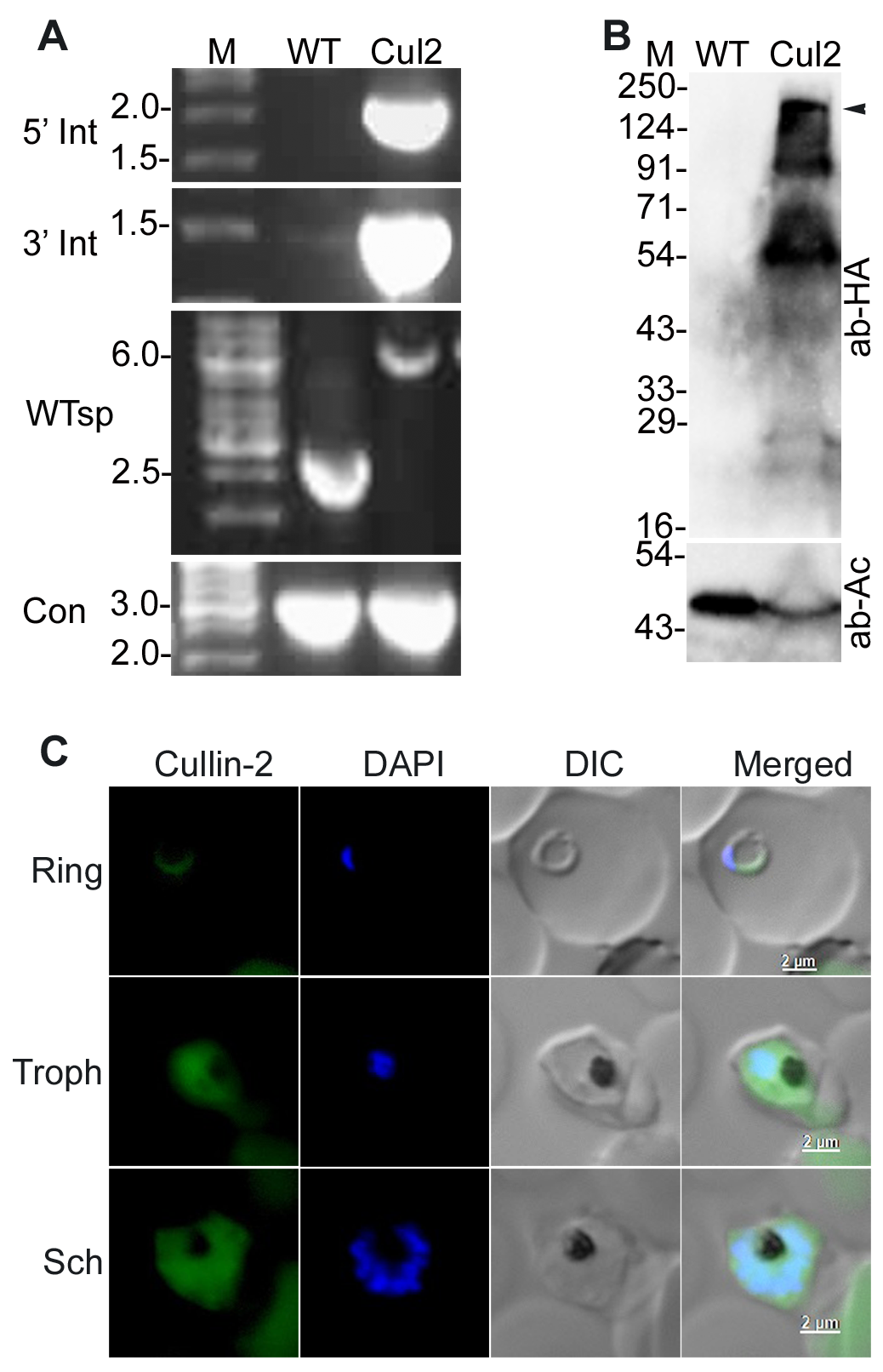

Supplement: S9 Fig — The endogenous PfCullin-2 coding region was replaced with PfCullin-2/cDDHA coding sequence as shown in SS3D Fig. A cloned line was assessed for the presence of integration locus by PCR and expression of PfCullin-2/cDDHA protein by western blotting. A. The ethidium bromide-stained agarose gel shows PCR products amplified from the wild type (WT) and PfCul2KD3D7 (Cul2) genomic DNAs using the indicated primer sets. DNA size markers (M) are in kbp. B. The lysates of wild type P. falciparum 3D7 (WT) and PfCul2KD3D7 (Cul2) parasites were assessed for expression of PfCullin-2/cDDHA by western blotting using anti-HA (ab-HA) and anti-β-actin (ab-Ac) antibodies. The arrow indicates the size of full-length PfCullin-2/cDDHA. C. Ring, trophozoite (Troph), and schizont (Sch) stages of PfCul2KD3D7 parasites were assessed for localization of PfCullin-2/cDDHA by IFA using anti-HA antibodies. The images are for PfCullin-2/cDDHA signal (Cullin-2), nuclear staining (DAPI), parasite and RBC boundaries (DIC), and overlap of all the three images (Merged). Scale bar is in the merged image. (TIF) [file ppat.1012045.s009.tif]

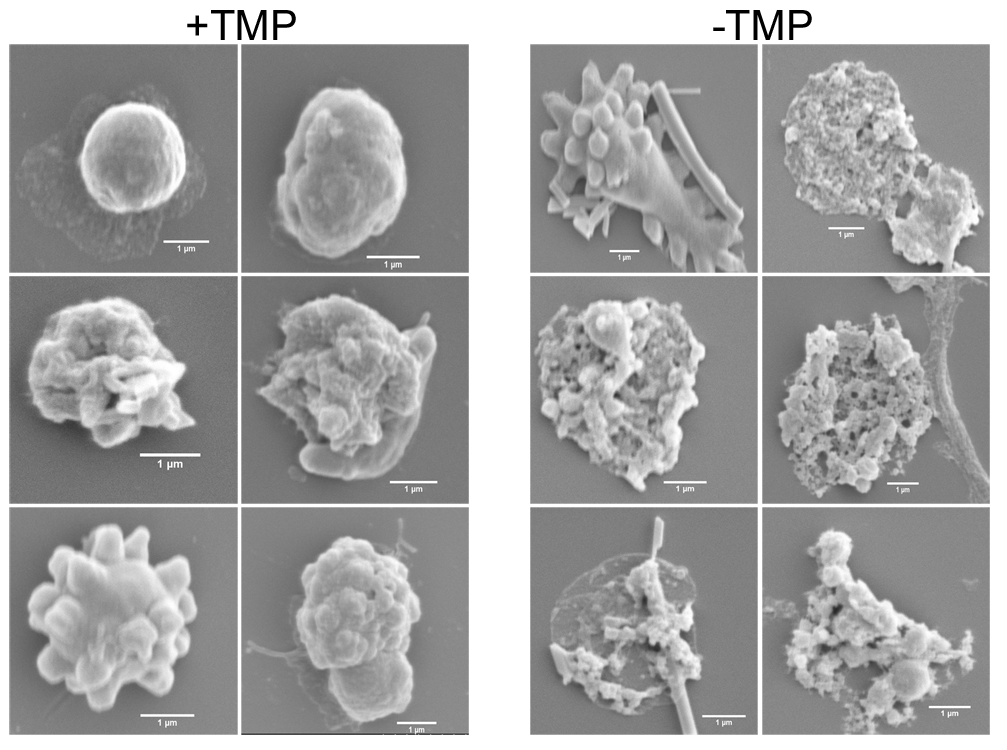

Supplement: S10 Fig — PfCul2KD3D7 parasites were cultured with (+TMP) or without (-TMP) for three cycles. The 3rd cycle parasites were isolated by saponin lysis and processed for SEM. The images show outer surface of the parasites, which appears smooth in case of +TMP, but distorted in case of -TMP parasites. (TIF) [file ppat.1012045.s010.tif]

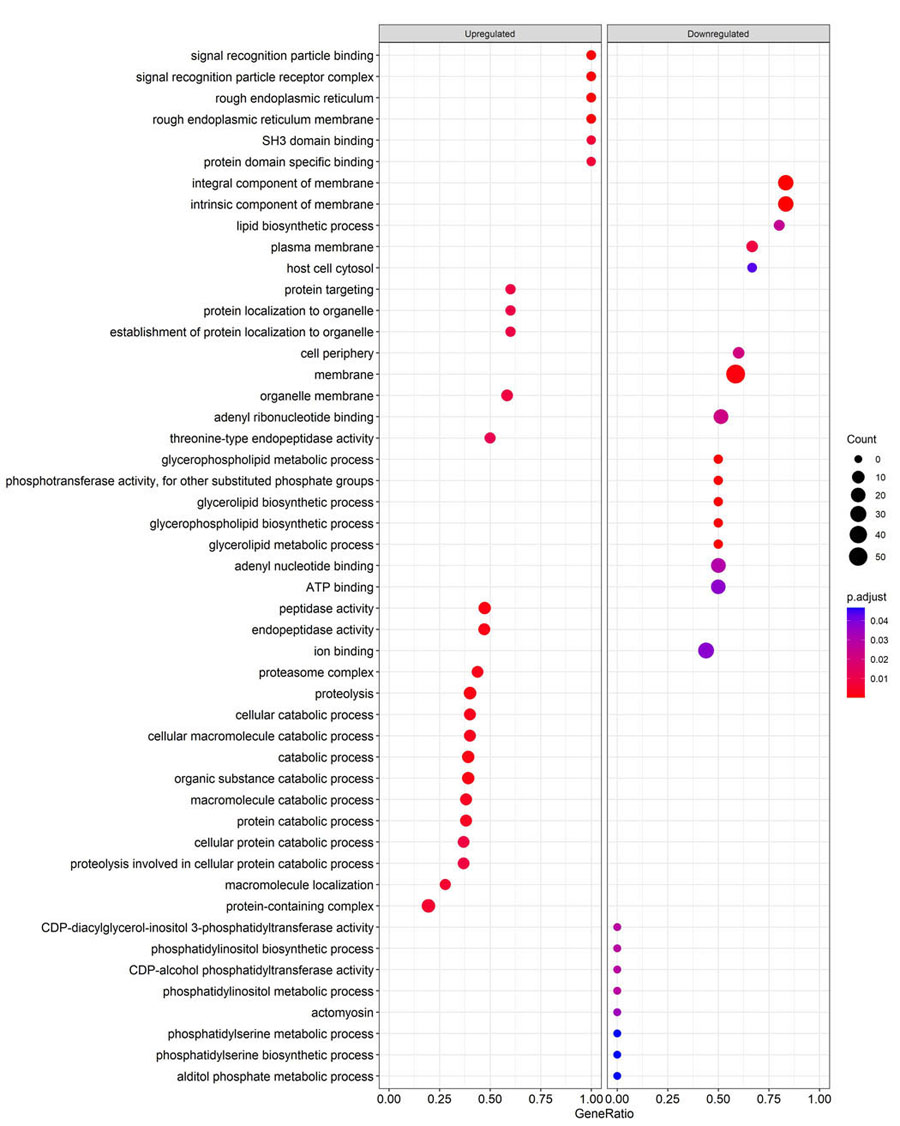

Supplement: S11 Fig — PfCul2KD3D7 parasites were cultured with or without trimethoprim for three cycles, and the 3rd cycle trophozoites were processed for LFQ. Differentially regulated proteins were used for gene set enrichment analysis (GSEA) using the cluster profiler for gene ontology (GO) term. The plot shows biological pathways on Y-axis and gene enrichment values on X-axis. The size and color of the circle denotes the number of GO terms involved in a particular pathway and their confidence, respectively. Bigger the size and darker the red color of the circle corresponds to higher confidence and enrichment of the pathways. (JPG) [file ppat.1012045.s011.jpg]

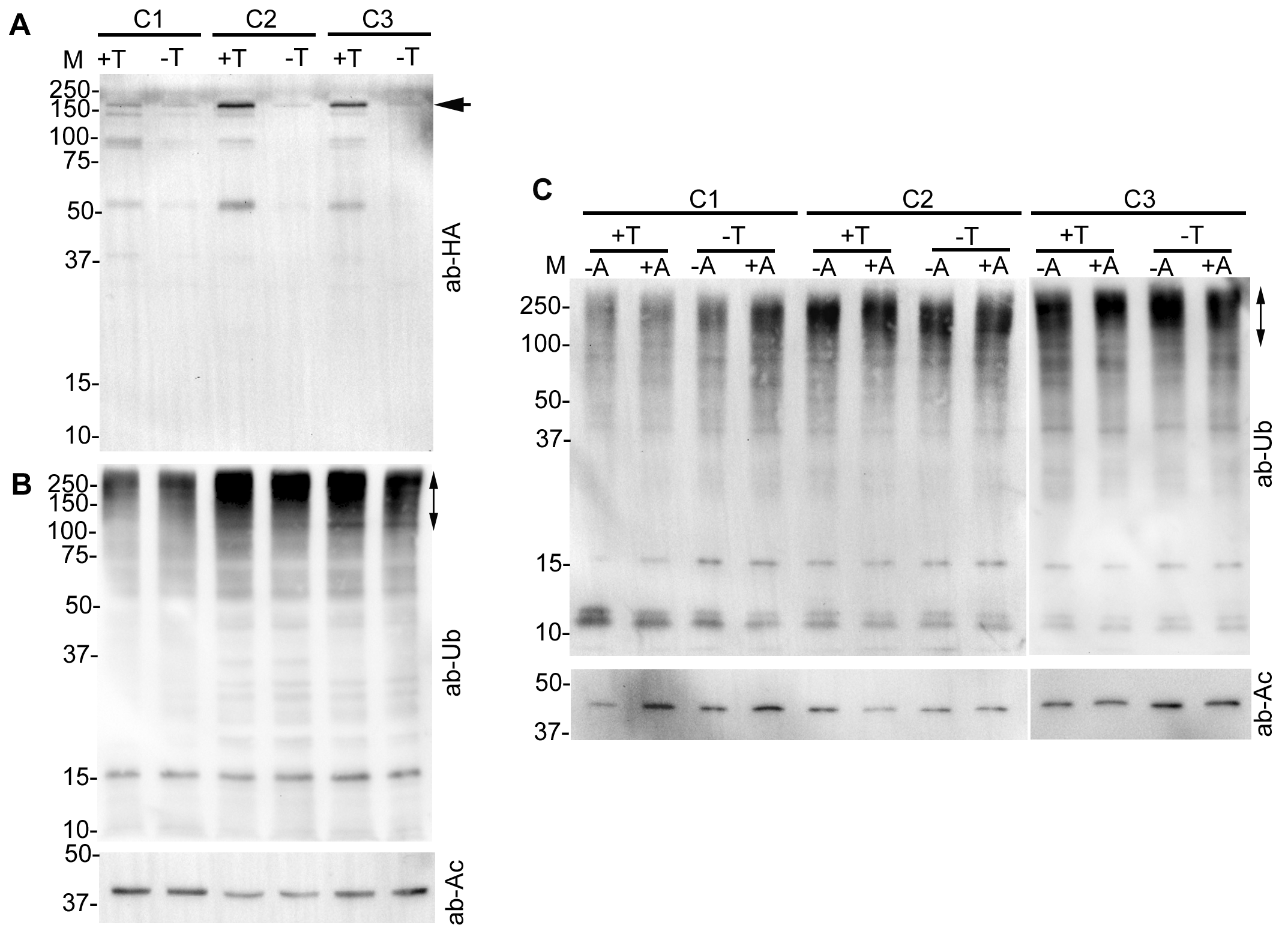

Supplement: S12 Fig — Synchronized PfCull2KD3D7 parasites were grown with (+T) or without (-T) trimethoprim for three consecutive cycles (C1, C2 and C3), harvested at the trophozoite stage in each cycle, and processed for PfCullin-2 levels (A), overall ubiquitination profiles (B), and ubiquitination activity (C). A. The indicated whole parasite lysates were checked for PfCullin-2 levels (indicated with arrow) using anti-HA antibodies (ab-HA). B. The indicated whole parasite lysates were checked for overall ubiquitination profile (indicated with a vertical arrow) using anti-ubiquitin antibodies (ab-Ub). The blot in B was stripped and probed for β-actin levels as a loading control (ab-Ac). C. The indicated parasite extracts were assessed for ubiquitination activity without (-A) or with (+A) ATP, and the reactions were processed for ubiquitinated protein levels by western blot using anti-ubiquitin antibodies (ab-Ub). The ubiquitinated proteins are indicated with a vertical arrow. The blots were stripped and probed for β-actin levels as a loading control (ab-Ac). The sizes of protein markers (M) are in kDa. (TIF) [file ppat.1012045.s012.tif]
