# Supplementary material for: Plasmodium falciparum contains functional SCF and CRL4 ubiquitin E3 ligases, and CRL4 is critical for cell division and membrane integrity
Source: PLoS Pathog. 2024 Feb 28;20(2):e1012045. doi: 10.1371/journal.ppat.1012045 (PMC10927090; doi:10.1371/journal.ppat.1012045)
Supplement: S1 Table — PfCullin-1, PfCullin-2, PfRbx1 and PfSkp1 homologs in reference apicomplexan parasite strains of different families. (DOCX) [file ppat.1012045.s013.docx]

**S1 Table. Putative CRL subunits in apicomplexan parasites.** PfCullin-1, PfCullin-2, PfRbx1 and PfSkp1 homologs in reference apicomplexan parasite strains of different families.

| **Family** | **Parasite** | **Cullin homologs** | **% Identity with PfCullin-1** | **% Identity with PfCullin-2** | **% Identity with the closest to human cullin** | **PfRbx1 homologs** | **PfSkp1**  **homologs** |
| --- | --- | --- | --- | --- | --- | --- | --- |
| Plasmodiidae | *Hepatocystis sp* | HEP_00189600 (cullin-1, putative) | 66.5% | 22.4% | Cullin-3 (24.3%) | HEP_00025600(E3 ubiquitin-protein ligase RBX1, putative) | HEP_00355900 (suppressor of kinetochore protein 1, putative) |
|  |  | HEP_00066600 (cullin-like protein, putative) | 20.7% | 41.9% | Cullin-4B (20.7%) |  |  |
| Babesiidae | *Babesia bovis* T2Bo | BBOV_III006880, (conserved hypothetical protein) | 22.8% | 18.5% | Cullin-4B (22.4%) | BBOV_III007590 (hypothetical protein) | BBOV_IV006860 (cytosolic glycoprotein FP21, putative) |
|  |  | BBOV_III001760, (conserved hypothetical protein) | 17.5% | 18.7% | Cullin-4B (21.7%) |  |  |
| Theileriidae | *Cytauxzoon felis* Winnie | CF000930  (unspecified product) | 26.7% | 20.2% | Cullin-1 (21.0%) | CF002561 (unspecified product) | CF000801 (unspecified product) |
|  |  | CF001230, (unspecified product) | 18.4% | 17.7% | Cullin-4B (18.8%) |  |  |
|  | *Theileria parva* Muguga | TpMuguga_04g0059 (cullin family protein) | 21.9% | 22.4% | Cullin-1 (20.4%) | TpMuguga_04g00669  (RING-box protein 1B) | TpMuguga_03g00225  (dimerization domain protein) |
|  |  | TpMuguga_03g02140 (cullin family protein) | 20.1% | 18.5% | Cullin-4B (19.4%) |  |  |
| Cryptosporidiidae | *Cryptosporidium parvum* Iowa II | cgd4_3150 (cullin family protein) | 26.0% | 20.0% | Cullin-4B (24.0%) | cgd8_930  (Zinc finger, RING/FYVE/PHD-type domain containing protein) | cgd7_2500  (Skp1 family protein) |
|  |  | cgd3_2790 (cullin domain containing protein) | 18.3% | 20.4% | Cullin-3 (22.2%) |  |  |
| Eimeriidae | *Cyclospora cayetanensis* CHN_HEN01 | cyc_05965 (cullin family protein) | 26.8% | 17.6% | Cullin-4B (30.7%) | cyc_05145 (anaphase promoting complex subunit related protein) | cyc_03497  (s-phase kinase-associated) |
|  |  | cyc_01443 (hypothetical protein) | 19.6% | 17.6% | Cullin-3 (34.7%) |  |  |
|  |  | cyc_06635 (cullin family protein) | 19.5% | 18.3% | Cullin-4B (267.0%) |  |  |
|  | *Eimeria tenella* Houghton | ETH_00004725  **(**cullin family protein**)** | 27.3% | 18.3% | Cullin-4B (31.9%) | ETH_00017775 (Anaphase promoting complex subunit 11, related) | ETH_00009000  (S-phase kinase-associated protein, putative) |
|  |  | ETH_00015050  **(**cullin family protein**)** | 22.6% | 22.0% | Cullin-3 (35.8%) |  |  |
|  |  | ETH_00020865  **(**cullin family protein**)** | 21.7% | 23.2% | Cullin-4B (35.4%) |  |  |
| Sarcocystidae | *Besnoitia besnoiti* Bb-Ger1 | BESB_020620 (cullin family protein) | 27.4% | 18.6% | Cullin-4B (31.5%) | BESB_066840 (ring box protein 1 family protein) | BESB_022380 (putative suppressor of kinetochore protein 1) |
|  |  | BESB_078110(cullin family protein) | 18.1% | 21.1% | Cullin-4B (32.6%) |  |  |
|  |  | BESB_013740 (putative cullin 3) | 20.3% | 16.1% | Cullin-3 (36.0%) |  |  |
|  | *Cystoisospora suis* Wien I | CSUI_004856(cullin family) | 22.4% | 13.3% | Cullin-4A (23.1%) | CSUI_007688  (ring box protein 1 family protein) | CSUI_000005 (suppressor of kinetochore protein 1) |
|  |  | CSUI_001492(cullin family) | 27.8% | 18.8% | Cullin-3 (35.0%) |  |  |
|  |  | CSUI_004490(cullin family protein) | 22.1% | 22.9% | Cullin-4B (43.3%) |  |  |
|  | *Hammondia hammondi* H.H.34 | HHA_289310(cullin family protein) | 28.2% | 17.6% | Cullin-4B (33.07%) | HHA_213690  (ring box protein 1 family protein) | HHA_207680 (suppressor of kinetochore protein 1, putative) |
|  |  | HHA_316660(cullin family protein) | 16.9% | 18.0% | Cullin-4B (32.7%) |  |  |
|  |  | HHA_201770(cullin 3, putative) | 19.2% | 16.0% | Cullin-3 (36.7%) |  |  |
|  | *Neospora caninum* Liverpool | NCLIV_041660(hypothetical protein) | 28.4% | 18.1% | Cullin-4B (32.3%) | NCLIV_038660 (conserved hypothetical protein) | NCLIV_002630 (protein F46A9.4, confirmed by transcript evidence, related) |
|  |  | NCLIV_059100(putative cullin homolog) | 19.1% | 20.2% | Cullin-4B (32.5%) |  |  |
|  |  | NCLIV_022980(hypothetical protein) | 19.8% | 17.4% | Cullin-3 (36.1%) |  |  |
|  | *Sarcocystis neurona* SN3 | SN3_0430007(cullin family protein) | 21.1% | 13.0% | Cullin-1 (23.33%) | SN3_00600825 (ring-box protein variant) | SN3_00102155 (glycoprotein fp21) |
|  |  | SN3_00900770(cullin 3) | 15.2% | 13.7% | Cullin-3 (32.0%) |  |  |
|  |  | SN3_01900570(cullin family protein) | 20.8% | 20.1% | Cullin-4B (33.1%) |  |  |
|  | *Toxoplasma gondii* ME49 | TGME49_289310 (cullin family protein) | 27.8% | 17.4% | Cullin-4B (31.8%) | TGME49_213690 (ring box protein 1 family protein) | TGME49_207680 (suppressor of kinetochore protein 1, putative) |
|  |  | TGME49_316660 (cullin family protein) | 18.1% | 18.9% | Cullin-4B (33.2%) |  |  |
|  |  | TGME49_201770(cullin 3, putative) | 18.8% | 15.8% | Cullin-3 (36.8%) |  |  |
| Gregarinidae | *Gregarina niphandrodes* | GNI_128180 (cullin family protein) | 14.5% | 17.4% | Cullin-4B (25.0%) | GNI_136150 (putative ring box protein 1) | GNI_136590 (putative Skp1 family protein) |
