# Supplementary material for: Plasmodium falciparum contains functional SCF and CRL4 ubiquitin E3 ligases, and CRL4 is critical for cell division and membrane integrity
Source: PLoS Pathog. 2024 Feb 28;20(2):e1012045. doi: 10.1371/journal.ppat.1012045 (PMC10927090; doi:10.1371/journal.ppat.1012045)
Supplement: S2 Table — Shown are the Uniprot (PlasmoDB) ID, score (Sc), coverage (Co) and unique peptides (UP) for each protein identified in three independent biological repeats. (DOCX) [file ppat.1012045.s014.docx]

**S2 Table.** **Proteins identified in the PfCullin1/GFP immunoprecipitate.** Shown are the Uniprot (PlasmoDB) ID, score (Sc), coverage (Co) and unique peptides (UP) for each protein identified in three independent biological repeats.

| **ID** | **Protein** | **Experiment 1** | | | **Experiment 2** | | | **Experiment 3** | | |
| --- | --- | --- | --- | --- | --- | --- | --- | --- | --- | --- |
|  |  | **Sco** | **Cov** | **UP** | **Sco** | **Cov** | **UP** | **Sco** | **Cov** | **UP** |
| Q8IAU5  (PF3D7_0811000) | Cullin-1, putative | 35.98 | 24.13 | 10 | 11.42 | 18.09 | 3 | 143.44 | 22.68 | 14 |
| C6KTB1  (PF3D7_0627500) | Protein DJ-1 | 6.79 | 33.86 | 3 | 32.42 | 43.39 | 5 | 13.42 | 39.68 | 5 |
| O77367  (PF3D7_0319100) | E3 ubiquitin-protein ligase RBX1, putative | 53.13 | 50.43 | 10 | 82.92 | 41.26 | 9 | - | - | - |
| Q8IHR4  (PF3D7_1145400) | Dynamin-like protein | 11.05 | 25.09 | 3 | 4.91 | 20.67 | 2 | - | - | - |
| Q8IBZ9  (PF3D7_0709000) | Putative chloroquine resistance transporter | 2.45 | 25.71 | 1 | 4.15 | 17.45 | 1 | - | - | - |
| Q8IC05  (PF3D7_0708400) | Heat shock protein 90 | 24.18 | 24.83 | 7 | - | - | - | 154.94 | 36.11 | 26 |
| Q8IJN7  (PF3D7_1015900) | Enolase | 19.14 | 28.48 | 5 | - | - | - | 74.03 | 41.48 | 12 |
| Q7KQL9  (PF3D7_1444800) | Fructose-bisphosphate aldolase | 11.82 | 16.53 | 4 | - | - | - | 53.69 | 44.72 | 13 |
| Q8IKW5  (PF3D7_1451100) | Elongation factor 2 | 11.25 | 22.96 | 3 | - | - | - | 41.60 | 23.32 | 12 |
| Q8IJD4  (PF3D7_1026800) | 40S ribosomal protein S2 | 8.70 | 17.49 | 1 | - | - | - | 11.66 | 27.38 | 3 |
| Q8IBV7  (PF3D7_0714000) | Histone H2B variant | 8.19 | 43.90 | 1 | - | - | - | 2.52 | 41.46 | 7 |
| Q8IDQ9  (PF3D7_1343000) | Phosphoethanolamine N-methyltransferase | 5.54 | 16.17 | 1 | - | - | - | 51.61 | 41.73 | 8 |
| P27362  (PF3D7_0922500) | Phosphoglycerate kinase | 5.14 | 8.89 | 2 | - | - | - | 52.75 | 52.40 | 22 |
| Q8IIG6  (PF3D7_1120100) | Phosphoglycerate mutase, putative | 4.63 | 34.40 | 1 | - | - | - | 21.68 | 42.80 | 5 |
| Q76NM3  (PF3D7_1324900) | L-lactate dehydrogenase | 4.48 | 18.35 | 1 | - | - | - | 67.98 | 40.19 | 14 |
| Q8I0V4  (PF3D7_1222300) | Endoplasmin, putative | 4.32 | 12.91 | 2 | - | - | - | 23.03 | 22.17 | 7 |
| Q8IEU2  (PF3D7_1302100) | Gamete antigen 27/25 | 4.07 | 3.69 | 1 | - | - | - | 26.19 | 36.87 | 7 |
| Q8ILL2  (PF3D7_1424400) | 60S ribosomal protein L7-3, putative | 4.06 | 38.16 | 2 | - | - | - | 9.21 | 21.91 | 3 |
| Q8IKF0  (PF3D7_1468700) | Eukaryotic initiation factor 4A | 3.87 | 14.82 | 2 | - | - | - | 55.45 | 30.65 | 10 |
| Q8IM10  (PF3D7_1408600) | 40S ribosomal protein S8e, putative | 3.10 | 10.09 | 1 | - | - | - | 13.95 | 35.78 | 3 |
| Q8IBD4  (PF3D7_0830800) | surface-associated interspersed protein 8.2 (SURFIN 8.2) | 2.19 | 16.98 | 1 | - | - | - | 2.84 | 12.74 | 1 |
| Q8II72  (PF3D7_1129100) | Parasitophorous vacuolar protein 1 | 1.89 | 17.70 | 1 | - | - | - | 1.64 | 10.18 | 1 |
| P50250  (PF3D7_0520900) | Adenosylhomocysteinase | - | - | - | - | - | - | 24.85 | 28.81 | 5 |
| Q8IM03  (PF3D7_1409300) | DNA damage-inducible protein 1 | - | - | - | 89.17 | 39.01 | 13 | 2.01 | 19.11 | 2 |
| Q8I207  (PF3D7_0401800) | *Plasmodium* exported protein (PHISTb) | - | - | - | 31.21 | 32.32 | 8 | 6.54 | 17.32 | 1 |
| O77313  (PF3D7_0303000) | N-ethylmaleimide-sensitive fusion protein | - | - | - | 6.75 | 16.73 | 1 | 1.84 | 9.83 | 1 |
| Q8IAN4  (PF3D7_0803400) | DNA repair and recombination protein RAD54, putative | - | - | - | 1.63 | 10.33 | 1 | - | - | - |
| Q7KQJ9  (PF3D7_1226600) | Proliferating cell nuclear antigen 2 | - | - | - | - | - | - | 3.93 | 15.53 | 1 |
| Q8IEI4  (PF3D7_1313000) | Ubiquitin-like protein NEDD8 | - | - | - | - | - | - | 8.86 | 77.63 | 2 |
| Q8I608  (PF3D7_0919000) | Nucleosome assembly protein | - | - | - | - | - | - | 3.91 | 21.61 | 2 |
| Q8IL07  (PF3D7_1446600) | Centrin-2 | - | - | - | - | - | - | 3.20 | 28.57 | 1 |
| O97225  (PF3D7_0303500) | Spindle pole body protein, putative | - | - | - | - | - | - | 2.88 | 13.03 | 1 |
