# Supplementary material for: Plasmodium falciparum contains functional SCF and CRL4 ubiquitin E3 ligases, and CRL4 is critical for cell division and membrane integrity
Source: PLoS Pathog. 2024 Feb 28;20(2):e1012045. doi: 10.1371/journal.ppat.1012045 (PMC10927090; doi:10.1371/journal.ppat.1012045)
Supplement: S3 Table — Shown are the Uniprot (PlasmoDB) ID, score (Sc), coverage (Co) and unique peptides (UP) for proteins present in three independent biological repeats. (DOCX) [file ppat.1012045.s015.docx]

**S3 Table.** **Proteins identified in the PfRbx1myc immunoprecipitate.** Shown are the Uniprot (PlasmoDB) ID, score (Sc), coverage (Co) and unique peptides (UP) for proteins present in three independent biological repeats.

| **ID** | **Protein** | **Experiment 1** | | | **Experiment 2** | | | **Experiment 3** | | |
| --- | --- | --- | --- | --- | --- | --- | --- | --- | --- | --- |
|  |  | **Sco** | **Cov** | **UP** | **Sco** | **Cov** | **UP** | **Sco** | **Cov** | **UP** |
| O77367  (PF3D7_0319100) | E3 ubiquitin-protein ligase RBX1, putative | 10.9 | 19.6 | 1 | 40.7 | 55.1 | 4 | 52.16 | 47.6 | 4 |
| Q8IAU5  (PF3D7_0811000) | Cullin-1, putative | 20.8 | 25.4 | 11 | 104.0 | 48.6 | 31 | 99.79 | 38.0 | 28 |
| Q8I259  (PF3D7_0108300) | Conserved Plasmodium protein, unknown function | 36.34 | 14.00 | 8 | 4.18 | 19.41 | 2 | - | - | - |
| Q8I1Z9  (PF3D7_0403100) | Regulator of chromosome condensation, putative | 20.9 | 28.3 | 6 | 14.6 | 21.7 | 3 | 9.16 | 19.2 | 1 |
| Q8IAU7  (PF3D7_0811200) | ER membrane protein complex subunit 1, putative | 19.03 | 16.77 | 7 | 9.54 | 22.51 | 4 | 6.21 | 15.18 | 1 |
| C6KT19  (PF3D7_0617900) | Histone H3 variant | 14.37 | 24.26 | 1 | 12.56 | 39.71 | 2 | 11.49 | 29.41 | 1 |
| Q8I562  (PF3D7_1236100) | Clustered-asparagine-rich protein | 12.71 | 11.46 | 3 | 4.11 | 7.87 | 1 | 5.46 | 13.26 | 2 |
| Q8IJ60  (PF3D7_1034900) | Methionine-tRNA ligase | 11.62 | 17.66 | 3 | 2.14 | 13.72 | 2 | 17.05 | 22.61 | 7 |
| Q8I2W3  (PF3D7_0919000) | Nucleosome assembly protein | 9.1 | 22.3 | 4 | 16.6 | 23.9 | 6 | 20.22 | 30.0 | 7 |
| C6KT82  (PF3D7_0624600) | ISWI chromatin-remodelling complex ATPase | 7.7 | 17.6 | 1 | 8.4 | 19.0 | 3 | 20.91 | 20.0 | 8 |
| Q9NFA0  (PF3D7_0320700) | Signal peptidase complex subunit 2 | 6.93 | 23.46 | 2 | 1.99 | 5.03 | 1 | - | - | - |
| Q8IEI4  (PF3D7_1313000) | Ubiquitin-like protein Nedd8 | 6.6 | 35.5 | 1 | 17.2 | 60.5 | 2 | 11.85 | 77.6 | 2 |
| Q8I526  (PF3D7_1239700) | ATP-dependent zinc metalloprotease FTSH 1 | 6.40 | 15.11 | 2 | 28.76 | 30.68 | 9 | 2.42 | 16.36 | 2 |
| Q8IJR6  (PF3D7_1012900) | Autophagy-related protein 18 | 6.4 | 15.0 | 1` | 11.6 | 41.6 | 4 | 1.63 | 22.0 | 6 |
| Q8I2H3  (PF3D7_0934500) | V-type proton ATPase subunit E, putative | 5.79 | 17.45 | 1 | 5.71 | 47.23 | 1 | 5.68 | 14.04 | 1 |
| Q8ID55  (PF3D7_1365300) | Conserved Plasmodium protein, unknown function | 5.16 | 10.00 | 1 | 3.58 | 15.14 | 1 | 3.53 | 17.30 | 1 |
| Q8I713  (PF3D7_1109900) | 60S ribosomal protein L36 | 5.04 | 21.43 | 1 | 4.65 | 28.57 | 1 | 4.39 | 21.43 | 1 |
| Q8II23  (PF3D7_1134100) | Protein disulfide-isomerase, putative | 4.93 | 14.66 | 1 | 1.77 | 17.49 | 1 | 2.03 | 17.73 | 1 |
| Q8IL58  (PF3D7_1441200) | 60S ribosomal protein L1, putative | 4.17 | 17.97 | 2 | 1.72 | 32.26 | 2 | 5.59 | 15.67 | 2 |
| Q8ILE8  (PF3D7_1431700) | 60S ribosomal protein L14, putative | 3.94 | 4.24 | 1 | 20.54 | 41.21 | 4 | 26.97 | 31.52 | 6 |
| Q8IJ37  (PF3D7_1037100) | Pyruvate kinase 2 | 3.86 | 23.09 | 2 | 4.85 | 31.28 | 1 | 13.63 | 21.61 | 5 |
| Q8ILM5  (PF3D7_1422800) | Actin-related protein ARP4 | 3.6 | 5.0 | 1 | 9.4 | 13.0 | 2 | 2.13 | 3.8 | 2 |
| Q8I5G5  (PF3D7_1225200) | DNA-binding protein, putative | 3.1 | 17.4 | 1 | 6.0 | 19.0 | 4 | 1.68 | 12.0 | 1 |
| C6KTC1  (PF3D7_0628600) | DNA methyltransferase 1-associated protein 1, putative | 3.0 | 16.4 | 1 | 3.9 | 18.7 | 2 | 1.73 | 22.0 | 1 |
| C6KT13  (PF3D7_0617200) | BFR1 domain-containing protein, putative | 2.74 | 23.77 | 3 | 3.48 | 37.67 | 4 | 1.63 | 23.54 | 2 |
| Q8IE80  (PF3D7_1323700) | Glideosome associated protein with multiple membrane spans 1 | 2.39 | 5.61 | 1 | 4.54 | 22.44 | 2 | 4.08 | 5.61 | 1 |
| Q8I280  (PF3D7_0106100) | V-type proton ATPase subunit C, putative | 2.26 | 8.36 | 1 | 2.20 | 20.10 | 2 | 4.00 | 9.66 | 2 |
| Q8IER6  (PF3D7_1305000) | MCL1 domain-containing protein, putative | 2.1 | 13.1 | 1 | 11.4 | 24.7 | 7 | 6.27 | 14.0 | 2 |
| Q8IJN4  (PF3D7_1016200) | Rab3 GTPase-activating protein non-catalytic subunit, putative | 2.07 | 7.32 | 1 | - | - | - | 8.44 | 23.04 | 4 |
| Q8I0W8  (PF3D7_0513600) | Deoxyribodipyrimidine photo-lyase, putative | 2.03 | 13.12 | 1 | 1.64 | 14.38 | 1 | 1.70 | 24.08 | 1 |
| Q8IIW5  (PF3D7_1103700) | Casein kinase II beta chain | 1.94 | 10.61 | 1 | 3.85 | 20.41 | 2 | 6.29 | 14.69 | 2 |
| C6KSX7  (PF3D7_0613500) | AP-3 complex subunit beta, putative | 1.89 | 12.03 | 1 | 17.91 | 19.22 | 9 | 14.03 | 18.78 | 8 |
| Q8I334  (PF3D7_0911800) | Conserved Plasmodium protein, unknown function | 1.81 | 6.16 | 1 | - | - | - | 1.65 | 22.46 | 1 |
| Q8IAN7  (PF3D7_0803700) | Tubulin gamma chain | 1.8 | 4.2 | 1 | 11.4 | 19.1 | 4 | 4.05 | 23.2 | 2 |
| Q8IIR0  (PF11_0108) | Pre-mRNA-processing factor 6, putative | 1.8 | 22.5 | 1 | 8.4 | 27.0 | 6 | 2.67 | 26.0 | 1 |
| C6KT50  (PF3D7_0621200) | Pyridoxine biosynthesis protein PDX1 | 1.75 | 9.97 | 1 | - | - | - | 16.61 | 33.89 | 4 |
| Q8IBH9  (PF3D7_0727800) | Cation transporting ATPase, putative | 1.75 | 12.83 | 1 | 23.06 | 22.37 | 11 | 34.73 | 17.36 | 13 |
| Q8IC16  (PF3D7_0705400) | DNA replication licensing factor MCM7 | - | - | - | 4.43 | 14.25 | 2 | 3.15 | 17.05 | 3 |
| Q8I2R0  (PF3D7_0924700) | Splicing factor 3A subunit 3, putative | - | - | - | 3.30 | 22.24 | 2 | 1.64 | 19.86 | 3 |
| Q8IDN9  (PF3D7_1345800) | Conserved Plasmodium protein, unknown function | - | - | - | 10.07 | 25.45 | 5 | 2.34 | 12.78 | 1 |
| Q8IDV1  (PF3D7_1338200) | 60S ribosomal protein L6, putative | - | - | - | 20.61 | 48.42 | 6 | 6.48 | 26.70 | 2 |
| Q8IKM5  (PF3D7_1460700) | 60S ribosomal protein L27 | - | - | - | 15.66 | 40.41 | 5 | 9.38 | 35.62 | 4 |
