# Supplementary material for: Plasmodium falciparum contains functional SCF and CRL4 ubiquitin E3 ligases, and CRL4 is critical for cell division and membrane integrity
Source: PLoS Pathog. 2024 Feb 28;20(2):e1012045. doi: 10.1371/journal.ppat.1012045 (PMC10927090; doi:10.1371/journal.ppat.1012045)
Supplement: S4 Table — Shown are the Uniprot (PlasmoDB) ID, score (Sc), coverage (Co) and unique peptides (UP) for each protein present in three independent biological repeats. (DOCX) [file ppat.1012045.s016.docx]

**S4 Table.** **Proteins identified in the PfSkp1GFP immunoprecipitate.** Shown are the Uniprot (PlasmoDB) ID, score (Sc), coverage (Co) and unique peptides (UP) for each protein present in three independent biological repeats.

| **Accession ID** | **Protein** | **Experiment 1** | | | **Experiment 2** | | | **Experiment 3** | | |
| --- | --- | --- | --- | --- | --- | --- | --- | --- | --- | --- |
|  |  | **Sco** | **Cov** | **UP** | **Sco** | **Cov** | **UP** | **Sco** | **Cov** | **UP** |
| Q8ID38  (PF3D7_1367000) | Suppressor of kinetochore protein 1, putative (Skp1) | 138.3 | 24.1 | 4 | 175.7 | 43.8 | 5 | 158.7 | 22.2 | 4 |
| Q8I542  (PF3D7_1238100) | Calcyclin binding protein, putative | 1.6 | 18.0 | 1 | 2.0 | 12.7 | 1 | 18.0 | 3.5 | 1 |
| Q8I207  (PF3D7_0401800) | Plasmodium exported protein PHISTb | - | - | - | 8.1 | 21.4 | 2 | 78.5 | 39.3 | 17 |
| C6KTB1  (PF3D7_0627500) | Protein DJ-1 | 8.8 | 41.3 | 2 | 18.9 | 47.1 | 6 | 4.1 | 37.0 | 2 |
| Q8IHR4  (PF3D7_1145400) | Dynamin-like protein | 9.2 | 30.1 | 3 | 5.5 | 17.3 | 2 | 5.4 | 23.4 | 3 |
| Q8IAU5  (PF3D7_0811000) | Cullin-1, putative | 3.9 | 13.5 | 1 | 31.5 | 24.7 | 16 | - | - | - |
| Q7KQK2  (PF3D7_1211800) | PfpUB polyubiquitin | 15.8 | 76.6 | 3 | 10.7 | 35.4 | 3 | - | - | - |
| C6KT37  (PF3D7_0619700) | F-box protein FBXO1, putative | 2.2 | 27.3 | 2 | 7.8 | 31.1 | 4 | - | - | - |
| Q8I2U5  (PF3D7_0920800) | Inosine-5'-monophosphate dehydrogenase | 25.23 | 27.25 | 7 | 2.08 | 14.12 | 1 | - | - | - |
| Q8IIF0  (PF3D7_1121600) | Exported protein 1 | 9.96 | 17.28 | 2 | 6.11 | 6.79 | 2 | - | - | - |
| Q8IK02  (PF3D7_1003500) | 40S ribosomal protein S20e, putative | 3.71 | 51.69 | 1 | 4.79 | 20.34 | 1 | - | - | - |
| Q8ILV2  (PF3D7_1414300) | 60S ribosomal protein L10, putative | 1.78 | 15.07 | 1 | 2.60 | 9.13 | 1 | - | - | - |
| C6KST5  (PF3D7_0608700) | T-complex protein 1 subunit zeta | 1.61 | 18.78 | 1 | 7.72 | 25.60 | 3 | - | - | - |
| O77372  (PF3D7_0318500) | conserved Plasmodium protein | 18.45 | 30.21 | 1 | - | - | - | 8.26 | 18.25 | 1 |
| Q9U0N1  (PF3D7_0113000) | Glutamic acid-rich protein GARP | 9.34 | 17.38 | 2 | - | - | - | 21.73 | 20.95 | 4 |
| Q8ILQ9  (PF3D7_1419100) | ATP-dependent RNA helicase DDX55 | 3.35 | 31.03 | 1 | - | - | - | 3.66 | 28.63 | 1 |
| Q8IM03  (PF3D7_1409300) | DNA damage-inducible protein 1 | - | - | - | 77.2 | 38.7 | 13 | 90.8 | 40.1 | 14 |
| Q8IAN4  (PF3D7_0803400) | DNA repair and recombination protein RAD54, putative | - | - | - | 3.08 | 13.48 | 1 | 1.91 | 13.88 | 1 |
