# Supplementary material for: Plasmodium falciparum contains functional SCF and CRL4 ubiquitin E3 ligases, and CRL4 is critical for cell division and membrane integrity
Source: PLoS Pathog. 2024 Feb 28;20(2):e1012045. doi: 10.1371/journal.ppat.1012045 (PMC10927090; doi:10.1371/journal.ppat.1012045)
Supplement: S5 Table — Shown are the Uniprot (PlasmoDB) ID, score (Sco), coverage (Cov) and unique peptides (UP) for proteins present in three independent biological repeats. The characterized Plasmodium proteins are referenced. (DOCX) [file ppat.1012045.s017.docx]

**S5 Table.** **Proteins identified in the PfCullin-2/cDD_HA_ immunoprecipitate.** Shown are the Uniprot (PlasmoDB) ID, score (Sco), coverage (Cov) and unique peptides (UP) for proteins present in three independent biological repeats. The characterized *Plasmodium* proteins are referenced.

| **ID** | **Protein** | **Experiment 1** | | | **Experiment 2** | | | **Experiment 3** | | |
| --- | --- | --- | --- | --- | --- | --- | --- | --- | --- | --- |
|  |  | **Sco** | **Cov** | **UP** | **Sco** | **Cov** | **UP** | **Sco** | **Cov** | **UP** |
| C6KTD3 (PF3D7_0629800) | PfCullin-2 | 69.32 | 13.37 | 15 | 80.49 | 14.88 | 18 | 60.43 | 22.32 | 14 |
| O77380 (PF3D7_0317700) | CPSF, subunit A, putative | 59.88 | 8.43 | 12 | 67.97 | 10.42 | 17 | 68.67 | 25.61 | 32 |
| Q8ILZ2 (PF3D7_1410300) | WD repeat-containing protein, putative | 56.38 | 4.49 | 19 | 55.88 | 6.08 | 26 | 65.87 | 17.84 | 39 |
| C0H552 (PF3D7_0922800) | Uncharacterized protein | 37.72 | 3.95 | 15 | 36.87 | 4.58 | 17 | 63.72 | 17.46 | 34 |
| Q9TY95 (PF3D7_0207600) | Serine repeat antigen 5 [[1](#_ENREF_1), [2](#_ENREF_2)] | 25.97 | 9.33 | 8 | 25.36 | 10.23 | 9 | 1.81 | 8.22 | 2 |
| Q8IAK9 (PF3D7_0801000) | *Plasmodium* exported protein (PHISTc) [[3](#_ENREF_3)] | 18.41 | 6.97 | 5 | 23.41 | 10.91 | 8 | 23.83 | 8.53 | 7 |
| Q8I5D2  (PF3D7_1228600) | Merozoite surface protein 9 | 14.37 | 4.17 | 4 | 6.32 | 4.04 | 3 | 11.32 | 4.85 | 3 |
| Q8IBJ1 (PF3D7_0726500) | Ubiquitin carboxy terminal hydrolase, putative | 12.70 | 2.04 | 5 | 6.99 | 1.73 | 4 | 27.92 | 16.87 | 22 |
| Q8IEI4 (PF3D7_1313000) | Ubiquitin like protein NEDD8 [[4](#_ENREF_4)] | 11.36 | 38.16 | 2 | 10.43 | 38.16 | 2 | 7.26 | 18.42 | 1 |
| Q8IJR6 (PF3D7_1012900) | Autophagy-related protein 18 [[5-7](#_ENREF_5)] | 10.97 | 9.47 | 3 | 6.75 | 12.11 | 4 | 5.72 | 25 | 4 |
| Q8I4R5 (PF3D7_1252100) | Rhoptry neck protein 3 [[8](#_ENREF_8)] | 10.81 | 2.98 | 6 | 18.20 | 4.33 | 9 | 9.37 | 14.22 | 7 |
| Q8IAW0 (PF3D7_0812400) | Karyopherin alpha | 10.77 | 3.67 | 2 | 8.26 | 3.67 | 2 | 9.52 | 20.63 | 7 |
| Q8I5Y3 (PF3D7_1206200) | Translation initiation factor 3 subunit C, putative | 10.06 | 3.15 | 3 | 4.76 | 2.34 | 2 | 8.72 | 3.15 | 3 |
| Q8IBT2  (PF3D7_0716800) | Eukaryotic translation initiation factor 3 subunit I, putative | 9.50 | 7.03 | 1 | 14.96 | 17.13 | 3 | 16.58 | 18.04 | 3 |
| Q8I5S6  (PF3D7_1212700) | Eukaryotic translation initiation factor 3 subunit A, putative | 7.70 | 1.45 | 2 | 2.17 | 1.31 | 2 | 4.47 | 1.23 | 2 |
| Q9TY99 (PF3D7_0202000) | Knob-associated histidine-rich protein (KAHRP) [[9](#_ENREF_9)] | 7.48 | 6.12 | 3 | 15.03 | 6.12 | 3 | 26.52 | 10.86 | 4 |
| Q6ZMA7 (PF3D7_0406200) | Parasitophorous vacuole membrane protein S16 [[10](#_ENREF_10)] | 4.94 | 8.28 | 1 | 4.06 | 8.28 | 1 | 2.57 | 8.28 | 1 |
| Q8IIU7  (PF3D7_1105600) | Translocon component PTEX88 [[11](#_ENREF_11)] | 5.77 | 5.41 | 4 | 7.48 | 8.24 | 6 | - | - | - |
| Q8IC42  (PF3D7_0702500) | *Plasmodium* exported protein, unknown function | 5.59 | 11.07 | 2 | 6.96 | 4.35 | 1 | 6.50 | 4.35 | 1 |
| Q8I3T5  (PF3D7_0517300) | Serine/arginine-rich splicing factor 1 [[12](#_ENREF_12)] | 5.42 | 4.36 | 1 | 2.70 | 4.36 | 1 | 5.89 | 4.36 | 1 |
| Q8IL58  (PF3D7_1441200) | 60S ribosomal protein L1, putative | 5.25 | 9.22 | 2 | 1.84 | 3.69 | 1 | 7.07 | 13.82 | 3 |
| Q8IL56  (PF3D7_1441400) | FACT complex subunit SSRP1, putative | 4.99 | 7.91 | 3 | - | - | - | 2.46 | 3.36 | 1 |
| Q8II36  (PF3D7_1132800) | Aquaglyceroporin | 4.70 | 3.88 | 1 | 4.58 | 3.88 | 1 | 4.84 | 3.88 | 1 |
| O96258  (PF3D7_0217800) | 40S ribosomal protein S26 | 3.82 | 11.21 | 1 | 2.57 | 22.43 | 2 | - | - | - |
| Q6ZMA8  (PF3D7_0406100) | V-type proton ATPase subunit B | 3.81 | 4.05 | 1 | 3.59 | 4.05 | 1 | 4.90 | 4.05 | 1 |
| Q8IBN1 (PF3D7_0722200) | Rhoptry associated leucine zipper like protein-1 | 3.33 | 2.94 | 2 | 1.66 | 2.67 | 2 | 1.95 | 2.94 | 2 |
| Q8IIR6  (PF3D7_1108700) | Heat shock protein J2 | 3.22 | 3.15 | 1 | 5.45 | 4.81 | 2 | 7.24 | 4.81 | 2 |
| Q8I3F0  (PF3D7_0532400) | Lysine-rich membrane-associated PHISTb protein [[13](#_ENREF_13)] | 2.93 | 5.30 | 2 | 9.08 | 11.74 | 4 | 24.40 | 15.15 | 6 |
| Q8IHZ9  (PF3D7_1136500) | Casein kinase 1 [[14](#_ENREF_14)] | 2.89 | 6.50 | 2 | 3.12 | 3.72 | 1 | 6.06 | 6.50 | 2 |
| Q8IE18 (PF3D7_1330800) | RNA binding protein, putative | 2.28 | 3.36 | 1 | 6.96 | 4.87 | 2 | 5.21 | 5.37 | 2 |
| Q8IJ76  (PF3D7_1033200) | Early transcribed membrane protein 10.2 | 2.65 | 7.04 | 2 | 2.55 | 4.51 | 1 | 3.73 | 10.99 | 3 |
| Q8IE67  (PF3D7_1325100) | Phosphoribosylpyrophosphate synthetase | 2.59 | 2.75 | 1 | 10.01 | 7.78 | 3 | 9.94 | 10.07 | 4 |
| Q8IL80  (PF3D7_1438900) | Thioredoxin peroxidase 1 [[15](#_ENREF_15)] | 2.48 | 10.26 | 1 | 2.56 | 10.26 | 1 | 2.45 | 10.26 | 1 |
| C0H530  (PF3D7_0910100) | Exportin-7, putative | 2.30 | 0.83 | 1 | 2.05 | 0.83 | 1 | 1.99 | 0.83 | 1 |
| Q8IHU0  (PF3D7_1142500) | 60S ribosomal protein L28 | 2.28 | 18.11 | 2 | 4.34 | 15.75 | 2 | - | - | - |
| C0H5L3  (PF3D7_1368300) | Non-structural maintenance of chromosomes element 1, putative | 2.17 | 3.48 | 1 | - | - | - | 1.66 | 3.48 | 1 |
| C0H4H3  (PF3D7_0611700) | 60S ribosomal protein L39 | 2.15 | 19.61 | 1 | 2.27 | 19.61 | 1 | 5.33 | 19.61 | 1 |
| Q8IAU1  (PF3D7_0810600) | ATP-dependent RNA helicase DBP1, putative | 2.03 | 1.17 | 1 | 2.12 | 1.17 | 1 | 4.31 | 2.34 | 2 |
| O97292  (PF3D7_0321800) | WD repeat-containing protein, putative | 2.00 | 0.70 | 2 | 3.66 | 0.70 | 2 | - | - | - |
| O97266  (PF3D7_0315100) | Eukaryotic translation initiation factor 4E | 1.84 | 8.81 | 2 | 1.70 | 8.81 | 2 | 2.02 | 4.85 | 1 |
| Q8I3T4 (PF3D7_0517400) | FACT complex subunit SPT16, putative | 1.80 | 2.10 | 2 | 3.77 | 2.63 | 2 | 5.54 | 3.59 | 3 |
| Q8ILE8  (PF3D7_1431700) | 60S ribosomal protein L14, putative | 1.63 | 8.48 | 2 | - | - | - | 1.62 | 4.24 | 1 |
| Q8IJX3  (PF3D7_1006800) | G-strand-binding protein 2 [[16](#_ENREF_16)] | 1.63 | 3.66 | 1 | 2.07 | 7.72 | 2 | 4.87 | 7.72 | 2 |
| Q8IK07  (PF3D7_1002900) | Conserved Plasmodium protein, unknown function | - | - | - | 2.14 | 7.09 | 1 | 3.93 | 15.75 | 2 |
| O97250  (PF3D7_0307200) | 60S ribosomal protein L7, putative | - | - | - | 7.87 | 10.51 | 2 | 4.85 | 17.51 | 4 |
| C6KT34  (PF3D7_0619400) | Cell division cycle protein 48 homologue, putative [[17](#_ENREF_17)] | - | - | - | 5.18 | 5.92 | 4 | 5.30 | 5.07 | 4 |
| C0H4V4  (PF3D7_0817700) | Rhoptry neck protein 5 [[18](#_ENREF_18)] | - | - | - | 2.04 | 0.95 | 1 | 2.13 | 0.95 | 1 |
| Q8IIF0  (PF3D7_1121600) | Exported protein 1 [[19](#_ENREF_19)] | - | - | - | 4.53 | 6.79 | 2 | 4.80 | 17.90 | 3 |
| O77367 (PF3D7_0319100) | E3 ubiquitin-protein ligase RBX1, putative | 4.82 | 13.08 | 1 | 5.75 | 13.08 | 1 | - | - | - |

**References**

1. Collins CR, Hackett F, Atid J, Tan MSY, Blackman MJ. The *Plasmodium falciparum* pseudoprotease SERA5 regulates the kinetics and efficiency of malaria parasite egress from host erythrocytes. PLoS Pathog. 2017;13(7):e1006453.

2. Stallmach R, Kavishwar M, Withers-Martinez C, Hackett F, Collins CR, Howell SA, et al. *Plasmodium falciparum* SERA5 plays a non-enzymatic role in the malarial asexual blood-stage lifecycle. Mol Microbiol. 2015;96(2):368-87.

3. Nagaoka H, Kanoi BN, Morita M, Nakata T, Palacpac NMQ, Egwang TG, et al. Characterization of a *Plasmodium falciparum* PHISTc protein, PF3D7_0801000, in blood- stage malaria parasites. Parasitology International. 2021;80:102240.

4. Bhattacharjee M, Adhikari N, Sudhakar R, Rizvi Z, Das D, Palanimurugan R, et al. Characterization of *Plasmodium falciparum* NEDD8 and identification of cullins as its substrates. Scientific Reports. 2020;10(1):20220.

5. Bansal P, Tripathi A, Thakur V, Mohmmed A, Sharma P. Autophagy-Related Protein ATG18 Regulates Apicoplast Biogenesis in Apicomplexan Parasites. mBio. 2017;8(5).

6. Sudhakar R, Das D, Thanumalayan S, Gorde S, Sijwali PS. *Plasmodium falciparum* Atg18 localizes to the food vacuole via interaction with the multi-drug resistance protein 1 and phosphatidylinositol 3-phosphate. The Biochemical journal. 2021;478(9):1705-32.

7. Breglio KF, Amato R, Eastman R, Lim P, Sa JM, Guha R, et al. A single nucleotide polymorphism in the *Plasmodium falciparum* atg18 gene associates with artemisinin resistance and confers enhanced parasite survival under nutrient deprivation. Malaria journal. 2018;17(1):391.

8. Low LM, Azasi Y, Sherling ES, Garten M, Zimmerberg J, Tsuboi T, et al. Deletion of *Plasmodium falciparum* Protein RON3 Affects the Functional Translocation of Exported Proteins and Glucose Uptake. mBio. 2019;10(4).

9. Wiser MF. Knobs, Adhesion, and Severe Falciparum Malaria. Tropical medicine and infectious disease. 2023;8(7).

10. Kongkasuriyachai D, Fujioka H, Kumar N. Functional analysis of *Plasmodium falciparum* parasitophorous vacuole membrane protein (Pfs16) during gametocytogenesis and gametogenesis by targeted gene disruption. Molecular and biochemical parasitology. 2004;133(2):275-85.

11. Matz JM, Ingmundson A, Costa Nunes J, Stenzel W, Matuschewski K, Kooij TW. In Vivo Function of PTEX88 in Malaria Parasite Sequestration and Virulence. Eukaryotic cell. 2015;14(6):528-34.

12. Goyal M, Singh BK, Simantov K, Kaufman Y, Eshar S, Dzikowski R. An SR protein is essential for activating DNA repair in malaria parasites. Journal of Cell Science. 2021;134(16).

13. Proellocks NI, Herrmann S, Buckingham DW, Hanssen E, Hodges EK, Elsworth B, et al. A lysine-rich membrane-associated PHISTb protein involved in alteration of the cytoadhesive properties of *Plasmodium falciparum*-infected red blood cells. Faseb j. 2014;28(7):3103-13.

14. Batty MB, Schittenhelm RB, Dorin-Semblat D, Doerig C, Garcia-Bustos JF. Interaction of *Plasmodium falciparum* casein kinase 1 with components of host cell protein trafficking machinery. IUBMB life. 2020;72(6):1243-9.

15. Krnajski Z, Walter RD, Müller S. Isolation and functional analysis of two thioredoxin peroxidases (peroxiredoxins) from *Plasmodium falciparum*. Molecular and biochemical parasitology. 2001;113(2):303-8.

16. Niikura M, Fukutomi T, Fukui K, Inoue SI, Asahi H, Kobayashi F. G-strand binding protein 2 is involved in asexual and sexual development of *Plasmodium berghei*. Parasitol Int. 2020;76:102059.

17. Spork S, Hiss JA, Mandel K, Sommer M, Kooij TW, Chu T, et al. An unusual ERAD-like complex is targeted to the apicoplast of *Plasmodium falciparum*. Eukaryotic cell. 2009;8(8):1134-45.

18. Curtidor H, Patiño LC, Arévalo-Pinzón G, Vanegas M, Patarroyo ME, Patarroyo MA. *Plasmodium falciparum* rhoptry neck protein 5 peptides bind to human red blood cells and inhibit parasite invasion. Peptides. 2014;53:210-7.

19. Lisewski AM, Quiros JP, Mittal M, Putluri N, Sreekumar A, Haeggström JZ, et al. Potential role of *Plasmodium falciparum* exported protein 1 in the chloroquine mode of action. International Journal for Parasitology: Drugs and Drug Resistance. 2018;8(1):31-5.
