# Supplementary material for: Plasmodium falciparum contains functional SCF and CRL4 ubiquitin E3 ligases, and CRL4 is critical for cell division and membrane integrity
Source: PLoS Pathog. 2024 Feb 28;20(2):e1012045. doi: 10.1371/journal.ppat.1012045 (PMC10927090; doi:10.1371/journal.ppat.1012045)
Supplement: S6 Table — Proteins shown/predicted to be targeted to the apicoplast and mitochondria are highlighted. (DOCX) [file ppat.1012045.s018.docx]

**S6 Table. High confidence up- and down-regulated proteins in PfCullin-2-depleted trophozoites**. Proteins shown/predicted to be targeted to the apicoplast and mitochondria are highlighted.

| **Uniprot ID** | **Proteins** | **Log2-fold change** | **P-value** | **Remark** |
| --- | --- | --- | --- | --- |
| **Up-regulated** | | | | |
| Q8IEU2 | Gamete antigen 27/25 | 5.427 | 0.008 | Gametocytogenesis, sexual differentiation |
| Q8I542 | Calcyclin binding protein, putative | 5.366 | 0.021 | Regulates calcium dependent ubiquitination |
| O96230 | Acyl-CoA synthetase (ACS9) | 3.882 | 0.017 | Acetate metabolism (nuclear encoded apicoplast protein) |
| O77396 | Proteasome subunit alpha type-3, putative | 3.572 | 0.013 | Component of 20S proteasome cap |
| Q8IDG3 | Proteasome subunit alpha type-4, putative | 3.166 | 0.016 | Component of 20S proteasome cap |
| Q8I2S6 | DNA-directed RNA polymerase II subunit RPB3, putative | 3.080 | 0.028 | Transcription |
| Q8IJT9 | Eukaryotic translation initiation factor 2 subunit beta (eIF2β) | 2.911 | 0.000 | Selects AUG codon during translation initiation, direct interaction with protein phosphatase Type I |
| Q8IIG6 | Phosphoglycerate mutase, putative | 2.878 | 0.022 | Glycolysis |
| C0H4A9 | Uncharacterized protein | 2.716 | 0.046 | Unknown function |
| Q8IIL0 | Cysteine proteinase falcipain 3 | 2.549 | 0.021 | Hemoglobin metabolism |
| Q8I6U4 | Cysteine proteinase falcipain 2a | 2.548 | 0.007 | Hemoglobin metabolism |
| Q8I638 | Acyl-CoA synthetase (ACS7) | 2.440 | 0.041 | Acetate metabolism (nuclear encoded apicoplast protein) |
| Q8ILE3 | Voltage-dependent anion-selective channel protein, putative | 2.407 | 0.032 | Mediates the flow of metabolites and ions across outer mitochondrial membrane |
| Q8IEQ1 | 26S protease regulatory subunit 10B, putative | 2.346 | 0.046 | Component of 19S proteasome cap |
| Q8IEK9 | PhIL1-interacting candidate PIC5 | 2.246 | 0.048 | IMC protein, role in merozoite reorientation and invasion of host erythrocytes |
| Q7KQJ9 | Proliferating cell nuclear antigen 2 | 2.096 | 0.032 | DNA replication, DNA repair, chromatin remodelling and epigenetics |
| Q8IDF6 | Adenylosuccinate synthetase | 2.066 | 0.047 | Purine biosynthesis |
| C0H4F1 | FoP domain-containing protein, putative | 1.949 | 0.018 | Chromatin associated protein |
| Q8IJI0 | Serine/arginine-rich splicing factor 4 | 1.908 | 0.030 | Cofactor of snRNPs |
| Q8ILU3 | RNA-binding protein Nova-1, putative | 1.800 | 0.039 | RNA binding and mRNA splicing |
| Q8I2H3 | V-type proton ATPase subunit E, putative | 1.794 | 0.033 | Hydrolyses ATP, present in food vacuole proteome |
| Q8ILS0 | H/ACA ribonucleoprotein complex subunit 4, putative | 1.677 | 0.027 | rRNA processing |
| Q7KQK3 | Heat shock protein DnaJ homologue Pfj4 | 1.676 | 0.027 | Helps in parasite survival in the host |
| C6KT20 | Uncharacterized protein | 1.588 | 0.015 | Unknown function |
| O96220 | T-complex protein 1 subunit theta | 1.549 | 0.008 | Chaperone molecule, assists in protein folding upon ATP hydrolysis |
| Q8IL13 | ATP-dependent RNA helicase DDX17 | 1.536 | 0.006 | Unwinds dsRNA, crucial for parasite development |
| Q8IKP7 | Aminodeoxychorismate lyase | 1.507 | 0.032 | Folate biosynthesis, converts chorismate and glutamine |
| A0A5K1K8V8 | 60S ribosomal protein L6, putative | 1.453 | 0.041 | Component of large ribosomal subunit |
| Q8IBS3 | Serine-tRNA ligase, putative | 1.388 | 0.048 | Incorporation of L-serine during translation, (nuclear encoded apicoplast protein) |
| Q8IB66 | RNA-binding protein, putative | 1.280 | 0.049 | Binds to RNA |
| Q8I5A9 | Ras-related protein Rab-2 | 1.272 | 0.039 | Intracellular membrane trafficking, formation of transport vesicles, present in food vacuole proteome |
| Q7KQK6 | GTP-binding nuclear protein RAN/TC4 | 1.211 | 0.008 | Triggers microtubule assembly and required for chromosome segregation |
| A0A143ZWL7 | Tubulin beta chain | 1.192 | 0.034 | Constituent of microtubule and binds to GTP at beta chain |
| Q8IJZ7 | 60S ribosomal protein L13, putative | 1.172 | 0.047 | Binds RNA during translation |
| O97282 | T-complex protein 1 subunit epsilon | 1.141 | 0.048 | Chaperone molecule, assists in protein folding upon ATP hydrolysis |
| Q8II83 | Uncharacterized protein | 1.114 | 0.027 | Unknown function |
| Q8I2Q0 | Lipocalin | 1.023 | 0.007 | Mediates unidirectional heme biomineralization in *Plasmodium* (food vacuole) |
| **Down-regulated** | | | | |
| C0H5F1 | 3-hydroxyacyl-CoA dehydratase DEH | -4.2591 | 0.0004 | Long-chain fatty acid elongation, *Plasmodium* DEH is ER-localized and crucial for oocyst mitotic division during malaria transmission |
| Q8IE80 | Glideosome associated protein with multiple membrane spans 1 | -3.8283 | 8.9E-05 | Part of invasion machinery (motor locomotion) |
| C0H5K9 | Phosphatidylserine synthase | -3.7885 | 0.0304 | Catalyses synthesis of PtdSer, expresses in *Plasmodium* ER-mitochondria contact site in asexual cycle |
| O97238 | Transporter, putative | -3.6005 | 0.0210 | Transport |
| C0H548 | Uncharacterized protein | -3.5961 | 0.0317 | Putative membrane protein in Pf strain SY75 |
| Q8IIM9 | UDP-galactose transporter, putative | -3.5603 | 0.0073 | Galactosylation of proteins and lipids. |
| Q6LFN2 | Trophozoite exported protein 1 | -3.0737 | 0.0084 | A vaccine candidate, alpha helical coiled coil protein motif containing |
| O96205 | Nucleoporin NUP434, putative | -2.848 | 0.0033 | Unknown |
| P46468 | AAA family ATPase, CDC48 subfamily | -2.6662 | 0.0448 | Varied roles in division, proliferation, growth, apoptosis, necrosis, ubiquitination, membrane fusion (nuclear encoded apicoplast localized) |
| Q8IDF7 | V-type proton ATPase 21 kDa proteolipid subunit, putative | -2.599 | 0.0064 | Maintains pH of intracellular compartments and also sometimes responsible for acidifying the extracellular environment |
| Q8IBA2 | ATP-dependent RNA helicase DBP10, putative | -2.5533 | 0.0217 | Biogenesis of 60S ribosomal subunits and also required for the normal formation of 25S and 5.8S rRNAs |
| Q8IL21 | ATP-dependent RNA helicase MAK5, putative | -2.3354 | 0.0117 | Biogenesis of 60S ribosomal subunits and also required for the normal formation of 25S and 5.8S rRNAs |
| Q8IM01 | Uncharacterized protein | -2.3145 | 0.0046 | Putative membrane protein |
| Q8ID59 | DNA-directed RNA polymerases I, II, and III subunit RPABC1, putative | -1.9349 | 0.0281 | Transcription |
| Q8I3A0 | Copper-transporting ATPase | -1.9155 | 0.0177 | Maintains intracellular copper concentration by transporting copper, important for parasite fertility |
| Q8I6Z1 | Acyl-CoA synthetase (ACS5) | -1.7617 | 0.0145 | Acetate metabolism ((nuclear encoded apicoplast protein) |
| Q8I2A8 | Nucleoside transporter 4 | -1.7186 | 0.0319 | Regulates the intra- and extracellular concentrations of purine and pyrimidine nucleosides |
| Q8I5L4 | Phospholipid-transporting ATPase 2 | -1.709 | 0.0404 | Transports aminophospholipid from the outer to the inner leaflet of various membranes |
| Q8IHN4 | Antigen 332, DBL-like protein | -1.682 | 0.0057 | Duffy binding like protein |
| Q8IK09 | Uncharacterized protein | -1.5543 | 0.0094 | Unknown function |
| Q8I544 | Uncharacterized protein | -1.4124 | 0.0134 | Unknown (nuclear encoded apicoplast protein) |
| Q8I5I3 | P-type phospholipid transporter | -1.2371 | 0.0135 | Phospholipid-transporting ATPase, putative |
| Q8IJF2 | Uncharacterized protein | -1.003 | 0.0319 | Unknown (nuclear encoded apicoplast protein) |
