# Supplementary material for: Plasmodium falciparum contains functional SCF and CRL4 ubiquitin E3 ligases, and CRL4 is critical for cell division and membrane integrity
Source: PLoS Pathog. 2024 Feb 28;20(2):e1012045. doi: 10.1371/journal.ppat.1012045 (PMC10927090; doi:10.1371/journal.ppat.1012045)
Supplement: S7 Table — (DOCX) [file ppat.1012045.s019.docx]

**S7 Table. Primer used in the study.**

| **Name** | **Sequence** |
| --- | --- |
| PfRbx-F | AGAAAGATCTATGGCTGATAATATAACAAATGATA |
| PfRbxmyc-R | TAACATCTCGAGTTACAGATCCTCTTCAGAAATAAGTTTTTGCTCCAGGTCTTCCTCACTAATCAATTTCTGCTCGGTACCATCTGTGGCTTTTTGAAATTCCCA (2× Myc-tag is underlined) |
| PfSkp1-F | TAAAAGATCTATGAAAAATGATAAGATAAAATTAGTAAG |
| PfSkp1-R | TATAGGTACCTTATATCTCCACACCACCTGTTCT |
| PfCul1Fki | ATATTTAAGGGCCCCGATCCAAATTTCAACGAATGC |
| PfCul1Rki | ATTATCAACCGGTTGGTATGTAGACATAAATTTGGCTATT |
| PfCullin2F1kiFP | TTAATGCGGCCGCGGGCCCCTATCGAACATATATGTGTAAGAGA |
| PfCullin2F1kiRP | TATATTTAGGTACCCAATTCATAATGATAATTATTATTTTTGAAAAATATGAATTCTCTAC |
| PfCullin2F2F | ATCTCTCCTAGGGGTTCAAAATGATAATGAC |
| PfCullin2F2R | AATATTGGCGCCCTTCCTATTTGTTTAGCCCG |
| Cull1-FseqP3 | AGCAGAAGAATGGATAAATG |
| GFPSeqR1 | GTGCCCATTAACATCACCA |
| PcDT5U-RP | GGATGCCATGCACATGCTTAGTACACAT |
| PfCul1-3con | AACACCACATGTAGCATTACA |
| PfCullin2-5con | AGATATGTAAGTGACAAAGA |
| PfCullin2-3con | GTGTCAATATGATAAAATATATTTCCTTTCAAGCC |
| PvAc-Rseq | TAGGTATGCATACGTGAATGTACTGG |
| Hrp2-seqF | CTTTTACAATATGAACATAAAGTACAAC |
| PfAtg18-5con | CACTTAACCCGCTAAAATTGGCT |
| PfAtg18-3con | CATTATTTCGGGAAAATAAAAAAGGTAACGA |
| PfSkp1-Fexp | TAAAGGATCCATGAAAAATGATAAGATAAAATTAGTAAG |
| PfSkp1-Rexp | TATACTCGAGTATATCTCCACACCACCTGTTCT |
| PfC-Cul1-Fexp | ATATATGGATCCTCCGTCAAAATATTAAATAAGGGTTAC |
| PfC-Cul1-Rexp | TATATACTCGAGTCATTCTCCTATAATTTTTTTTAATCCTGTAAGAGCTTC |
| PfN-Cul1-F | ATATATGGATCCATGGACATTGTAAATATAAACTTTGA |
| PfN-Cul1-R | ATATATCTCGAGTCAAAAATAGGAAAATAATATATATGTTCT |
| PfCal-Fexp | ATTCGGATCCATGACGACCGAAGAAAAGATTAAC |
| PfCal-Rexp | CTTACTCGAGTTAAAAATTTGGTACGGAAAAGTCTG |
| PfFbox-Fexp | TAGCGGATCCATGGGTAATACAATTTCTGAAAGGAAACAA |
| PfFbox-Rexp | CGATCTCGAGTCAAATATCATTACCACCCTTAATTAAATAAAATAC |
